# Polymer-Based Flexible Wireless Sensors for Health Monitoring

**DOI:** 10.1007/s40820-026-02233-5

**Published:** 2026-06-08

**Authors:** Heyuan Huang, Gang Xue, Jianning Zhan, Yu Yang, Ben Jia, Zhicheng Dong, Zexing Deng, Xin Zhao

**Affiliations:** 1 School of Aeronautics, Northwestern Polytechnical University, Xi’an, 710072 People’s Republic of China; 2 Queen Mary University of London Engineering School, Northwestern Polytechnical University, Xi’an, 710072 People’s Republic of China; 3 School of Civil Aviation, Northwestern Polytechnical University, Xi’an, 710072 People’s Republic of China; 4 State Key Laboratory for Mechanical Behavior of Materials, Xi’an Jiaotong University, Xi’an, 710049 People’s Republic of China; 5 College of Materials Science and Engineering, Xi’an University of Science and Technology, Xi’an, 710054 People’s Republic of China

**Keywords:** Flexible sensors, In vivo continuous monitoring, Data processing, Low-power wireless communication, System robustness

## Abstract

Polymer-based flexible wireless sensors have demonstrated significant potential in health monitoring. However, existing studies have predominantly focused on material design, energy harvesting, and wireless communication, while research addressing signal attenuation, noise accumulation, and data distortion within wireless links remains relatively limited. Physiological signals are highly susceptible to multi-source interference during acquisition and transmission; without effective data processing and intelligent analysis strategies, monitoring outcomes may fail to meet the accuracy and stability requirements of clinical applications. To address this research gap, this review constructs a comprehensive framework encompassing signal sensing, link transmission, and intelligent processing, building on advances in flexible materials and wireless energy supply modes. Special attention is given to the critical roles of channel compensation, noise suppression, feature extraction, lightweight artificial intelligence models, and edge computing in enhancing data quality and reducing energy consumption. Furthermore, the review systematically analyzes the impact of wireless link stability and energy management on monitoring performance. By reinforcing the synergistic mechanisms between data processing and communication, this work aims to provide design principles and development directions for the next generation of high-reliability, low-power, and intelligent wireless flexible health monitoring systems.

## Highlights

- A system-level review of polymer-based flexible wireless sensors for epidermal, subcutaneous, and short-term implantable health monitoring is presented, elucidating the coupling among materials, interfaces, and wireless links.
- Materials design and application studies are systematically integrated to clarify how different material systems influence sensing performance, signal stability, and monitoring reliability.
- Key challenges and future trends are summarized, with emphasis on mitigating material-level noise, high sensitivity, multifunctional integration, and clinical translation potential.

## Graphical abstract

**Figure:**
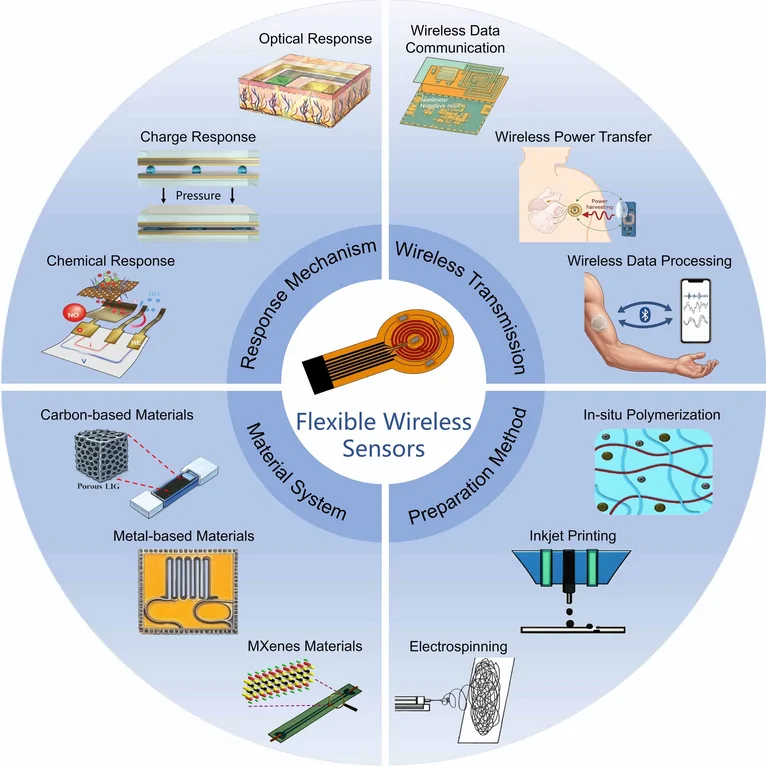

## Introduction

**Fig. 1:**
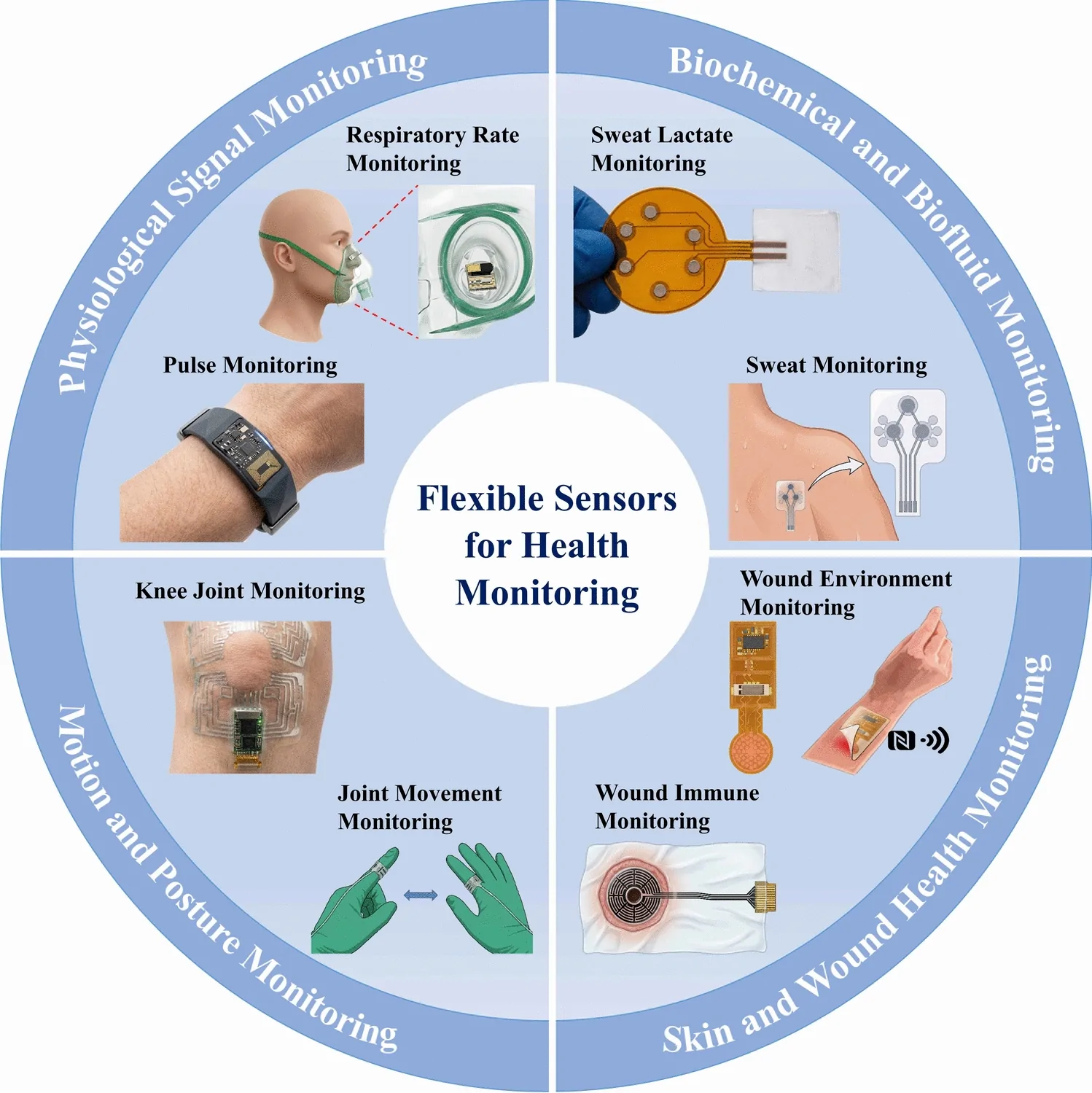
Applications of flexible sensors in multidimensional health monitoring, including physiological signals, biochemicals and biofluids, skin and wound health, and motion and posture

The global surge in chronic illnesses, rapid demographic aging, and the growing preference for home-based care highlight the critical inadequacy of hospital-centered, episodic physiological monitoring. Conventional medical instrumentation predominantly relies on bulky, rigid, and wired systems designed for snapshot measurements taken days or weeks apart. This intermittent approach is intrinsically ill-equipped to capture dynamic, high-frequency physiological fluctuations that unfold within seconds, thereby hindering early diagnosis, longitudinal tracking, and proactive intervention for patients requiring continuous care. To address these intrinsic limitations, the convergence of flexible electronics and wireless technology has emerged as a transformative trend in continuous healthcare. Flexible sensors feature mechanical compliance, tissue-like modulus, and ultra-thin form factors, allowing them to conform intimately to the human epidermis [1–3]. This mechanical congruence eliminates the motion artifacts, interfacial degradation, and wearer discomfort typically associated with rigid devices [4–6]. Crucially, when seamlessly integrated with ultra-low-power radios, embedded micro-controllers, and wireless energy-harvesting modules, these skin-mounted systems completely remove physical tethers [7–9]. This wireless-flexible paradigm enables unobtrusive, real-time tracking of multidimensional physiological data across diverse daily scenarios, ranging from natural sleep to vigorous exercise. By delivering high-fidelity continuous data streams, these systems provide a highly reliable foundation for longitudinal disease management and personalized therapeutics [10–12]. Representative embodiments are illustrated in Fig. 1.

While polymer-based flexible wireless sensors have garnered considerable research interest, existing review articles typically suffer from certain limitations. Most studies focus on isolated aspects, such as single-material platforms [13], device-level sensing mechanisms that lack adequate emphasis on wireless system-level co-design [14], or broad technical classifications of manufacturing processes without systematic comparison of interfacial stability and wireless integration compatibility [15]. Importantly, previous reviews often treat wireless transmission as an independent and disconnected module. They rarely address the inevitable signal degradation, including noise contamination, link attenuation, and coupling distortion, that arises from multi-interface propagation, nor do they systematically analyze signal processing and compensation strategies within a unified framework alongside material properties.

To bridge this long-standing gap between materials science and systems engineering, this review adopts an end-to-end framework encompassing sensing mechanisms, wireless systems, manufacturing strategies, materials, and applications. Specifically, this review highlights four principal contributions. First, we reconstruct the data acquisition and analysis workflow, clarifying how molecular-level properties and polymer-interfacial impedance propagate into communication reliability and ultimately determine diagnostic credibility. Second, we break the traditional limitation of isolating fabrication from system performance by deeply analyzing how manufacturing methods systematically influence both core electrical properties and wireless transmission capabilities. Third, we systematically integrate and compare diverse functional materials within polymeric matrices, including carbon-based fillers, metallic nanostructures, intrinsically functional polymers, and 2D MXene networks, correlating their filler–matrix microstructures with overall sensing performance and signal fidelity. Finally, to enhance clinical translation, we provide an in-depth benchmarking between polymer-based flexible wireless sensors and clinical standard devices. This outlines future directions for achieving high sensitivity and multifunctional operation under stringent battery-free power budgets, ultimately providing a comprehensive reference for translating laboratory prototypes into regulatory-qualified, clinically deployable monitors.

## Response Mechanisms

### Optical Response

Optical response mechanisms originate from the reversible modulation of material optical properties, including absorption, scattering, and emission, in response to external stimuli. These phenomena are intrinsically governed by electronic energy-level transitions initiated through light-matter interactions [16, 17]. Transduction proceeds via spectral shifts, visible color changes, or variations in fluorescence and phosphorescence intensity. Signal generation follows two main routes. Direct routes rely on the analyte’s intrinsic optical changes. Alternatively, reagent-mediated routes use dyes, molecular probes, or nanostructures to amplify the signal. Reagent-mediated methods offer exceptional sensitivity to subtle chemical changes, while direct methods provide rapid and intuitive visual readouts. Due to their high sensitivity and capacity for remote, non-contact monitoring, optical response strategies are broadly applicable across diverse domains of physiological analysis.

**Fig. 2:**
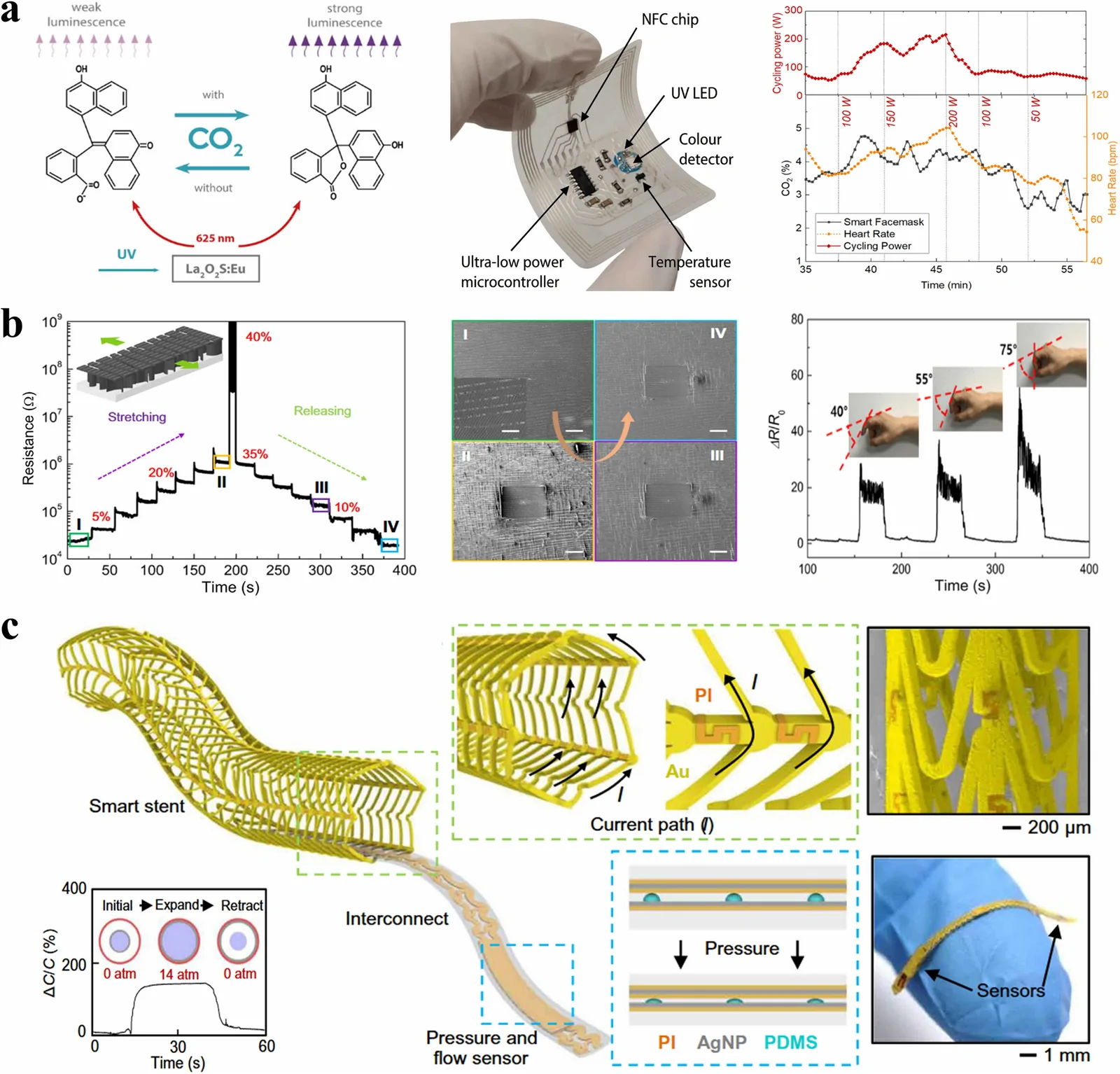
**a** Wearable CO_2_ monitoring system integrated into a smart facemask. The panels show the CO_2_ detection mechanism based on phosphorescence emission intensity modulation (left), a photograph of the flexible sensing patch with integrated components (middle), and real-time monitoring of CO_2_ concentration and heart rate during exercise (right) [18]. Reproduced with permission. Copyright 2022, Nature Communications. **b** Resistive strain sensor based on structural design. The panels illustrate the step-wise resistance response and corresponding microstructural evolution during stretching and releasing cycles (left and middle), and its application in monitoring finger joint bending at specific angles (right) [21]. Reproduced with permission. Copyright 2022, npj Flexible Electronics. **c** Integrated smart stent system. The schematics display the structural design and current path of the smart stent (top), the capacitance modulation mechanism of the integrated pressure and flow sensor (bottom right), and the sensor’s capacitance response during mechanical expansion (bottom left) [22]. Reproduced with permission. Copyright 2022, Science Advances

Within the domain of respiratory gas surveillance, Escobedo and co-workers [18] (Fig. 2a) introduced an intelligent FFP2 respirator that operates through a reagent-mediated optical pathway. The device transduces carbon dioxide fluctuations into readable signals by modulating the emission of an inorganic phosphor whose luminescence is altered by CO_2_-driven pH shifts; an acid–base indicator subsequently intensifies this modulation through an inner-filter interaction, yielding concurrent spectral and colorimetric outputs. The architecture is expressly tailored for deployment within the respiratory microenvironment of the facemask, where gaseous gradients are steep and analytical sensitivity must remain high, and can be coupled to a near-field communication wireless power module to sustain the uninterrupted acquisition of data. Conversely, for the chemical profiling of perspiration, Zhang et al. [19] devised a stretchable, superhydrophilic colorimetric membrane governed by a direct optical principle. Upon sweat ingress, embedded pH dyes and ion-chelating chromophores undergo absorbance shifts that translate into discernible hue variations proportional to electrolyte concentration and acidity. The construct conforms to expansive skin areas and withstands vigorous perspiration during physical exertion, while smartphone-based imaging and color calibration deliver immediate post-exercise electrolyte evaluation.

Absorption- and emission-based optical transduction furnishes a contact-free, highly sensitive route to the quantification of respiratory gases and biofluids such as perspiration, translating molecular recognition events almost instantaneously into intuitive spectral shifts or intensity variations. Yet the same modality is constrained by photobleaching, fluorescence decay, and the finite chemical robustness of organic dyes, while the requisite light sources, filters, and collimation optics enlarge the system complexity [20]. In flexible or wearable formats, minute bending or stretching displaces optical axes, and interfacial fouling or non-specific sweat ingress introduces background drift that erodes measurement fidelity. Prospective advances are therefore expected to focus on inherently stable photosensitive architectures, such as metal organic frameworks, two-dimensional lattices, and quantum dot assemblies, which can prolong emission lifetime and enhance quantum yield. Concurrently, low-power optoelectronic readout schemes married to adaptive calibration algorithms should yield more integrated, energy-frugal, and mechanically resilient optical sensing platforms.

### Charge Transport Response

Charge transduction sensing is prevalent in flexible and wearable architectures because mechanical stimuli naturally disrupt carrier motion, spatial distribution or internal dipole moments. To provide a rigorous physical framework, the transduction pathways in flexible sensors can be categorized into four primary electromechanical mechanisms: Piezoresistive, capacitive, piezoelectric, and triboelectric responses.

#### Piezoresistive Sensing

Piezoresistivity relies on the modulation of electrical resistance under applied strain or pressure. In flexible conductive composites (e.g., semiconductor matrices or percolative filler networks), the physical mechanism is governed by the reconfiguration of the conductive network. Deformation alters the inter-particle distances, which dramatically affects the contact resistance and the probability of quantum‐mechanical tunneling between adjacent conductive fillers. Na et al. [21] (Fig. 2b) ingeniously utilized these physics by translating skeletal motion into an electrical trace through a vertical graphene (VG) film governed by crack-opening statistics. Under tensile load, the VG lattice parts along sub-micron fissures, switching the dominant carrier pathway from percolative networks to quantum‐mechanical tunneling, thereby elevating the gauge factor beyond 5 × 10^3^. Conversely, in bulk electrolytes and ionogels, piezoresistivity is governed by ionic mobility. For example, Pei et al. [23] demonstrated a zwitterionic hydrogel that sustains a robust resistance change through the reversible modulation of ionic migration channels, accommodating extreme elongations (up to 670%) for subdermal implantable monitoring. However, piezoresistive architectures present inherent mechanical challenges. The intrinsic viscoelastic creep of elastomer matrices and the structural fatigue of conductive networks under dynamic loading often lead to mechanical hysteresis and baseline drift, necessitating further structural engineering.

#### Capacitive Sensing

Capacitive sensors operate on the principle of a parallel-plate capacitor. Mechanical stimuli induce structural deformations that alter the overlapping area, the distance between the electrodes, or the effective relative permittivity of the dielectric layer. To enhance sensitivity, introducing micro-textures (e.g., pyramids or porous sponges) into the dielectric layer is highly effective, as the compression displaces trapped air (low permittivity) with the elastomer matrix (higher permittivity). Herbert et al. [22] (Fig. 2c) abandoned crack physics in favor of this dielectric strategy: A micro-textured polydimethylsiloxane (PDMS) layer functions as a compressible spacer. Applied pressure reduces the air fraction within the texture, increases the effective permittivity, and produces a capacitive sensitivity of 0.013 kPa^−1^ with a millisecond response. Coupled inductively to an implanted stent, this architecture provides a linear, low-drift readout of quasi-static pressures and pulsatile flows. Despite their high linearity, capacitive sensors are intrinsically vulnerable to parasitic capacitance and moisture. Because biofluids possess a drastically higher relative permittivity than typical elastomers, fluid ingress can severely overwrite the baseline, making advanced encapsulation strictly necessary.

#### Piezoelectric Sensing

Unlike piezoresistive and capacitive modes that require an external power supply, piezoelectric sensing is an active mechanism based on the generation of polarization charges. It is intrinsically rooted in the non-centrosymmetric crystal structures of specific materials (e.g., ZnO and lead zirconate titanate). When subjected to mechanical stress, the crystal lattice deforms, shifting the centers of positive and negative charges. This broken symmetry induces a net dipole moment and a transient potential difference across the material. Due to the rapid generation and dissipation of charges, piezoelectric flexible sensors are exceptionally well-suited for capturing highly dynamic physiological signals. For instance, recent advancements have successfully integrated these active sensors into wireless body area networks. As demonstrated in a recent study [24], researchers engineered a self-powered wireless piezoelectric sensor by embedding high-k barium titanate BaTiO_3_ nanoparticles into a highly stretchable polyamide elastomer matrix. The specific structural design of this device amplifies local stress transfer to the BaTiO_3_ fillers. This allows the soft, wearable platform to effectively convert subtle biomechanical movements, such as wrist flexion and gait dynamics, into distinct high-voltage signals. Coupled with a customized low-power Bluetooth module, it transmits these dynamic waveforms wirelessly to mobile terminals, enabling continuous, machine-learning-assisted kinematic monitoring. Nevertheless, a fundamental limitation of piezoelectric sensors is their inherent inability to measure static or quasi-static pressures, as the generated polarization charges rapidly leak through the internal resistance of the material and external circuits. Furthermore, matching their substantial intrinsic impedance with conventional electronics poses a significant system-level challenge. Ensuring clinical-grade versatility demands the integration of specialized charge amplifiers, optimized dielectric barriers to extend charge retention times, and hybrid sensing modalities to capture both dynamic and static physiological events.

#### Triboelectric Sensing

Triboelectric sensing represents another self-powered active paradigm, driven by the coupling effect of contact electrification and electrostatic induction. When two distinct flexible materials with different electron affinities physically contact and separate under external pressure, an asymmetrical charge transfer occurs at their interface. The subsequent mechanical separation distances these polarized surfaces, driving free electrons to flow through an external circuit via Maxwell’s displacement current to balance the local potential. This mechanism transforms biomechanical energy directly into high-voltage readable pulses. Leveraging this principle, recent breakthroughs have realized completely untethered sensing paradigms. For example, Xie et al. [25] developed a highly conformable triboelectric tactile sensor array utilizing liquid metal electrodes patterned on soft elastomeric substrates. When external pressure is applied, the contact electrification between the textured tribo-layers and human skin generates localized charge distributions. By coupling these dynamic triboelectric signals with customized wireless transmitters and neuromorphic computing algorithms, this self-powered system achieves untethered, high-spatiotemporal mapping of complex tactile stimuli, showcasing the tremendous potential of triboelectric mechanisms in next-generation wireless healthcare interfaces. Despite their high-output voltages, the primary bottleneck for triboelectric nanogenerators (TENGs) lies in severe mechanical wear and the screening of surface charges by environmental humidity, which drastically reduces long-term electrical output.

#### Comparison and Future Perspectives

While the four aforementioned charge-transduction mechanisms exhibit distinct physical origins, they collectively enable high-resolution sensing of physical stimuli, featuring microsecond responses, microwatt power drain, and broad material compatibility. However, selecting the optimal mechanism requires balancing their inherent trade-offs. For instance, piezoresistive and capacitive modes excel in static and quasi-static measurements but require continuous external power. Conversely, piezoelectric and triboelectric paradigms offer self-powered, highly dynamic sensing capabilities but intrinsically struggle with static pressure retention and impedance mismatch. Beyond these mechanism-specific characteristics, all charge-transduction sensors face shared, critical vulnerabilities when deployed in practical environments. These charge carriers are highly susceptible to extrinsic interference. Fluctuations in ambient temperature and humidity, coupled with sweat accumulation and natural skin exfoliation, severely compromise the sensor–skin interface [26]. These factors disrupt internal charge transport, causing baseline drift and rapid signal failure that often obscure the intended physiological measurements. Intrinsically, devices relying on complex nanomaterial architectures face additional hurdles, primarily limited wafer-level reproducibility and progressive structural fatigue over thousands of operational cycles [27, 28].

Achieving reliable long-term operation for epidermal or implanted sensors requires mitigating key degradation pathways like moisture ingress, ionic perturbation, and mechanical wear. Suppressing biofluid ingress without polarization-induced band bending requires co-designing mechanically compliant encapsulants with interfacial adhesion promoters. Concurrently, stabilizing the conductive percolation network against thermal and ionic perturbations relies on targeted defect engineering, surface dipole tuning, and hierarchical nanostructuring. To overcome mechanical wear, transitioning to fatigue-immune films requires scalable manufacturing methods like roll-to-roll metallization and laser-induced forward transfer, combined with solution-processed self-healing polymers capable of recovering conductivity after extreme deformation (> 1,000% strain or > 10,000 bending cycles). Ultimately, long-term stability depends on monolithic system integration. Coupling robust transducers with sub-microwatt analog front ends enables localized digitization and drift correction. Embedding these components within edge-learning architectures alongside multimodal reference sensors establishes a closed calibration loop, ensuring continuous and clinically stable physiological monitoring.

### Specific Chemical Response

The chemical response mechanism of flexible sensors depends on specific molecular interactions to directly translate analyte recognition into quantifiable signals. Target molecules diffuse into the sensing structure and undergo chemical transformations including enzymatic reactions, redox processes, and host–guest complexation. These reactions modify interfacial charge, ionic concentrations, or dipole configurations [29]. Such physical and chemical perturbations are then converted into electrical signals through electrochemical, capacitive, field-effect, or impedimetric transduction pathways. By coupling chemical recognition events with electrical readout, this integrated reaction-transduction cascade delivers reliable selectivity and supports continuous, highly specific health monitoring in complex physiological media.

**Fig. 3:**
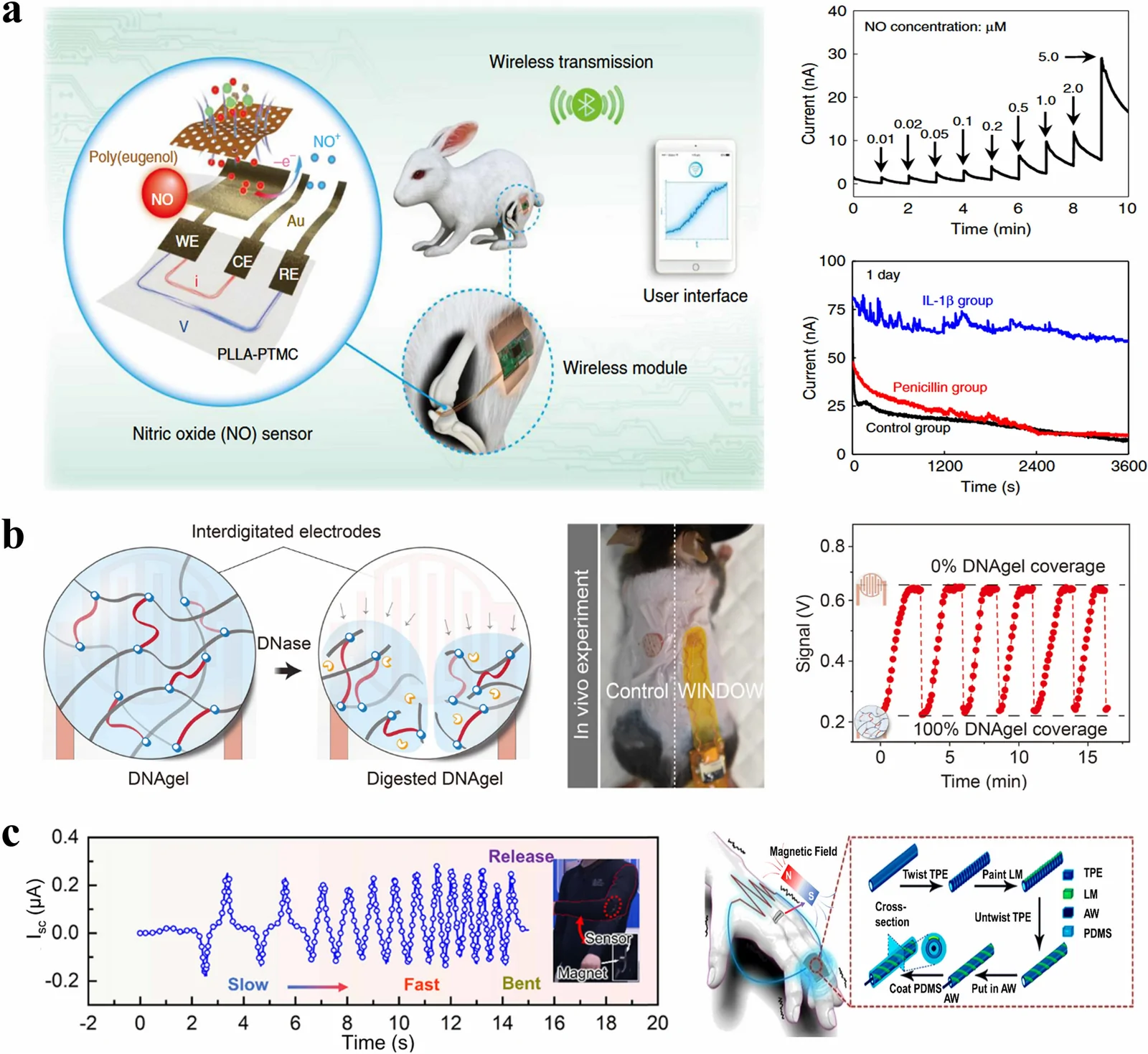
**a** Specific oxidation of NO mediated by a poly(eugenol) permselective membrane for highly selective NO detection and wireless real-time monitoring in the rabbit joint cavity [30]. Reproduced with permission. Copyright 2020, Nature Communications. **b** DNA hydrogel undergoes selective enzymatic degradation in response to deoxyribonucleases, leading to decreased gel coverage and subsequent changes in dielectric/electrical signals, which was successfully applied for in vivo monitoring in mice [31]. Reproduced with permission. Copyright 2021, Science Advances. **c** Under an external magnetic field, geometric deformation of flexible coils based on a liquid metal coating leads to changes in magnetic flux, thereby generating an induced electromotive force (recorded as short-circuit current, I_sc_) for effective sensing of motion speed (slow, fast) and bending strain signals [34]. Reproduced with permission. Copyright 2022, Nano Energy

Li et al. [30] (Fig. 3a) interrogated nitric-oxide flux in biofluids through a chemoselective anodic route. A poly(eugenol) membrane, engineered for hydrophobic rejection, charge sieving, and size exclusion, steers nitric oxide (NO) toward a pre-oxidation state at the working electrode while denying access to ascorbate, nitrite, and catecholamines. Coupled to a potentiostat-on-flex and an inductive power coil, the device streams sub-micromolar NO transients from macrophages, cartilage explants, and murine joints, affording an early electrochemical signature of inflammation and cartilage catabolism. Xiong et al. [31] (Fig. 3b) exchanged redox chemistry for enzymatic dielectrometry: A DNA hydrogel cast between interdigitated electrodes functions as both recognition element and dielectric medium. Pathogen-secreted deoxyribonucleases cleave the network, elevating water content and lowering the relative permittivity; the capacitive decrement is inductively linked to a reader coil outside the incubator, permitting label-free detection of colony-forming units within the first hour of infection while the gel’s porosity inherently rejects proteins and cells. Liu et al. [32] dispensed with external power altogether by letting sweat drive the chemistry. Lactate oxidized at a screen-printed lactate oxidase anode liberates electrons and generates lactate acid–base equivalents that act as internal electrolyte; the resulting 0.3–0.5 V sweat battery powers a three-electrode microfluidic strip that reports pH, glucose, and Na^+^ via chronopotentiometric pulses transmitted by Bluetooth. The same convective flow that fuels the cell continuously flushes surfactants and skin debris, yielding a self-cleaning, self-powered platform that survives days of profuse secretion. Together, these examples illustrate how deliberate molecular recognition, whether oxidative, enzymatic, or metabolic in nature, can be integrated with flexible, wireless, and even energy autonomous architectures to enable uninterrupted chemical surveillance in complex biological environments.

Polymer-based flexible wireless sensors based on specific chemical transformations achieve selective, highly sensitive detection in complex biofluids by converting molecular events into wireless readable electrical/dielectric signals via specific target-sensing material reactions. However, their core recognition units are vulnerable to pH, temperature, and ionic strength fluctuations, and long-term exposure to biofluids or environmental pollutants easily causes structural relaxation, activity loss, or non-specific adsorption of enzymes, aptamers, and molecular receptors, further triggering baseline drift and selectivity degradation, with inherent environmental sensitivity and poor long-term stability remaining the key bottlenecks for practical application [33]. Future research should focus on constructing highly stable recognition interfaces via robust recognition elements and antifouling modification, integrating flexible microfluidics for efficient sample manipulation and signal stability enhancement, and combining low-power wireless circuits with embedded calibration algorithms to compensate for environmental and aging effects, to support reliable long-term continuous monitoring.

### Magnetic Response

In the field of polymer-based flexible wireless sensors, magnetic sensing mechanisms have evolved into a crucial passive signal transduction strategy, facilitating long-range readout without requiring complex circuit configurations. The fundamental principle of this approach hinges on alterations in the magnetic properties of magnetic materials in response to external stimuli, such as pressure, tension, or deformation. Specifically, ferromagnetic or magnetoelastic materials exhibit magnetostrictive effects under mechanical loading; this phenomenon induces the rearrangement of magnetic domains and gives rise to measurable variations in magnetization vectors [35]. Flux-based sensors take measurable changes in magnetic flux or magnetization as their transduction foundation, a design that enables reliable and highly sensitive real-time sensing of physiological activities including cardiovascular, respiratory, and muscular signals.

In the acquisition of stretchable strain signals, magnetic sensing strategies are primarily categorized into two types: Self-inductive coil structures and magnetic hydrogel external magnetic sensor coupled structures. Li et al. [34] (Fig. 3c) proposed a liquid metal helical coil sensor, which leverages the geometric deformation of the coil under tensile strain to induce variations in magnetic flux, thereby generating an electromotive force for self-powered strain signal transduction. The response efficiency of this sensor can be further improved by introducing gradient magnetic fields or high-permeability cobalt-based amorphous wires. This system is applicable for real-time monitoring of finger bending, wrist movements, and hand tremors (1–5 Hz), demonstrating potential applications in Parkinson’s disease rehabilitation and wearable electronic skin. By contrast, Zhang et al. [36] developed a wireless flexible strain sensor using GelMA/Fe_3_O_4_ magnetic hydrogel. In this design, local changes in magnetization and magnetic flux density caused by the rearrangement of Fe_3_O_4_ nanoparticles under small strains are detected by an external magnetic sensor, enabling passive and contact free signal acquisition. In highly dynamic environments, traditional passive sensing mechanisms often face limitations in capturing multifaceted fluidic information. To address this, magnetic soft composites can be leveraged to establish actuation-enhanced active sensing paradigms. Notably, Han et al. [37] developed magnetic artificial cilia arrays that dynamically interact with surrounding fluids through remote magnetic actuation. Coupling these soft robotic dynamic interactions with machine-learning algorithms enables the system to quantitatively recognize and decouple complex environmental cues, such as localized liquid viscosity and flow dynamics. This expands the capabilities of flexible sensors in complex biological fluids.

Flux-based magnetic sensing mechanisms achieve low-power, long-range wireless detection via magnetic domain rearrangement and magnetization modulation, with inherent insensitivity to electrical noise and conductive media, robust environmental adaptability, and compatibility with flexible conductive materials for highly sensitive monitoring of subtle mechanical deformations. However, they still face critical bottlenecks including signal hysteresis and drift from magnetic hysteresis and nonlinear effects, as well as large variability in magnetomechanical coupling across diverse material systems [38]. Future research should focus on optimizing magnetomechanical coupling via carbon nanotube (CNT)-reinforced magnetic composites, 2D magnetic materials, and high-performance amorphous magnetic alloys to boost sensitivity, long-term stability, and low-power performance, alongside limiting magnetic field strength within safe thresholds and mitigating environmental magnetic interference for reliable wearable and epidermal applications.

### Multimodal Synergistic Response

A single stimulus input is frequently inadequate for comprehensive characterization of complex physiological or environmental states. Multimodal response mechanisms integrate distinguishable stimulus responsive features into material systems, interfacial charge behaviors, or hierarchical microstructures, allowing different physical quantities such as strain, pressure, and temperature, as well as chemical parameters including pH value, ion concentration, and gas composition, to induce either synergistic or decoupled responses within a single sensing unit. Through the rational utilization of composite materials, multi-channel charge networks and multi-layered structural designs, multimodal sensors exhibit the potential to achieve simultaneous multi-parameter monitoring without augmenting device volume, thereby enhancing information density and environmental adaptability. Nevertheless, translating these conceptual advantages into practical flexible systems is often hindered by complex signal coupling and environmental interference. To address these practical challenges, beyond orthogonalization strategies implemented at the material and structural levels, multimodal systems are capable of employing data fusion and machine learning algorithms to accomplish mode separation and feature extraction in the signal domain, which further improves the interpretability and robustness of multi-source information.

In the field of multimodal sensing research, Yu et al. [39] fabricated a three-dimensional helical structure using flexible Bi_2_Te_3_ thin films, enabling simultaneous detection of temperature and pressure. Modal orthogonality in this system stems from distinct physical mechanisms: Temperature signals are generated through the thermoelectric effect, while pressure signals originate from the negative piezoresistive effect of the helical structure. Given the independent operation of these two pathways, decoupled outputs can be achieved without the need for composite materials. This characteristic renders the system suitable for array-based mapping of pressure temperature distributions. By contrast, Baines et al. [40] proposed the ChromoSense system, where modal orthogonality is accomplished via optical multi-channel decoupling. Functional dye elastomers undergo color changes under bending and exhibit alterations in light intensity under stretching or compression, while temperature modulates the optical properties of the dyes. Consequently, chromaticity, light intensity, and temperature responses correspond to separate optical readout dimensions, realizing intrinsic three modal decoupling. This approach eliminates the requirement for conductive materials and relies exclusively on optical signals, thereby conferring portability and flexibility. Gao et al. [41] utilized a single Ti_3_C_2_T_x_ MXene to construct a three-dimensional porous elastic structure, with modal orthogonality primarily derived from differences in the material’s electrical properties and structural deformation pathways. The temperature response is based on the thermosensitive conductivity of MXene, whereas the pressure response results from resistance changes induced by the compressibility of the porous structure. Due to the independence of these two signals along the charge transport pathways, there is no crosstalk between temperature and pressure detection. This feature makes the system well suited for applications in electronic skin and biomimetic tactile recognition. Furthermore, Dai et al. [42] proposed a novel piezoelectric-electrocatalytic flexible sensor for the simultaneous monitoring of pulse and glucose levels. The core breakthrough lies in demonstrating the modulatory role of the piezotronic effect: The dynamic polarization electric field generated by mechanical deformation is not only extracted as high-quality pulse signals but also acts as an additional driving force to facilitate interfacial carrier transfer, thereby drastically enhancing the catalytic efficiency for non-enzymatic glucose sensing.

While multimodal synergistic mechanisms enhance information density through simultaneous multi-parameter detection, their real-world flexible applications face significant challenges, notably signal crosstalk, material limitations, and system integration complexity. Signal crosstalk arising from overlapping transport pathways or coupled deformations can be addressed systematically by introducing physical isolation microstructures, frequency-domain multiplexing, and machine learning for nonlinear signal decoupling. Additionally, flexible materials are susceptible to degradation such as microcracking or plastic deformation under repeated loading. Overcoming these limitations requires the development of self-healing elastomers and stress-absorbing dynamic architectures alongside robust calibration strategies to combat environmental and aging interferences. Furthermore, from an engineering perspective, multiple output channels considerably increase data bandwidth and system integration complexity. Tackling these integration challenges demands monolithic integration with flexible printed circuits (FPCBs) and front-end in-sensor computing or data compression to reduce communication loads and enhance energy efficiency. Ultimately, future advancements will rely on synergistic material-structural designs to improve signal orthogonality, coupled with artificial intelligence approaches for robust feature extraction and anomaly detection in complex, high-dimensional monitoring environments.

Table 1 summarizes representative recent studies on polymer-based flexible wireless sensors employing different response mechanisms. It provides a systematic comparison of the underlying mechanisms, their advantages and limitations, response parameters, sensing range, and key features, serving as a reference for sensor design optimization.

**Table 1:** Summary of representative polymer-based flexible wireless sensors based on different sensing mechanisms

Mechanism Category	Specific Sensing Mechanism	Advantages and Limitations	Response Parameter	Sensing Range	Key Features	Refs
Optical	Posture: Bending-altered light path; selective dye absorption pH: Sweat-induced Rhodamine B conformation change; fluorescence variation	Advantages: Single-fiber dual detection (physicochemical); biocompatible Limitations: Posture-pH signal crosstalk; two-step decoupling calibration required	Posture: Visible light color/wavelength shift (RGB/chromaticity) pH: UV fluorescence intensity variation	Posture: 0–90° for a single bending segment pH: 4–8	Detection Limit (posture): 0.02 mm^−1^ Linear regression R^2^ (pH): 0.993 Optical attenuation: -0.6 dB cm^−1^ Response time: 10 ms	[43]
	Compressed porous red PL layer; increased green TIEL transmittance; real-time red-to-green transition	Advantages: High sensitivity and resolution; display-free visual pressure mapping Limitations: Integrated TENG matrix needed for quantitative electrical signals	Optical color variation (green/red intensity ratio) and electrical voltage signals	10 kPa–2.4 MPa	Detection Limit: 10 kPa Sensitivity: > 190 kPa^−1^ overall Response Time: < 10 ms Spatial Resolution: 500 μm	[44]
Charge Transport	Capacitive; capillary water transport into yarn grooves increases dielectric constant and capacitance	Advantages: Highly sensitive, breathable, battery-free wireless respiratory monitoring Limitations: Slight capacitance drift under prolonged extreme bending	Outputs electrical capacitance and wireless resonance frequency shifts	6%–97% Relative Humidity (RH)	Sensitivity: 82.40 pF/%RH Response/Recovery time: 3.5/4 s Stability: Highly stable, with only a 2.1% capacitance increase after 1000 bending cycles	[45]
	Resistive; water physisorption on graphene-carbon increases resistance	Advantages: Flexible, low-cost, disposable, fast-responding Limitations: Paper substrate limits its use to non-wet environments; wireless requires ~ 3 V external power	Measured as electrical resistance (Ω), change in resistance (ΔR), or percentage response	25%RH–91.7%RH	Sensitivity: ~ 12.4 Ω/%RH Response/Recovery: ~ 31/ ~ 8 s (chamber); ~ 4/ ~ 6 s (respiration) Stability: > 100 cycles repeatability; > 4 months lifespan	[46]
	Resistive; strain-induced 3D graphene network disconnection (resistance surge); Velcro-like structure re-adhesion for recovery	Advantages: Perfect performance recovery after severe overload breakage Limitations: Narrow high-sensitivity range (2.5%); performance highly dependent on elongation direction	Outputs resistance (Ω) and relative resistance variation (ΔR/R_0_)	Perpendicular to cracks: 0–2.5%; Parallel to cracks: 0–40%	Sensitivity (GF): > 5,000 at 2% strain (VGS-V) Response/Recovery time: ~ 200/ ~ 100 ms Durability: > 10,000 consecutive stretching-releasing cycles	[47]
Chemical	Glucose-induced pH drop causes hydrogel shrinkage; altered antenna capacitance; shifted RF resonant frequency and magnitude	Advantages: Battery-free, wireless, minimally invasive; single-step continuous glucose monitoring; high specificity Limitations: Signal strength highly sensitive to antenna alignment and vertical separation	RF signal reflection (S11 parameter), specifically shifts in resonant frequency (MHz) and magnitude (dB)	0–18 mM glucose concentration	Sensitivity: 17 MHz/mM Accuracy: ~ 91% (based on Clarke Error Grid) Optimal Reading Distance: 2.5 mm	[48]
	Electrocatalytic oxidation of sweat metabolites by conductive Ni_3_HHTP_2_ MOF; Faradaic current generation	Advantages: High breathability; wet-adhesion on skin; stable non-enzymatic performance Limitations: Relies on rigid PCB module for wireless data transmission	Oxidation current (μA) measured via chronoamperometry and differential pulse voltammetry	Vitamin C: 10–1190 μM Uric acid: 50–250 μM	Breathability (WVTR): 21.3 g‧m^−2^‧h^−1^ Wet Adhesion: ~ 27 kPa Operating Potential: ~ 0.001 V for Vitamin C	[49]
	Lactate oxidation via Prussian blue mediator; proportional Faradaic current	Advantages: Zero-power, continuous resting sweat monitoring Limitations: Dilution and skewed readings from intense exercise sweating	Current response (µA) measured via chronoamperometry	0–15 mM (linear dynamic range)	Sensitivity: 90 nA·mM^−1^·mm^−2^ (in vitro) Limit of Detection (LOD): 350 nM Power Consumption: 706 µW (at 10 Hz sampling rate)	[50]
Magnetic	Stress-altered micromagnet spacing/dipoles; liquid–metal coil induction	Advantages: Highly stretchable, intrinsically waterproof, zero external magnetic field Limitations: Relies on dynamic deformation; output vs. softness trade-off	Magnetic flux density changes; open-circuit voltage and short-circuit current	Minute vibrations to 400 kPa stress; up to 190% tensile strain	Coupling factor: 7.19 × 10^–8^ T Pa^−1^ Internal impedance: ~ 30 ohms Current density: 4.27 mA cm^−2^	[51]
	Deformation-altered magnetic dipoles; conductive yarn induction	Advantages: Intrinsically waterproof, high tissue compatibility, zero external magnetic field Limitations: Output highly dependent on textile weaving and folding patterns	Magnetic field variations (ΔB) physically, open-circuit voltage (V_oc_) and short-circuit current (I_sc_)	0.05–450 kPa pressure; up to 180% tensile strain	Max coupling factor: 1.05 × 10^–7^ TPa^−1^ Current density: 0.63 mA cm^−2^ Internal impedance: 180 Ω	[52]

## Wireless Data and Power Transmission

### Wireless Data Communication

The key characteristics of wireless transmission links, including data rate, stability, penetration depth, and energy efficiency, intrinsically govern the practical utility and operational longevity of flexible sensors. Among common transmission modalities, near-field magnetic coupling features low-power consumption and high efficiency for epidermal sensing applications, yet is restricted by a short effective transmission range. In contrast, far-field communication protocols such as Wi-Fi support extended transmission ranges and high data throughput for complex signal transmission, but their high-power consumption generally limits their integration into miniaturized or battery-less flexible platforms. As an alternative approach, non-electromagnetic acoustic and ultrasonic transmission links enable stable and low-loss signal propagation in biological aqueous environments, which renders them particularly suitable for monitoring deep-tissue implanted devices.

#### Near-Field Wireless Transmission

Wireless near-field transmission relies on electromagnetic inductive coupling, enabling stable short-range data exchange and energy transfer under low-power conditions. Benefiting from low attenuation in biological tissues, high coupling efficiency and the elimination of bulky radio-frequency modules, this approach is particularly suitable for flexible and implantable physiological monitoring systems that demand high structural compliance [53, 54]. Within this regime, operation in the low-frequency and high-frequency bands facilitates centimeter-scale communication through magnetic field coupling, with near-field communication (NFC) and inductor–capacitor (LC) resonant coupling serving as two typical implementation pathways.

NFC typically operates at 13.56 MHz, leveraging magnetic field coupling between coils to achieve low-power data interaction alongside concurrent energy supply. This characteristic renders it well suited for the continuous acquisition of epidermal physiological signals. By contrast, LC resonant magnetic coupling regulates resonant frequency through inductance or capacitance variations induced by external mechanical, chemical, or electrical stimuli. This mechanism boasts robust anti-interference capability and high spectral resolution, thereby making it more appropriate for flexible wearable and implantable systems that demand high measurement precision, long-term stability, and energy self-sufficiency.

**Fig. 4:**
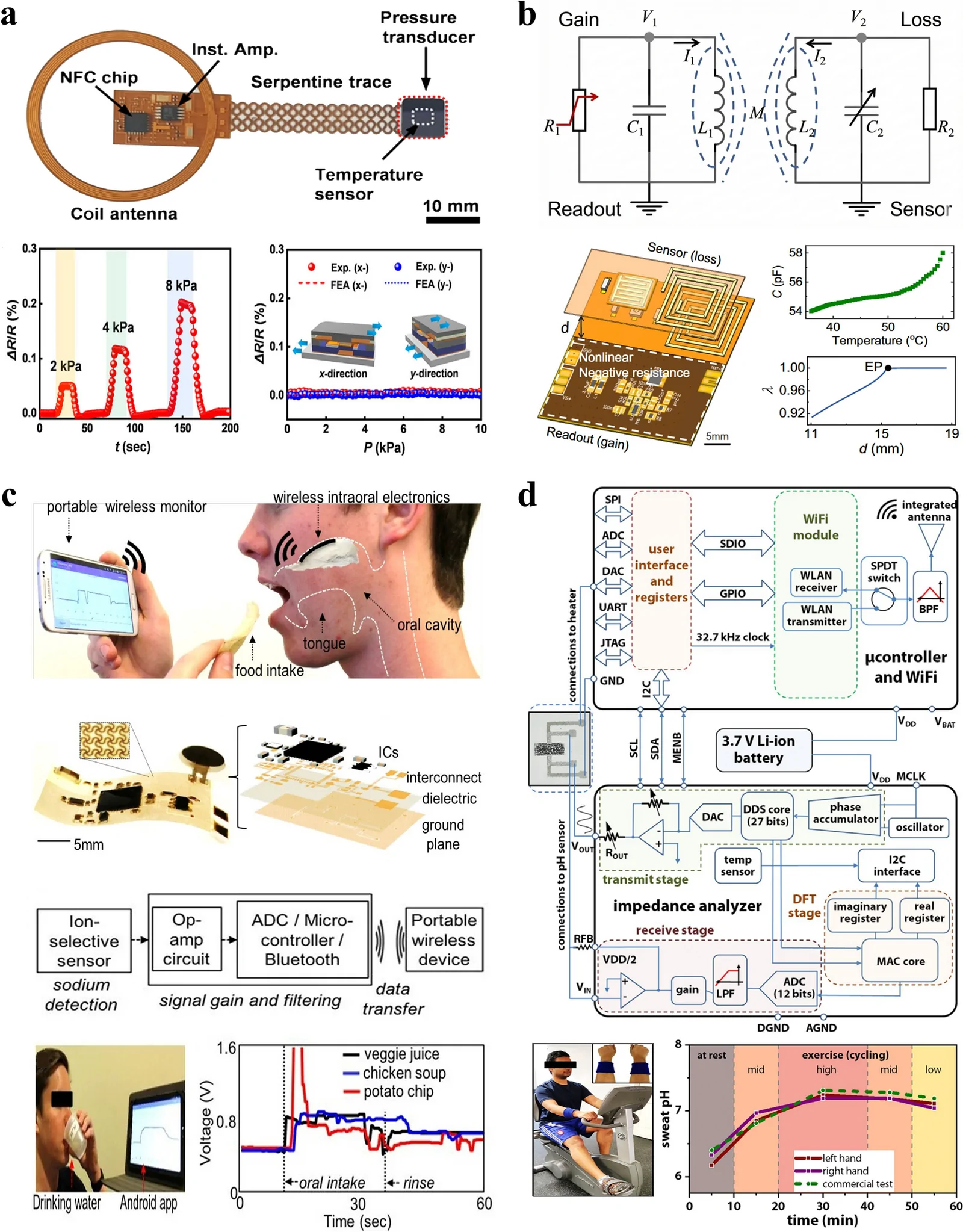
Representative implementations of different wireless transmission strategies in flexible and wearable health monitoring. **a** A battery-free flexible patch based on NFC, enabling multipoint real-time skin pressure and temperature readout via coil magnetic coupling [55]. Reproduced with permission. Copyright 2021, Nature Communications. **b** LC passive wireless sensors with inductive and capacitive designs, where resonant-frequency shifts induced by temperature changes allow high-resolution, power-free temperature detection [56]. Reproduced with permission. Copyright 2024, Nature Communications. **c** An intraoral flexible electronic system based on BLE, employing far-field wireless links to monitor sodium intake during eating, providing a portable quantitative tool for hypertension management [59]. Reproduced with permission. Copyright 2018, PNAS. **d** Flexible electronic stickers using Wi-Fi far-field communication, achieving medium- to long-range continuous monitoring of sweat pH through high-frequency RF links, suitable for real-time data uploading during physical activity [60]. Reproduced with permission. Copyright 2020, Biosensors and Bioelectronics

In the context of multi-parameter monitoring at the skin interface, Oh et al. [55] (Fig. 4a) developed a battery-free flexible NFC sensing system, which enables multipoint monitoring of skin pressure and temperature in bedridden patients. Operating under a constrained energy budget, the system maintains a stable communication distance and continuous readout capability; meanwhile, it incorporates temperature drift compensation to enhance the reliability of long-term data acquisition. For passive temperature sensing applications, Chen et al. [56] (Fig. 4b) proposed an LC wireless sensing architecture based on a nonlinear parity time symmetric structure, where inductively coupled coils facilitate dual channel transmission of energy and signals. This system exhibits a superlinear temperature response and retains high-frequency resolution as well as consistent readout performance under low-power operation. These two approaches, respectively, emphasize multi-parameter integration capability and temperature sensing resolution, reflecting distinct design trade-offs between functional prioritization and system load constraints. In contrast to the aforementioned passive and multi-parameter platforms, Zulqarnain et al. [57] developed a flexible wireless electrocardiogram (ECG) patch based on amorphous indium gallium zinc oxide (a-IGZO) thin-film transistors, which prioritizes the integrity of the biopotential signal acquisition chain. By integrating front-end amplification, signal conditioning and digitization modules, the system achieves reliable extraction of cardiac rhythm features; it also employs contactless NFC transmission for near-field data backhaul, rendering it suitable for low-power packaged systems dedicated to continuous ECG monitoring.

In short-range physiological monitoring, near-field magnetic coupling offers 20%–40% efficiency at 1–5 cm distances, making it ideal for low-power flexible implants. However, it faces several distinct limitations: low bandwidth (< 5 Mbps), eddy current losses that reduce the quality factor by 30%–60%, tissue-induced measurement drift, and a maximum effective range of 5–10 cm [58]. Furthermore, increasing coupling efficiency often requires larger coils, conflicting with the mechanical compliance of flexible devices. Future optimizations require adaptive resonant tuning and environmental compensation to maintain coupling stability. Simultaneously, the integration of signal compression and event-driven sampling can maximize the utilization of limited bandwidth, provided that persistent challenges including deformation-induced parameter drift, depth-efficiency trade-offs, and multi-node magnetic interference are effectively addressed.

#### Far-Field Wireless Data Communication

Wireless far-field transmission relies on electromagnetic radiation to achieve medium- to long-range data and energy transfer, where the transmitting antenna converts electrical signals into electromagnetic waves and the far-field receiving antenna reconverts them into electrical signals [61]. Its core performance is jointly determined by the transmission power, antenna gain, path loss, operating frequency, and modulation scheme, with the link margin directly affecting achievable communication distance, data rate, and stability. From a system architecture perspective, far-field approaches can be categorized into two technical routes: passive tag-based systems and active wireless systems.

The former, with ultrahigh-frequency radio-frequency identification (UHF RFID) as a typical example, relies on external carrier excitation and employs backscattering technology to achieve ultra-low-power identification and data feedback. In the context of flexible skin-interfaced electronics, these passive systems often utilize stretchable dipole or patch antennas printed on elastomeric substrates, enabling battery-free sensing of physiological parameters over several meters. Link design for such systems primarily focuses on reader equivalent isotropically radiated power (EIRP), tag backscatter coefficient, and loss control in the near-field-to-far-field transition region adjacent to the human body. Crucially, unlike the magnetic fields utilized in near-field coupling, far-field electromagnetic waves suffer from severe absorption and severe antenna detuning induced by the high dielectric constant of human tissues. Consequently, implementing far-field RFID on the human body often necessitates specialized antenna geometries and impedance-matching strategies to mitigate these biological tissue effects. The latter encompasses active wireless protocols including BLE, Wi-Fi, millimeter wave and ultra-wideband (UWB), characterized by controllable transmission power and independently modulated links. Operating under higher link budgets, these active systems achieve longer communication distances, higher bandwidth, and improved data rates, rendering them the primary solution for remote physiological monitoring and real-time data streaming applications.

Lee et al. [59] (Fig. 4c) developed a stretchable intraoral hybrid electronic system, which employs a BLE low-power link to establish stable communication over longer ranges. The system integrates a microstructured sodium ion selective sensor with a low noise amplifier, achieving a resolution of approximately 4.7 mV and an ion selectivity of 190 mV per decade while maintaining a data packet loss rate below a few percent over communication distances exceeding 10 m. In the context of far field monitoring of biofluid chemical signals, Pal et al. [60] (Fig. 4d) developed waterproof electronic stickers (WPEDs) that combine impedance spectroscopy with Wi-Fi far-field links to establish a complete measurement pathway, enabling stable uplink data transmission over several meters. This system achieves a pH resolution of approximately 0.1, with an instantaneous transmission power on the order of tens of milliwatts. In comparison with Wi-Fi-based impedance systems, the BLE architecture supports long-range periodic data transmission at lower power consumption, albeit with reduced throughput, making it more suitable for low sampling rate ion monitoring tasks. Zhang et al. [62] developed a waist worn system that integrates a triboelectric nanogenerator (TENG) as a self-powered sensing front end. The conditioned signals drive a Bluetooth link, enabling real-time discrimination of thoracic and abdominal breathing patterns. The system can continuously transmit respiratory waveforms over a 10-m range, with typical energy output sufficient to support milliwatt scale signal chains. It also employs respiratory frequency feature extraction to detect abnormalities associated with obstructive sleep apnea syndrome (OSAS).

Far-field electromagnetic communication supports long-range, high-throughput multi-node sensing, but faces severe engineering challenges in wearable applications. Mechanical deformation and high dielectric loading from the human body cause resonance frequency shifts, impedance mismatch, and radiation distortion, considerably reducing the effective link budget. Furthermore, sweat accumulation alters the local dielectric environment [63], while the inherently low conductivity of stretchable conductors degrades the antenna quality factor and bandwidth [64, 65]. Overcoming these bottlenecks requires integrating advanced conductive materials (e.g., nanowire networks, liquid metals) and multiscale structured antennas to maintain radiation consistency. Additionally, utilizing dynamic body loading models, adaptive impedance matching, and emerging high-bandwidth platforms (5G, Wi-Fi 6E, mmWave) can stabilize link margins. A coordinated design framework spanning materials, antenna architecture, and adaptive protocols is essential to realize efficient, low-power remote physiological monitoring.

#### Non-Electromagnetic Communication

Wireless non-electromagnetic communication utilizes acoustic, ultrasonic, or mechanical waves to overcome the severe attenuation of electromagnetic signals in highly conductive biological media. By maintaining low-loss propagation despite strong absorption, high impedance mismatches, or even metallic shielding, these modalities ensure reliable data and energy transfer. Consequently, wireless non-electromagnetic communication serves as a critical complement to electromagnetic systems, providing superior tissue penetration and cross-medium reliability for challenging applications like deep-seated implants, biofluid channels, and tissue-encased structures.

**Fig. 5:**
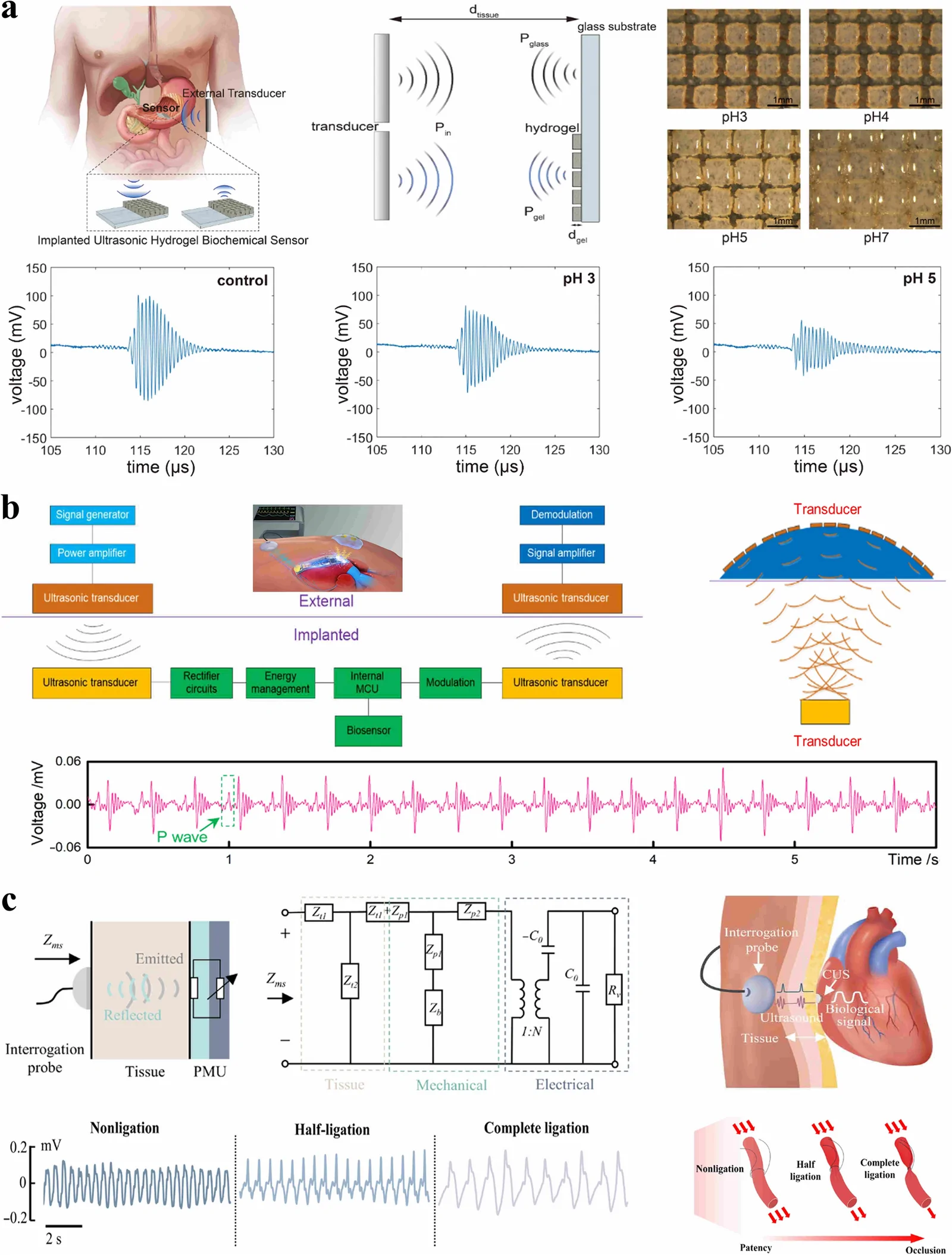
**a** Schematic illustration and experimental demonstration of an implanted ultrasonic hydrogel biochemical sensor for wireless pH monitoring, where pH-responsive hydrogel volume changes are detected via ultrasonic pulse-echo readings [66]. Reproduced with permission. Copyright 2020, Frontiers in Bioengineering and Biotechnology. **b** System architecture showing bidirectional data communication and non-electromagnetic acoustic power transfer for a flexible cardiac pacemaker, accompanied by the acquired electrocardiogram signal highlighting the P wave [67]. Reproduced with permission. Copyright 2021, Science Advances. **c** Wireless sensing and communication realized via an interrogation probe and an equivalent electromechanical circuit model, demonstrating the monitoring of blood vessel occlusion status through ultrasonic pulse modulation [68]. Reproduced with permission. Copyright 2025, Science Advances

In the context of wireless detection of biofluid chemical signals, Nam et al. [66] (Fig. 5a) proposed a hydrogel-based ultrasonic backscatter scheme. Through the adoption of pulse excitation and differential echo reading, this system enables passive wireless querying. The hydrogel forms tunable scattering layers at two interfaces; the quantity being sensed is entirely determined by the difference in scattering intensity induced by volumetric changes of the hydrogel, without the requirement for a local power source or active electronic components. Operating within the 5–10 MHz ultrasonic range, the prototype achieves a query depth of approximately 10 cm and a pH resolution of 0.2 in aqueous environments, highlighting a design approach for passive chemical readout based on the volumetric response of materials. For implantable wireless physiological monitoring, Jin et al. [67] (Fig. 5b) developed a thin and flexible system that realizes bidirectional communication through acoustic power transfer. By integrating an on-chip rectifier, microcontroller, ultrasonic transducer, and fractal serpentine interconnects, the system combines active data links with acoustic energy delivery. Operating in the 1–3 MHz acoustic band, it maintains a stable power link within several centimeters of soft tissue and provides a communication range of approximately 5 cm, supporting applications that require active modulation and protocol control, such as cardiac rhythm monitoring and electrical stimulation. Besides, Wang et al. [68] (Fig. 5c) proposed a passive modulated ultrasonic system. By directly modulating the amplitude or phase of ultrasonic pulses, physiological signals such as intrathoracic pressure are encoded onto the reflected acoustic carrier, enabling circuit free implantation and passive sensing. Operating at approximately 3–5 MHz, the system achieves effective monitoring depths of several centimeters, eliminating the need for rectification and amplification circuits while preserving high-link readability. Its depth coverage is considerably greater than that of NFC, making it suitable for extension to deep tissue respiration and blood pressure monitoring applications. Recently, Liu et al. [69] reported an embedded ultrasonic soft sensor integrated with magnetic actuators for wireless robotic sensing. Remarkably, the integrated flexible device features ultra-compact dimensions of merely 1.3 × 1.3 × 1.6 mm^3^, tissue-level softness with a modulus of 98 kPa, and a lightweight design of 4.6 mg. Driven by external magnetic fields and passive ultrasound communication, this millimeter-scale system enables precise, in situ wireless detection of biophysical parameters, including deep-tissue force and viscosity.

Wireless non-electromagnetic communication is capable of maintaining low loss propagation in human tissues characterized by high attenuation and strong absorption. Wireless acoustic communication excels in deeply implanted devices (e.g., thoracic, abdominal, and perivascular regions) due to superior impedance matching and low-loss propagation in aqueous tissues. However, practical performance is hindered by tissue-induced scattering, diminished acoustic pressure from miniaturized transducers, and severe multipath interference caused by complex in vivo geometries [70]. Overcoming these challenges requires advanced piezoelectric materials with high electromechanical coupling, directional ultrasonic microarrays to stabilize beams, and adaptive demodulation algorithms to ensure data integrity. Furthermore, integrating acoustic links with electromagnetic or mechanical modalities could enable more robust, low-power continuous physiological monitoring in heterogeneous environments.

Table 2 summarizes representative recent studies of polymer-based flexible wireless sensors employing different signal transmission modalities. The table systematically compares their typical transmission strategy, typical method, transmission parameters, and specific sensing modality, providing a reference for the design and optimization of wireless transmission in flexible sensor systems.

**Table 2:** Summary of polymer-based flexible wireless sensors employing different signal transmission modalities

Transmission Strategy	Typical Method	Transmission Parameters	Specific Sensing Modality	Refs
Near-Field Communication	NFC	Operating Frequency: 13.56 MHz Transmission Distance: Up to 10 mm Tissue Penetration: Epidermal (skin surface)	Flexible electrochemical immunosensor (Differential Pulse Voltammetry, DPV)	[71]
	NFC	Tissue Penetration: Epidermal/Skin surface Power Consumption: Zero-power (self-powered RFID) or low-power (Bluetooth)	Flexible graphene-knitted temperature sensor (resistance decreases as temperature increases)	[72]
	Wireless LC circuit	Operating Frequency: 550–600 MHz Transmission Distance: 2–7 mm Tissue Penetration: ~ 1.5 mm (skin) to subcutaneous	Capacitive pressure sensing	[73]
Far-Field Communication	Wireless Bluetooth (via Arduino and HC-05 module)	Operating Frequency: Standard Bluetooth Transmission Distance: Standard Bluetooth range (to smartphone) Tissue Penetration: External wearable	Chemiresistive sensing (Resistance modulation for humidity, breath, ethanol, and temperature)	[74]
	Wireless transmission via ESP32 microcontroller (Bluetooth/Wi-Fi)	Operating Frequency: Standard Bluetooth/Wi-Fi bands (Target physiological signals: 0.5–2 Hz) Transmission Distance: Standard wireless range to smartphone or PC Tissue Penetration: Non-invasive surface attachment; propagates acoustic waves via an air channel	Mechano-acoustic piezoresistive sensing	[75]
	Printed flexible antennas combined with rigid RFICs	Frequency: 902–928 MHz (UHF-RFID) Bandwidth: 22–24 MHz Distance: 40–90 cm Power: Passive (battery-less; harvests RF energy)	Temperature Sensing: Uses the RFIC’s on-board sensor Capacitive Touch Sensing: Uses printed interdigitated electrodes to detect user interaction	[76]
	Far-Field LoRa	Operating Frequency: 433/868 MHz (LoRa ISM bands) Tissue Penetration (SAR): 1 g average SAR values are 1.25 W/kg (433 MHz) and 0.89 W/kg (868 MHz)	Remote health monitoring for elderly care and ward-to-server relay	[77]
	Far-Field RF Backscatter	Operating Frequency: 3.5 GHz (backscatter), 2.4 GHz (RF EH) Bandwidth/Data Rate: 500 kHz subcarrier; 125–500 kbps Transmission Distance: 25 cm (at 500 kbps) up to 250 cm (at 125 kbps)	Imaging of wounds or oral/pharyngeal lesions in outpatient or home settings	[78]
Non-Electromagnetic Communication	Acoustic/Ultrasound Communication	Operating Frequency: 0.2–10 MHz (Communication); 20–200 kHz (Energy Harvesting) Transmission Distance: Up to 4 cm (underwater) Tissue Penetration: 10 mm (tested in porcine tissue)	Real-time monitoring of physiological parameters via changes in ultrasound velocity	[79]
	Magnetic Backscatter	Operating Frequency: ~ 5.95–5.96 MHz (with 262 kHz modulation) Data Rate: Up to 5 kbps Transmission Distance: Up to 25 cm Tissue Penetration: Deep tissue (tested in porcine ribs)	Fingerprint-free, mm-level in-body localization	[80]
	Ultrasonic Networking	Operating Frequency: 17–22 kHz Data Rate: Up to 2.76 kbps Tissue Penetration: High (lower attenuation than RF signals) Power Consumption: Tx power of 20 mW (13 dBm)	Uses commercial off-the-shelf (COTS) microphones and speakers as ultrasonic transducers	[81]

### Wireless Power Supply and Energy Management

Continuous health monitoring employing flexible sensors demands alternatives to conventional miniature batteries, which suffer from inherent limitations including bulky size, mechanical rigidity, and short operational lifespans. To tackle this issue, three primary power supply strategies have been developed. First, near-field power transfer realized through magnetic or capacitive coupling exhibits high energy efficiency for skin-mounted or shallowly implanted devices, yet is confined to short transmission distances and necessitates precise positional alignment. Second, far-field power transfer schemes such as radio frequency (RF) or microwave approaches enable longer-distance power delivery for large-area wearable systems with milliwatt-level output power, but are compromised by low energy density and significant energy attenuation caused by tissue absorption. Lastly, self-powered strategies can scavenge mechanical, thermal, or chemical energy by means of triboelectric or piezoelectric generators, thereby supplying auxiliary power ranging from microwatts to milliwatts for low-power sensor nodes.

#### Near-Field Power Transfer

Wireless near-field power prioritizes efficient, stable energy transfer over data exchange using either magnetic (inductive) or electric (capacitive) coupling. Inductive coupling delivers high-efficiency, battery-free power via alternating magnetic fields, though its range is limited by coil size, alignment, and metal interference [82]. In contrast, capacitive coupling utilizes alternating electric fields, offering lightweight and stretchable designs ideal for flexible wearables. However, it has a shorter transfer distance and its efficiency is highly sensitive to electrode geometry, gaps, and surrounding dielectrics, including variations in human tissue. Ultimately, optimizing either method enables safe and reliable energy links for short-distance flexible sensors.

**Fig. 6:**
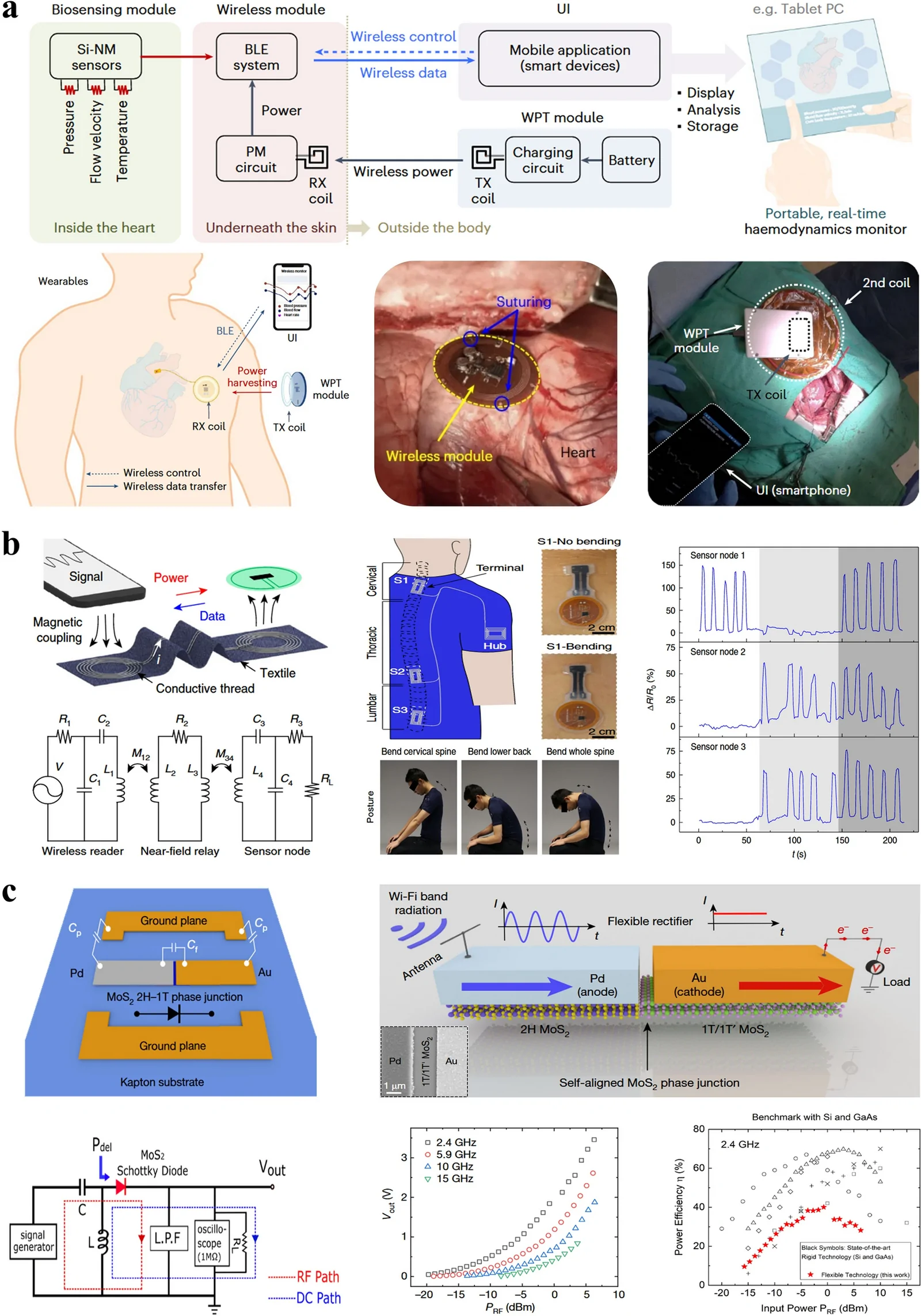
**a** An implantable wireless system for real-time hemodynamics monitoring. The system enables energy harvesting via subcutaneous receiving coils for WPT and utilizes a BLE module for data transmission to a user interface [83]. Reproduced with permission. Copyright 2023, Nature Biomedical Engineering. **b** Battery-free body-area sensor network utilizing magnetic coupling. It achieves multi-node wireless powering and data transmission through fabric-integrated near-field relay nodes, demonstrated here for continuous spinal posture monitoring [84]. Reproduced with permission. Copyright 2020, Nature Communications. **c** Flexible rectenna based on a two-dimensional MoS_2_ semiconductor–metal phase junction, exhibiting high RF-DC conversion efficiency. It enables efficient far-field energy harvesting in the Wi-Fi band, outputting approximately 3.5 V DC under 5-mW excitation [86]. Reproduced with permission. Copyright 2019, Nature

Kwon et al. [83] (Fig. 6a) reported a battery-free, wirelessly powered implantable platform that enables continuous and real-time measurement of vascular pressure, flow velocity, and temperature. The system adopts a near-field communication-based wireless power transfer (WPT) scheme, wherein a subcutaneous receiving coil combined with a matching capacitor forms a resonant cavity, which efficiently captures energy from an external transmitting magnetic field. To address common challenges in the implant environment, such as coil angular misalignment, positional drift, and load fluctuations caused by hemodynamic changes, the design integrates rectifiers, charge pumps, and supercapacitors into a multistage energy management chain. This configuration provides transient energy storage and voltage stabilization at the receiver, ensuring stable power delivery even under conditions of reduced coupling efficiency or increased sensor module power consumption. By contrast, Lin et al. [84] (Fig. 6b) proposed a fabric-based near-field power supply system for multi-node body area networks. Its core innovation lies in the use of conductive embroidery threads in textiles to form planar inductive arrays serving as energy relay structures, which extends the coverage beyond traditional point-to-point coil transfer. Driven by a time-varying magnetic field, the fabric-based relay generates mobile magnetic hotspots within a range of approximately 1 m. When a readout device approaches the relay region, efficient energy coupling is achieved at the skin-mounted nodes, with a power transfer efficiency exceeding 10% and an effective supply distance of less than 1.5 cm. The relay structure considerably reduces the nodes’ dependence on the transmitter position, enabling continuous power delivery and communication across multiple sensor nodes located on different parts of the body. In minimally invasive therapeutic applications, Lee et al. [85] developed a flexible, adhesive, and biodegradable wireless implant device. Powered by a near-field alternating RF magnetic field (220 kHz), the device drives a magnesium-based heater for localized hyperthermia therapy. Under typical coupling conditions, the heating unit can deliver tens to hundreds of milliwatts of thermal power, maintaining the treatment region at approximately 42–45 °C for several minutes to tens of minutes, and enabling controlled drug release.

Wireless near-field power eliminates the issues of bulk, rigidity, and surgical replacement risks associated with traditional batteries, enabling safer and battery-free operation for implantable devices. While low-frequency NFC provides effective deep-tissue penetration, it encounters constraints including magnetic coupling instability caused by misalignment and limited transmission range, as well as material degradation manifested in coil fatigue and corrosion. To address these challenges, future research and development efforts concentrate on three key directions: Employing advanced materials such as liquid metals and biodegradable conductors to enhance structural durability, incorporating smart circuit modules including adaptive power regulation to stabilize energy transmission, and designing optimized system architectures represented by relay networks to improve overall system coverage and operational robustness.

#### Far-Field Power Transfer

Wireless far-field power transfer transmits energy over medium-to-long distances via RF electromagnetic waves, and the receiver converts the signal into direct current (DC) power using a rectenna to enable the continuous operation of battery-free or low-power devices [87]. Crucially, the overall effectiveness of this process is governed by two key metrics: the transmission-reception (Tx-Rx) efficiency and the RF-DC conversion efficiency. Tx-Rx efficiency represents the ratio of power captured by the receiving antenna to the total radiated power, while RF-DC efficiency dictates how effectively the rectenna converts this captured energy into usable DC power. Compared with near-field methods, far-field power supply overcomes distance limitations, offering wide coverage and flexible deployment, making it suitable for distributed monitoring, multi-node wearable networks, and large-area health monitoring. Nevertheless, practical applications still face two major constraints. First, regulatory limits restrict transmit power to the milliwatt level, resulting in a rapid decline in available power density and consequently low Tx-Rx efficiency over increasing distances. Second, human tissues absorb and scatter high-frequency electromagnetic waves; this not only further degrades Tx-Rx coupling but also necessitates comprehensive safety evaluations.

Zhang et al. [86] (Fig. 6c) developed an atomic scale flexible rectenna based on a MoS_2_ metal semiconductor heterostructure, enabling efficient energy harvesting from the 2.4 GHz to 5.9 GHz Wi-Fi bands. The device adopts a self-aligned lateral Schottky architecture and achieves a RF-DC conversion efficiency of 40.1% at a 10-GHz cutoff frequency. Under only 5-mW RF excitation, it can output 3.5 V DC, exhibiting considerably higher performance than conventional flexible rectifiers. Its operating range extends to the X band, supporting non-line of sight and meter level stable power delivery, which provides a high-performance technical pathway for multi-band far-field energy transfer. While this work highlights significant advancements in RF-DC conversion, a comprehensive understanding of far-field systems necessitates evaluating the Tx-Rx efficiency as well. Even with an optimal RF-DC rectifier, the final power delivered is bottlenecked by the Tx-Rx efficiency, which is heavily dependent on antenna design, spatial alignment, and environmental attenuation. It should be emphasized that far-field power transfer is strictly constrained not only by practical energy budgets but also by complex environmental influences. Available ambient irradiance typically decreases with the square of the distance, and RF transmit power is restricted by human safety limits. In comparison, Zhao et al. [87] constructed a broadband monopole antenna using flexible MXene. Through interface engineering, the antenna achieves a 75% relative bandwidth and a 68%–76% radiation efficiency over the 1.7–4.0 GHz range, demonstrating efficient capture of multi-band far-field RF energy. It maintains stable energy reception under non-line-of-sight conditions and at distances of 1–5 m, validating its potential for continuous and reliable far-field power supply in wearable and Internet-of-Things devices. It should be noted that the actual usable power is still constrained by environmental irradiance and human safety limits. Thus, high-efficiency energy harvesting primarily maximizes the utilization of RF energy within a limited power budget, rather than providing unlimited output.

Far-field wireless power transfer enables meter-level, non-contact energy delivery for distributed wearable networks, overcoming the spatial constraints of near-field methods. While integrating these systems into flexible substrates enhances patient comfort, practical implementation faces significant challenges: severe RF signal attenuation in air and biological tissues, the limited mechanical resilience of rectennas under repetitive bending, and underdeveloped multi-node power management architectures. To address these issues, future research should focus on multi-band energy harvesting to boost overall power, adaptive impedance matching to mitigate environmental fluctuations, and the use of metasurfaces and beamforming to improve transmission directivity and coupling efficiency.

#### Energy Harvesting

To overcome the endurance, volume, and maintenance limitations of conventional batteries, energy harvesting has emerged as a crucial strategy for the long-term, continuous operation of flexible electronics. In human-centric applications, ambient energy is primarily harvested through piezoelectric, triboelectric, thermoelectric, and photovoltaic mechanisms. Although the harvested energy is often characterized by low-power density and strong variability, optimizing these harvesting technologies is essential to eliminate external power dependencies and facilitate the practical implementation of self-powered wearables in health monitoring.

**Fig. 7:**
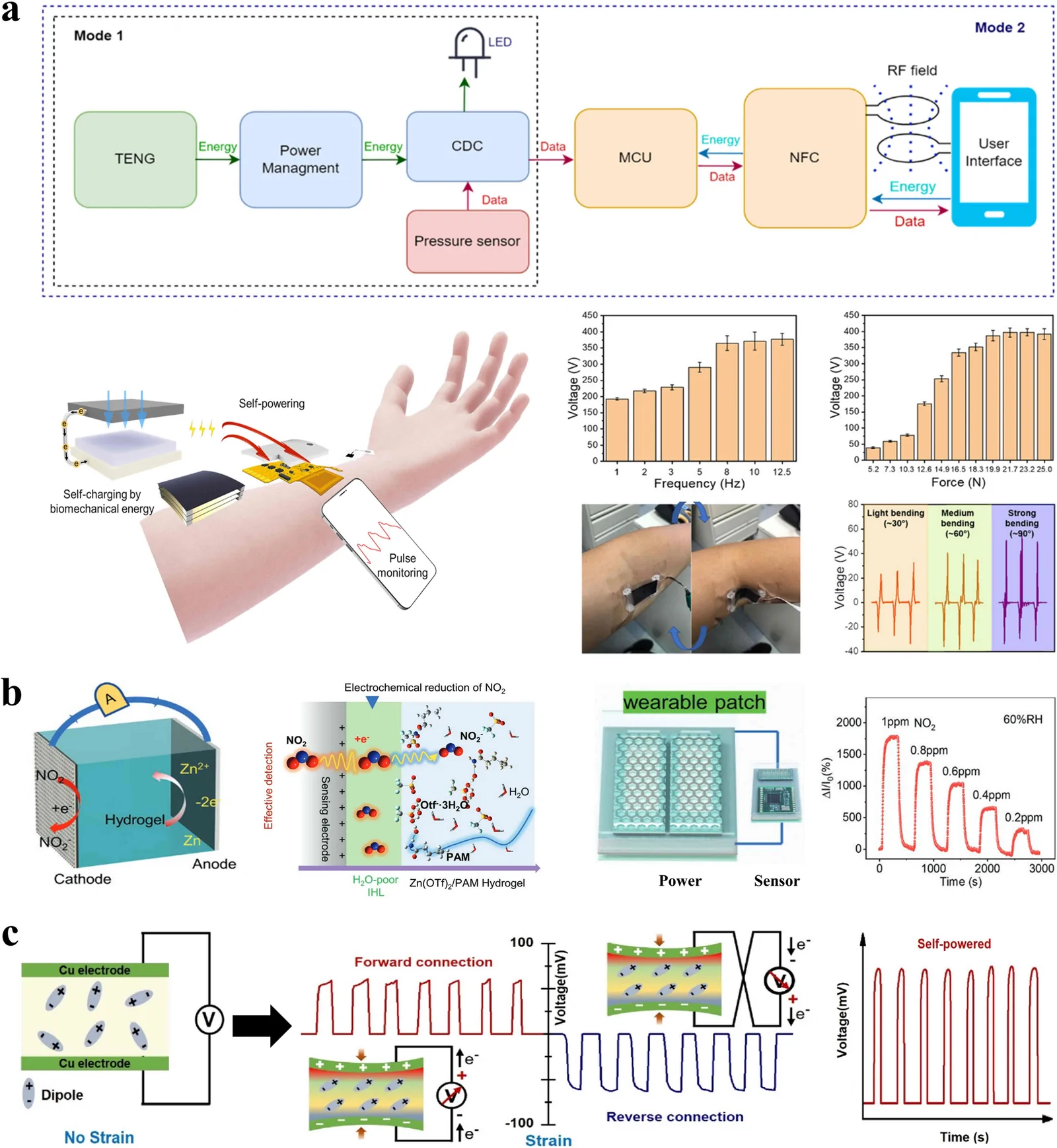
**a** Self-powered system based on a MXene triboelectric nanogenerator, harvesting energy from human mechanical motion for real-time monitoring of radial artery pulse waveforms [88]. Reproduced with permission. Copyright 2022, Nano Energy. **b** Self-powered NO_2_ sensor constructed from hydrogel materials, enabling gas detection without an external power source [89]. Reproduced with permission. Copyright 2023, Advanced Functional Materials. **c** Self-powered wireless monitoring system based on piezoelectric hydrogel, used for acquisition and transmission of motion signals [90]. Reproduced with permission. Copyright 2023, Nano Energy

Yi et al. [88] (Fig. 7a) demonstrated a sophisticated closed-loop wearable framework centered on a MXene triboelectric nanogenerator. This system effectively transduces mechanical energy derived from routine human kinetics into a stable electrical reservoir, providing peak power levels that satisfy the collective requirements of high sensitivity pressure transducers and near-field communication modules. This configuration establishes a seamless operational cycle involving kinetic energy acquisition, triboelectric conversion, and wireless data dissemination, thereby validating the practical utility of self-sustaining wearable electronics. A distinct approach was pursued by Wu et al. [89] (Fig. 7b), who realized a rechargeable nitric oxide sensor designed for autonomous chemical detection utilizing a flexible solid-state electrolyte. The rechargeable nature of this device stems from its capacity to restore its operational potential via spontaneous environmental electrochemical reactions, bypassing the external bias typically necessitated by traditional electrochemical gas sensing platforms. By leveraging interfacial charge modulation, the sensor achieves high flexibility and reusability, and when coupled with low-power circuitry, it facilitates robust wireless gas surveillance and early warning capabilities. Furthermore, Fu et al. [90] (Fig. 7c) introduced a mechanically driven self-powered system utilizing piezoelectric hydrogels. These materials are characterized by exceptional compliance, efficient mechanical-to-electrical energy transduction, and superior biocompatibility, enabling the generation of significant charge even under minimal structural deformation. The energy harvested through this mechanism is sufficient to sustain the synchronized operation of multi-channel sensor arrays and wireless communication units, allowing for the precise and simultaneous monitoring of complex joint kinematics and multi-regional bodily movement.

Self-powered strategies, such as triboelectric and piezoelectric harvesting, enable battery-independent operation for polymer-based flexible wireless sensors by converting low-frequency human motion into electrical power. While advantageous for their architectural simplicity, these systems face notable limitations. Triboelectric generators are highly sensitive to ambient humidity and sweat, which degrade their interfaces and reduce output, though recent sweat-resistant piezoelectric designs offer promising alternatives [91]. Meanwhile, piezoelectric materials are constrained by low volumetric energy density, struggling to support high-demand tasks like continuous communication. Furthermore, poor impedance matching between harvesting, rectification, and storage components often limits the actual power available. To achieve truly autonomous monitoring, future research must focus on multi-mechanism coupling (e.g., synergizing triboelectric, piezoelectric, and thermal harvesting) to capture broader motion frequencies, optimizing rectification and storage efficiencies, and implementing low-power communication and algorithmic strategies including event-triggered sampling and adaptive data compression to minimize overall systemic consumption.

Table 3 provides a systematic comparison of recent polymer-based flexible wireless sensors employing different power supply methods and energy management strategies, covering energy supply modes, key features, electrical parameters, and achievable power output/power density. This comparison not only highlights the advantages and limitations of current self-powered strategies but also offers targeted guidance for future optimization in terms of energy harvesting performance, system efficiency, and alignment with application scenarios.

**Table 3:** Summary of power supply and energy management strategies for polymer-based flexible wireless sensors

Energy strategy	Energy supply mode	Key features	Electrical parameters	Achievable power output/Power density	Advantages and Limitations	Refs
Near-field	WPT with real-time automated impedance matching	2D open-circuited spiral antenna; purely electronic automated impedance matching	13.56 MHz (ISM band); Q-value: 460; Inductance: 7100 nH; Capacitance: 18.4 pF	Maximum 92% power transfer efficiency	Advantages: Safe contactless charging, adaptive to air gaps Limitations: Efficiency sensitive to resonator coupling	[92]
	Passive RF powering via a glasses-integrated reader	Passive LCR resonator; chip-free and battery-free; remote impedance matching via IMTR	~ 99.0 MHz resonant frequency; 0.43 µH inductance; 3.03 pF capacitance; 41 Ω resistance	No direct power output; maintains a 4 dB signal amplitude at 14 mm	Advantages: Battery-free eye safety; 14 mm reading distance Limitations: Signal relies on distance and coupling	[93]
	Magnetic/inductive coupling; RF energy harvesting	Resonant or non-resonant operation Series or parallel matching networks Simultaneous power and data transfer via load modulation	Frequencies: < 100 kHz, 87–205 kHz, 6.78 MHz, 13.56 MHz Focuses on loosely coupled systems (k < 0.5)	Evaluated via relative metrics (PTE and TP) rather than absolute wattage Highly dependent on the precise matching of the coupling coefficient (k) and load resistance	Advantages: Versatile batteryless operation Limitations: Requires precise impedance matching and coil alignment	[94]
	Resonant coupling between large on-body primary coils and in-body receivers	Helmholtz-based large primary coil for uniform internal magnetic fields Series–Parallel (SP) compensation for optimal power transfer	Frequency range: 10 kHz–10 MHz Optimal frequency: ~ 4 MHz Load resistance range: 0–1000 Ω	Target power: 10 mW for leadless pacemakers Max output: Up to 310 mW for titanium-housed devices	Advantages: Deep tissue penetration, low heating, misalignment tolerance Limitations: Titanium eddy currents reduce coupling; ferrite limits MRI	[95]
Far-field	Converting ambient RF energy into DC power for wearable devices	Unidirectional dual-band magnetoelectric dipole Direct conjugate matching (no extra matching circuit)	Operating bands: 3.5 GHz and 4.9 GHz Peak efficiency (at 4 dBm): 53.43% Low-power efficiency: > 10% at -15 dBm	Ambient RF density: ~ 1.87 μW/m^2^ Indoor dynamic output: 53.2 mV average, 65.9 mV peak	Advantages: High front-to-back ratio, excellent body isolation Limitations: Radiation drops at high bending curvatures (< 20 mm)	[96]
	Harvesting ambient Wi-Fi RF (2.4–2.6 GHz) to power sensors and BLE	Ultra-thin polyimide (PI) substrate (50 µm) Fully integrated flexible patch	Frequency: 2.4–2.6 GHz Antenna gain: 5.6 dBi Output voltage: Stable 3.3 V DC System power: 4.09 mW (at 100 Hz sampling)	Charging speed: 220 µF to 4.12 V in 23.24 s (at 100 cm, 0.8 dBm input) Max output: 0.0415 mW (at 2 kΩ load, 0 dBm input)	Advantages: Continuous ambient Wi-Fi energy harvesting Limitations: Efficiency depends on source distance and angle	[97]
	Dual-band RF harvesting, DC rectification, supercapacitor storage	Dual-source harvesting, battery-less micro-pump actuation, low-power long-range backscatter	Frequencies: 850–950 MHz (RFID) and 464.5 MHz (two-way talk radio) Communication power consumption: < 600 µW Reading range: 3 m	Near-field charging: 4.63 s (at 464.5 MHz, 20 dBm input) Far-field charging: 6.6 min (at 900 MHz, 0 dBm input)	Advantages: Autonomous, flexible, low-cost fluid sensing Limitations: Fast detection needs near-field sources due to slow far-field charging	[98]
	Wireless RF-DC power conversion	Compact binary fractal tree geometry Omnidirectional, dual-linear polarization	Bandwidth: 2.0–2.92 GHz (covers 5G n40/n7, ISM 2.45 GHz) Max radiation efficiency: 96.7% at 2.45 GHz Peak RF-DC efficiency: 45.49%	Peak: 4000 μW at 100 V/m Typical: ~ 100 μW at 30 V/m	Advantages: Weather-independent, sustainable wireless power Limitations: Performance drops with low input power, long distance, or low traffic	[99]
	Wireless RF-DC conversion with PMU storage	Circular polarization (CP) Low specific absorption rate (SAR)	Frequency: 2.45 GHz Max efficiency: 78.2% (at -5 dBm, 2 KΩ) Peak gain: 3.2 dBi	Indoor voltage: 0.98 V Outdoor voltage: 2.46 V PMU boosted voltage: 3.3 V	Advantages: High efficiency at low power, stable PMU charging Limitations: Resonant frequency shifts on wrist due to tissue impedance	[100]
Energy Harvesting	Single-electrode TENG; triboelectrification and electrostatic induction	Coaxial core–shell yarn; spring core with mechanoluminescent ZnS:Cu/PDMS shell	V_OC_: Up to 27 V I_SC_: Up to 0.12 µA Q_SC_: Up to 9 nC Contact Area: 0.85 cm^2^ Frequency: Stable outputs across 1–5 Hz	Driving Capability: A 40 × 40 mm^2^ fabric lights 60 series LEDs and powers a smartwatch Sensitivity: Ranges from 1.02 to 0.15 V N^−1^	Advantages: Breathable, reusable, simultaneous electrical/optical sensing Limitations: Coating thickness trade-off (strength/output vs. leakage)	[101]
	Fully self-powered via structural deformation and contact-separation cycles	Synergistic dual-effect energy generation; optimal performance at 20 wt.% BTO and 7 µm thickness	Peak voltage: 20.51 V (at 20 N); Peak current: 0.86 µA	Maximum power density: 130.12 mW m^−2^ (at 25 MΩ load)	Advantages: Stable hybrid energy output, highly durable (> 5000 cycles) Limitations: Excessive BTO doping (25 wt.%) causes agglomeration and weak output	[102]
	Continuous self-powered supply; boosted energy transfer	Integrated CNT/Cu heat conduction unit (HCU); Cu heat sink and polyimide side insulation	175–180 mV open-circuit voltage at a 9 K thermal gradient; 2.3 mW peak output power at a 10 K gradient	Power density of 69 μW cm^−2^ at a 9 K thermal gradient	Advantages: > 7 days continuous power, excellent skin conformality Limitations: Relies on fluctuating temp differences; low efficiency (1.9%-4.0%)	[103]
	Fully self-powered; real-time thermal voltage for wireless ADC/Bluetooth	Embedded static air pockets (< 0.05 W/mK); decoupling of thermoelectric and piezoresistive effects	Temperature resolution: 0.02 K Response time: 20 ms (strain)/240 ms (temperature)	Stable output voltage of 5.44 mV at an ~ 8 K thermal gradient (wrist-worn); peak power output achieved at a matching 110 Ω load resistance	Advantages: Exceptional elasticity, washable, stable under 50% strain Limitations: Difficult to integrate impregnation into large-scale knitting lines	[104]

### Wireless Data Processing Techniques

Processing complex and noisy physiological signals from polymer-based flexible wireless sensors requires balancing low-power consumption and minimal latency with high accuracy. To achieve this, this section explores system-level strategies including lightweight local preprocessing, direct transmission, conventional algorithms, and machine learning techniques.

#### Lightweight Local Preprocessing and Direct Transmission

Lightweight local preprocessing and direct transmission are specifically adopted to resolve the fundamental conflict between the continuous generation of raw physiological data and the strict energy budgets of wearable nodes [105]. To mitigate high-frequency environmental noise and powerline interference prior to transmission, the analog signals undergo low-pass or band-pass filtering immediately following digitization. Furthermore, to alleviate the bandwidth bottlenecks inherent in wireless communication, subsequent feature extraction and data compression steps are employed to strip away data redundancy, thereby considerably lowering transmission power consumption. Simplified modulation schemes are then adopted for wireless transmission, enabling low-power and real-time monitoring of physiological signals such as heart rate, respiration and motion.

In the wireless detection of biofluids and respiratory signals, where the primary challenges are weak signal amplitudes and severe power constraints, Zhang et al. [106] developed a battery-free wearable potassium ion (K^+^) sweat sensor patch, achieving high-precision and low-power detection through the optimization of the analog front-end. Specifically, to address the inherently weak amplitude of physiological electrochemical signals, programmable gain amplification was utilized to map 100–400 mV signals into the optimal linear range of the analog-to-digital converter (ADC). Concurrently, to filter out high-frequency quantization noise without imposing a heavy computational burden on the battery-less system, a cascaded integrator-comb filter was integrated alongside a 14-bit sigma-delta (Σ-Δ) ADC. Subsequently, digital signals were wirelessly read using the ISO15693 protocol, enabling sub-millimolar real-time monitoring without relying on an external power source.

A simplified architecture that limits sensing nodes to basic sampling and transmission considerably reduces circuit complexity and device heating, enhancing wearability and long-term stability. However, the absence of local preprocessing leaves raw signals highly vulnerable to noise and interference, which degrades data quality and increases the wireless transmission burden. To address this, future research should focus on the synergistic design of advanced sensing materials (e.g., nanowire networks, MXenes, ionogels) and optimized front-end circuitry (e.g., low-noise amplification, tunable filtering) to enable wideband, low-distortion, and stable signal acquisition directly at the source.

#### Conventional Algorithm-Based Data Processing

Conventional algorithm-based data processing techniques applied in polymer-based flexible wireless sensors are mainly dedicated to optimizing raw physiological signals by addressing specific signal distortions and data transmission bottlenecks. Through sequential implementation of filtering, feature extraction, and dimensionality reduction operations, high-dimensional and noise-contaminated data can be converted into low-dimensional and reliable information [107, 108]. Depending on the targeted signal processing issues, common conventional data processing methods can be classified into three categories. The first category includes time‐frequency analysis and filtering techniques, such as wavelet transform and Kalman filtering. These techniques are specifically designed to tackle non-stationary interference, such as sudden motion artifacts and electrochemical noise, making them well-suited for processing continuous physiological signals (e.g., heart rate, blood oxygen and respiration). The second category comprises frequency-domain and spectral analysis methods; Fourier transform, as a typical example, is used to address the challenge of extracting periodic or rhythmic features from complex mixed signals, which supports the stable identification of step frequency, respiratory rate, and motion cycles. The third category encompasses data compression and sparse representation approaches, with compressed sensing being a representative one. These methods directly resolve the bottleneck of high wireless data bandwidth by reducing data dimensionality while preserving critical information, thereby facilitating the extraction of motion states or ECG features in low-cost transmission scenarios. Due to their relatively simple computational structures, these conventional methods can achieve effective data compression and noise suppression under constrained processing capabilities and energy budgets, ensuring real-time performance and measurement reliability in miniaturized and low-power system architectures.

**Fig. 8:**
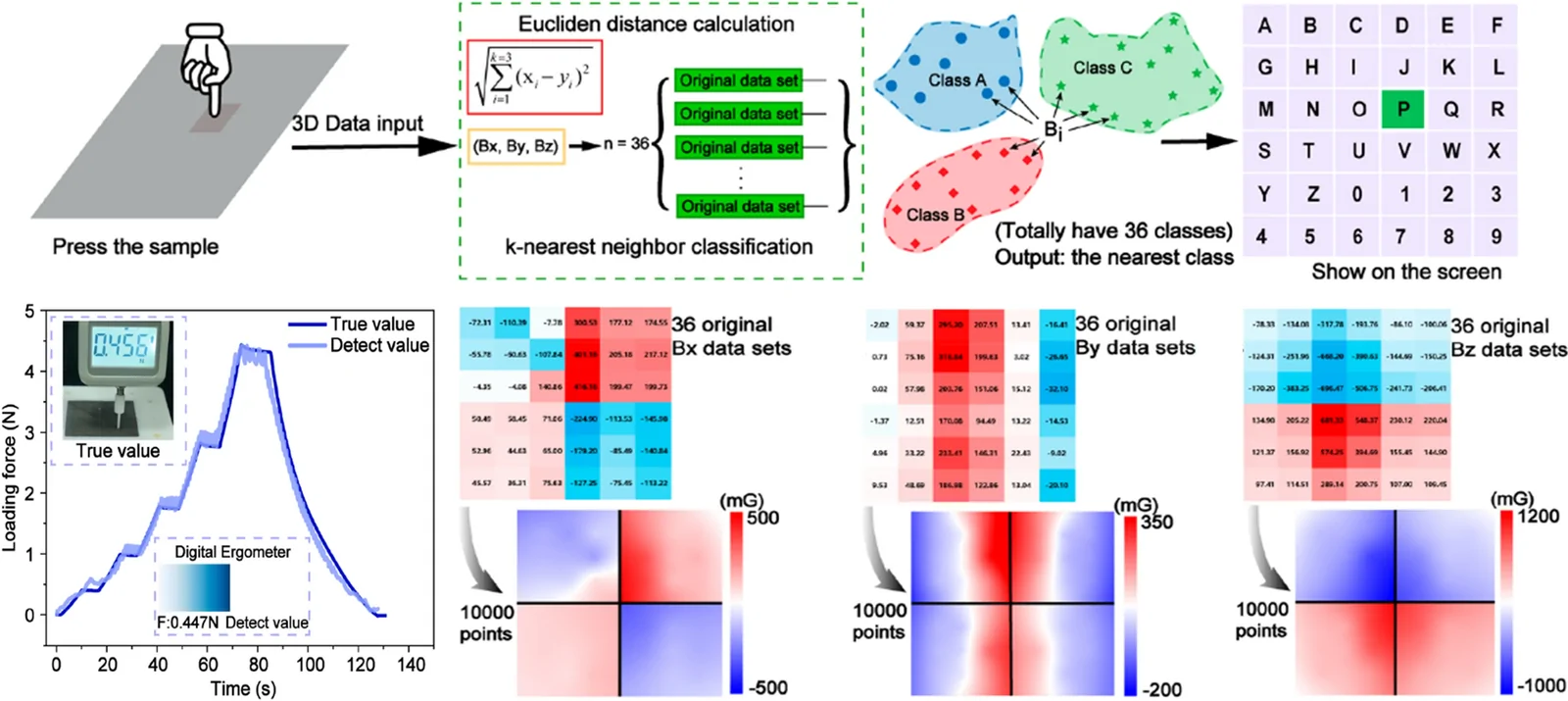
Schematic of the structure and working principle of a wireless flexible magnetic tactile sensor. The system converts external force location and magnitude into magnetic signals through an integrated Hall sensor and decodes the three-dimensional magnetic field data using clustering algorithms, enabling large-area, super-resolution tactile perception. This approach can be applied to tactile feedback in surgical robotics [109]. Reproduced with permission. Copyright 2022, ACS Nano

In wireless monitoring of tactile and pulse signals, Hu et al. [109] (Fig. 8) proposed a high-precision localization and force decoupling method based on a flexible magnetic tactile system. The system uses a single Hall sensor as the core unit, acquiring stable three-axis magnetic signals via high-precision sampling and basic noise reduction. Position and force are independently inferred through interpolation and a lightweight model, achieving millimeter-level localization accuracy with total power consumption of tens of milliwatts, compatible with low-power wireless transmission and skin-mounted continuous monitoring. The lightweight signal processing and model structure reduce local computational load, avoiding disruption to the wireless module’s energy stability. By contrast, Ma et al. [110] adopted a more traditional, computationally lightweight signal processing pathway to address the difficulty of extracting weak pulse features from noisy backgrounds without overwhelming the local power supply. Conductive micro-pyramid pressure sensors were used to collect pulse waveforms; instead of transmitting the entire raw waveform directly, key parameters including period and amplitude were extracted via Fourier analysis and peak detection. Linear filtering and thresholding realized real-time pulse waveform reconstruction. This workflow avoids complex modeling and high computational requirements, facilitating integration with low-power wireless nodes. The device exhibits high sensitivity and rapid response in low pressure range, good linearity and low hysteresis in mid pressure range, enabling stable capture of subtle pulse variations.

While conventional signal processing algorithms enable efficient feature extraction with limited training data in diverse physiological monitoring scenarios, they remain prone to performance degradation caused by noise and environmental fluctuations under dynamic operating conditions. Furthermore, the inherent processing steps including filtering and classification impose substantial computational and energy burdens on low-power wearable and implantable devices. To overcome these limitations, future research ought to prioritize lightweight processing modules, adaptive parameter tuning for motion artifact suppression, and small-scale edge computing for online self-calibration. Such developments will facilitate truly continuous, energy-efficient, and reliable operation of flexible health monitoring systems in real-world applications.

#### Machine Learning- and Neural Network-Based Data Processing

Machine learning empowers polymer-based flexible wireless sensors to process raw signals characterized by high noise levels and susceptibility to deformation. Its core advantage lies in automatically learning adaptive features from data without manual rule definition, thereby ensuring consistent output stability despite sensor deformation, attachment loosening, or environmental interference. The workflow typically comprises two interconnected stages: the offline stage focuses on model training, utilizing raw or lightly preprocessed datasets to capture spatial structures, temporal variations, and inter-channel correlations for constructing a generalized representation model; the online stage involves compressing and deploying the trained model to edge devices, enabling real-time inference with minimal computational overhead for tasks such as event recognition, anomaly detection, or signal reconstruction. The value of such methods is particularly pronounced in wireless and low-power systems. Directly extracting meaningful features from raw waveforms diminishes the reliance on complex front-end processing, thereby reducing the local computational load. Meanwhile, the model can transform high-dimensional signals into compact representations, enabling the transmission of only key features and thus alleviating the wireless communication burden at the source, ultimately enhancing the efficiency of long-term continuous monitoring.

**Fig. 9:**
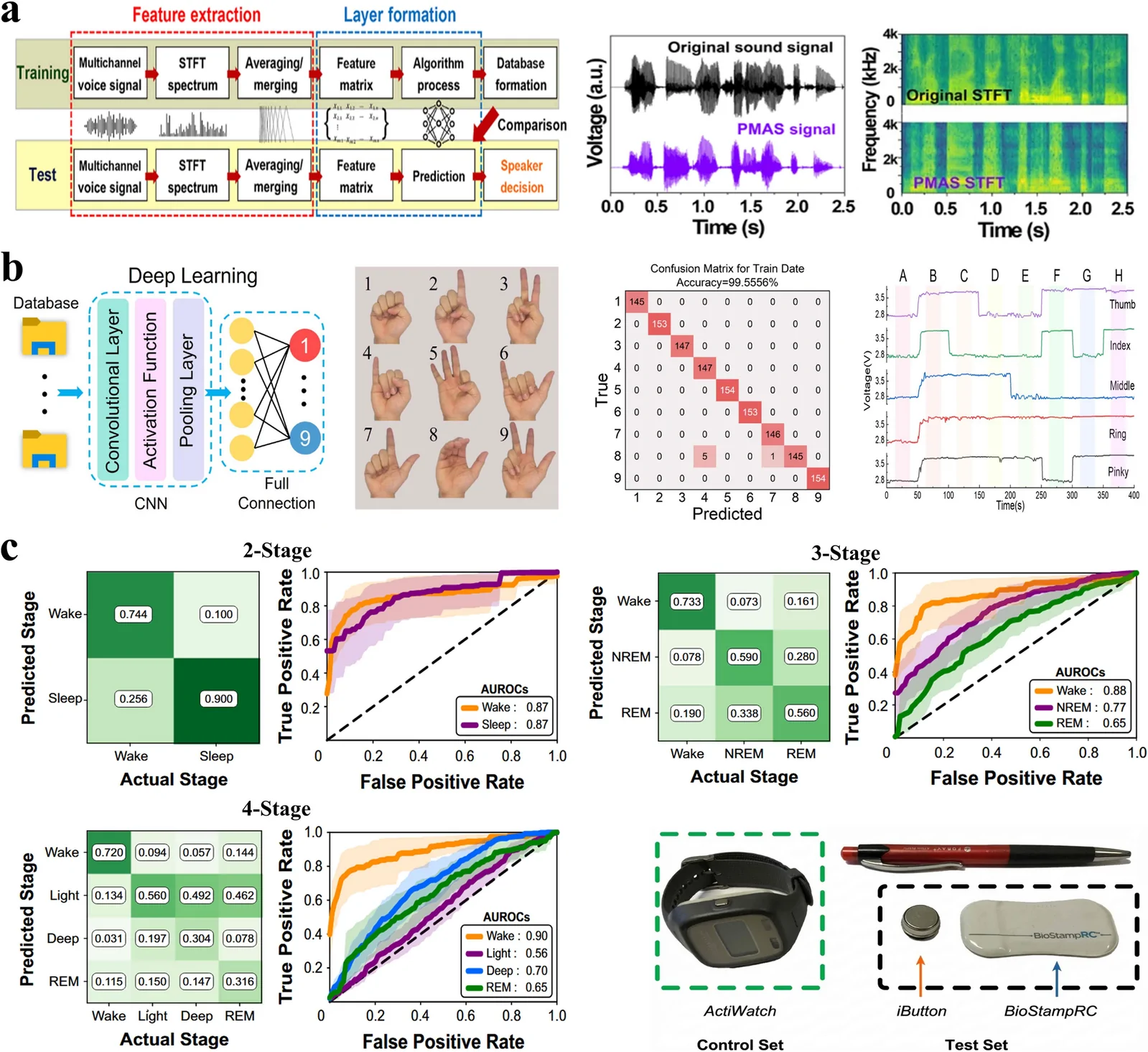
**a** Data processing framework of a multi-channel piezoelectric mobile acoustic sensor (PMAS). The flowchart illustrates feature extraction using short-time Fourier transform (STFT) and layer formation for speaker decision making, alongside comparisons of original and PMAS sound signals and their STFT spectra [111]. Reproduced with permission. Copyright 2021, Science Advances. **b** Application of a CNN deep learning model for flexible sensor signal processing. The network utilizes convolutional and pooling layers to classify multi-finger voltage signals, achieving high-accuracy (99.56%) recognition of 9 distinct hand gestures [112]. Reproduced with permission. Copyright 2025, Chemical Engineering Journal. **c** Automatic sleep stage recognition utilizing multimodal wireless wearable sensors. The performance is detailed through confusion matrices and receiver operating characteristic (ROC) curves for 2-stage (Wake/Sleep), 3-stage, and 4-stage classification models, comparing a test set (iButton, BioStampRC) against a control set (ActiWatch) [113]. Reproduced with permission. Copyright 2019, npj Digital Medicine

In practical applications, polymer-based flexible wireless sensors often confront complex signal processing challenges such as environmental background noise, multi-channel spatial crosstalk, and motion-induced baseline drift. To address these specific issues, machine learning models are deployed not merely as classifiers, but as targeted signal optimization tools. For instance, to overcome the severe interference of acoustic background noise in dynamic speech-based health monitoring, Wang et al. [111] (Fig. 9a) developed a lightweight speech processing and voiceprint recognition pipeline based on a biomimetic multi-resonance structure. The system employs a simplified analog front-end combined with wavelet and spectral subtraction denoising to achieve high signal-to-noise ratio (SNR) acquisition, while extracting key acoustic textures from multiple resonant frequency bands. Using a lightweight ResNet with approximately 1.9 million parameters, the system successfully extracts key acoustic textures from mixed noises, enabling low-power inference on mobile devices with recognition rates above 96%.

To tackle the spatial‐temporal crosstalk and long-term signal drift inherent in multi-channel flexible electronic skins, Zhang et al. [112] (Fig. 9b) proposed a targeted workflow. Here, variational mode decomposition (VMD) and low-pass filtering were specifically applied to separate low-frequency baseline drift from high-frequency physiological components. Subsequently, 1D-convolutional neural network (CNN) and CNN-LSTM networks were utilized to decode the complex spatial dependencies and temporal dynamics of the resulting 125-dimensional multimodal features, enabling millisecond-level gesture recognition and pulse/respiration analysis.

Furthermore, addressing the challenge of individual variability in long-term continuous monitoring, Boe et al. [113] (Fig. 9c) integrated accelerometer, ECG, and temperature signals. A cascaded XGBoost-LightGBM structure was employed to adaptively evaluate feature importance and select inputs based on population characteristics, thereby circumventing the need for standardized multi-lead recordings. Additionally, to resolve the conflict between high-frequency sampling requirements and strict latency/power limits in wireless nodes, Yao et al. [114] developed a low-latency sign language recognition system. Transient features of the cracks were extracted to enable adaptive baseline tracking for effective drift suppression. The system integrates these morphological features with a lightweight temporal CNN of approximately 128 k parameters, achieving over 98% accuracy with only 6-ms inference latency. It is suitable for monitoring joint micro-movements, Parkinsonian tremors, and laryngeal micro-strains. Together, these studies demonstrate the synergistic strategy of optimized analog front-ends and digital intelligent processing, enabling high-precision, real-time wireless physiological monitoring under low-power conditions.

Deep neural networks and machine learning techniques demonstrate exceptional capability in extracting nonlinear correlations from high-dimensional, multimodal physiological signals. These approaches deliver robust environmental adaptability, and enable the compression of complex raw data into high-level abstract features directly at the edge node, which substantially alleviates the burden of wireless data transmission.

Nevertheless, their practical deployment is severely hindered by multiple interconnected constraints: Flexible sensors are inherently susceptible to long-term signal drift and material degradation, model generalization performance is restricted by heavy data dependency and pronounced inter-individual physiological variability, and edge computing nodes are subject to stringent limitations in computational power and energy supply [115].

To surmount these critical bottlenecks, future research efforts should prioritize three core directions. The first focuses on developing lightweight neural networks tailored for low-power edge nodes, with architectural optimization strategies including network pruning, parameter quantization, and knowledge distillation. The second centers on implementing online adaptive modeling frameworks, which can continuously calibrate and adjust to real-time contact variations and external environmental disturbances. The third involves leveraging edge computing architectures to realize real-time anomaly detection and event-triggered data transmission, thereby striking an optimal trade-off between communication energy consumption and target recognition accuracy.

Table 4 organizes existing methods and performance metrics and highlights the divergence in method selection and system integration, providing a basis for future research on cross-method and cross-architecture comprehensive optimization.

**Table 4:** Summary of data processing techniques for polymer-based flexible wireless sensors

Data Processing Category	Typical Method	Processing Objective	Processing Location	Signal Processing Advantages and Disadvantages	Output Performance	Health Monitoring Application	Refs
Simple Data Processing	Morse code decoding	Signal decoding (abnormal respiration)	On-node	Real-time, portable; limited to predefined patterns	Response < 150 ms; Power < 1 mW	Sleep monitoring, respiratory feedback	[116]
	Low-pass filtering	Signal denoising	On-node	Simple, low-power; may lose high-frequency information	Error < 5%; Response < 150 ms; Power ~ 1 mW	Respiratory monitoring (normal/apnea)	[117]
	Flexible signal amplifier	Noise suppression and amplification	On-node	High SNR, flexible integration; requires calibration	Noise < 1 µV (2–30 Hz); × 30 amplification; Power ~ 2 mW	EEG/ECoG, pulse monitoring	[118]
Traditional Algorithm-Based Processing	Frequency-domain and spectral analysis methods	Strain-resistance linear fitting	On-node/Edge	High accuracy, low complexity; requires calibration	R^2^ = 0.997; Latency ~ 60 ms; Power ~ 2–3 mW	Respiration, gait analysis	[119]
	Time–frequency analysis and filtering techniques	Blood pressure prediction (SBP/DBP/MAP)	Edge	Handles multicollinearity, stable, interpretable; requires samples	R^2^ = 0.983/0.847/0.909; Latency ~ 80 ms; Power ~ 5 mW	Real-time BP monitoring	[120]
	Time–frequency analysis and filtering techniques	Classification (sarcopenia screening)	Edge	High accuracy, nonlinear robustness; requires training data	Accuracy 93.65%; Latency ~ 100 ms; Power ~ 6–8 mW	Muscle function assessment	[121]
	Data compression and sparse representation approaches	Compressed sensing/reducing sampling time	On-node/Edge	Reduces data volume and sampling time; requires high-complexity reconstruction	Latency ~ 200 ms; Sampling time reduced by 40% (RR accuracy 95.54%)	Pressure distribution, respiratory monitoring	[122]
Machine Learning/Neural Network-Based Processing	ANN	NAA concentration prediction	Edge	Intelligent, real-time output; requires training	LOD 1.6 nM; Latency ~ 50–100 ms; Power ~ 10–15 mW	Food safety and environmental monitoring	[123]
	Random Forest classifier	Thermal comfort prediction (cool/neutral/hot)	Edge	Robust, high accuracy, embeddable; higher computational cost	Accuracy 93.9%; Latency ~ 100–150 ms; Power ~ 15 mW	Skin temperature and thermal comfort monitoring	[124]
	Deep CNN	Posture recognition (16 classes)	Edge/Cloud	Automatic feature extraction, high accuracy; requires training	Accuracy 95%; Latency ~ 200–300 ms; Power ~ 50 mW	Human motion detection	[125]
	CNN-GRU network	Color-strain prediction and temperature correction	Edge/Cloud	High precision, self-calibration; data intensive	R^2^ = 0.998; MSE = 0.018; Latency ~ 250 ms; Power ~ 60 mW	Joint motion monitoring	[126]

## Fabrication Methods

The overall performance of flexible sensors is highly dependent on fabrication processes, which directly affect the microstructural morphology and electrical response of the sensing layer and determine device mechanical flexibility, long-term stability, and multifunctional module integration capability [127, 128]. Besides, fabrication processes further influence electromagnetic coupling and impedance matching between the sensing unit and modules for wireless transmission, energy harvesting and signal conditioning.

### In Situ Polymerization Strategy

In situ polymerization enables the direct formation of active networks on flexible substrates. Rather than detailing the fundamental chemical mechanisms, this section focuses on how in situ processing parameters directly govern the structural and mechanical properties of the sensors. Specifically, crosslinker concentration dictates the maximum stretchability of the polymer network, while curing conditions (e.g., UV irradiation intensity or thermal curing temperature) control the film thickness and spatial uniformity. A key fabrication advantage of the in situ interfacial co-growth mechanism is the dramatic enhancement of interfacial adhesion between the active network and the substrate, effectively preventing delamination under large mechanical strains [129].

**Fig. 10:**
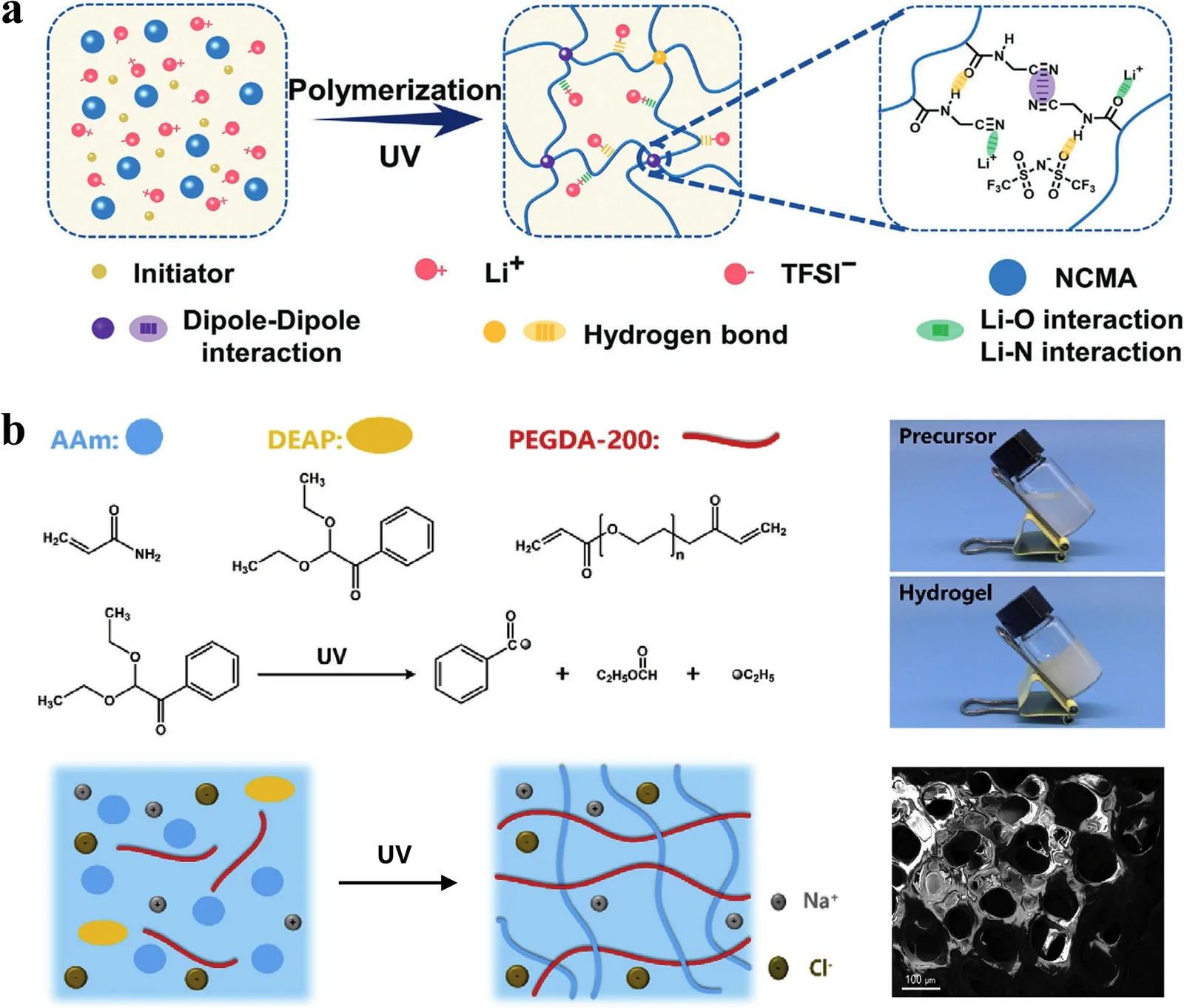
**a** Formation of a deep eutectic solvent by heating a mixture of N-cyano-methylacrylamide (NCMA) and lithium bis(trifluoromethanesulfonyl)imide (LiTFSI), followed by one-step photopolymerization with a photoinitiator to prepare a reversible physical network gel [130]. Reproduced with permission. Copyright 2023, Advanced Materials. **b** One-step in situ polymerization of PAAm hydrogel by mixing AAm, photoinitiator DEAP, and crosslinker PEGDA-200 in NaCl solution, followed by UV irradiation [131]. Reproduced with permission. Copyright 2020, Nano Energy

Recent studies demonstrate how modifying precursor formulations and curing steps can optimize these target parameters. For instance, Yao et al. [130] (Fig. 10a) utilized a one-step photopolymerization strategy based on deep eutectic solvents. By eliminating chemical crosslinkers to form a reversible physical network, this approach yielded gels with both high stretchability and exceptionally strong interfacial adhesion (624.9 kPa) on target substrates. Similarly, Hang et al. [131] (Fig. 10b) achieved highly stretchable, self-healing networks via one-step in situ UV polymerization, rapidly forming a 3D porous structure that supports high structural complexity and accommodates severe physical deformations without rupture.

While providing robust interfacial integration, in situ polymerization faces inherent challenges in spatial control. The high sensitivity to polymerization kinetics often causes spatial inhomogeneities, limiting the ultimate patterning precision and thickness uniformity across large areas [132]. To address this, advanced fabrication trends are shifting toward integrating in situ polymerization with 3D printing and digital lithography. These hybrid approaches bypass the limitations of traditional bulk gelation, offering the high patterning precision and fine structural complexity required for next-generation flexible matrices.

### 3D/4D Printing

3D printing techniques dictate the structural complexity and patterning precision of flexible matrices through strict parameter regulation [133, 134]. In direct ink writing (DIW), controlling extrusion pressure and printing speed effectively tunes the structural porosity and stretchability of high-viscosity materials [135, 136]. Light-based techniques, such as digital light processing (DLP) and projection stereolithography (PSL), provide sub-100 μm patterning precision; optimizing UV intensity and layer thickness prevents over-curing, thereby preserving the mechanical compliance of the printed network. Building on this, 4D printing introduces time-dependent deformability. By programming shape-memory polymers above their glass transition temperature, the printed networks achieve reversible deformations, further enhancing the stretchability and dynamic structural adaptability of the matrix without compromising initial patterning precision.

**Fig. 11:**
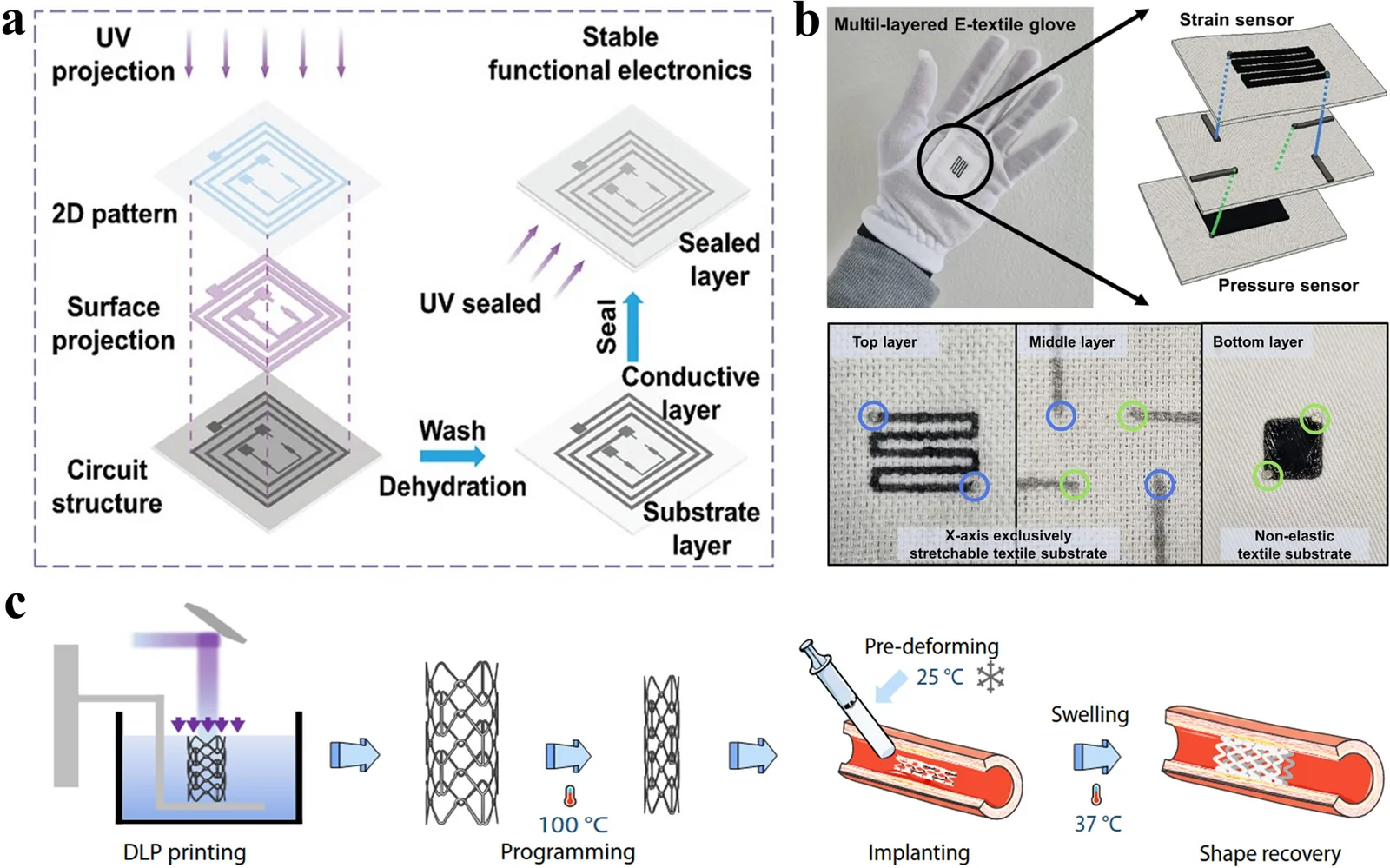
**a** Fabrication of a highly conductive hydrogel via PSL 3D printing. Two-dimensional antenna patterns are printed on a photopolymerizable elastic substrate using PSL [137]. Reproduced with permission. Copyright 2024, Advanced Materials. **b** DIW 3D-printed multifunctional wearable electronic textile platform. Multi-ink DIW printing enables bidirectional interconnects and multilayer structural construction [138]. Reproduced with permission. Copyright 2025, npj Flexible Electronics. **c** DLP 4D printing of a micelle-reinforced shape-memory polymer (SMP) system for the fabrication of personalized minimally invasive vascular scaffolds [139]. Reproduced with permission. Copyright 2025, Chinese Journal of Polymer Science

These direct correlations between printing processes and physical capabilities are evident in recent studies. For example, Sun et al. [137] (Fig. 11a) utilized PSL planar projection to achieve high patterning precision (≈ 50 μm resolution) and controlled conductive layer thickness (≈ 100 μm). The process also integrated a photopolymerizable sealing layer that formed covalent bonds with the substrate, ensuring robust interfacial adhesion and structural integrity under cyclic stretching. Addressing multi-material structural complexity, Pak et al. [138] (Fig. 11b) employed multi-ink DIW to construct multilayer architectures. By optimizing extrusion pressure and nozzle speed, they achieved a line width resolution of ≈ 440 μm, successfully managing complex spatial layouts and bidirectional interconnects on uneven substrates.

Furthermore, integrating shape-memory materials with high-resolution printing drastically expands the structural complexity achievable in flexible networks. Liu et al. [139] (Fig. 11c) demonstrated DLP 4D printing of a micelle-reinforced shape-memory polymer. Leveraging DLP’s planar exposure, they rapidly fabricated complex 3D lattice-like architectures with high patterning precision (≈ 40 μm) and uniform layer thickness (50–100 μm). The thermal programming of these shape-memory structures allows for dynamic volumetric transformation while maintaining exceptional material deformability.

While 3D and 4D printing excel in creating intricate geometries, maintaining precise patterning and strong interfacial adhesion across distinct layers remains challenging. Variations in ink rheology, curing kinetics, and thermal expansion often compromise interlayer bonding and dimensional accuracy. Future fabrication refinements must focus on robust multi-material processing windows to minimize post-processing defects and secure the structural consistency of these complex flexible matrices.

### Photolithography

Photolithography and localized energy-induced microfabrication (e.g., laser direct writing) offer unparalleled control over the patterning precision and thickness of flexible matrices. In mask-based photolithography, optimizing spin-coating speed and UV exposure dose is critical to dictate the final thickness, aspect ratio, and patterning precision, while preventing edge roughness. Similarly, in maskless laser direct writing, tuning the laser power and scanning speed dictates the localized energy fluence. An optimal fluence yields a highly porous network that maintains stretchability, whereas excessive energy causes structural embrittlement that drastically degrades it [140]. Crucially, these high-resolution techniques allow for exact geometric consistency and the seamless co-fabrication of multilayer structures, considerably increasing the structural complexity achievable on a single flexible substrate.

**Fig. 12:**
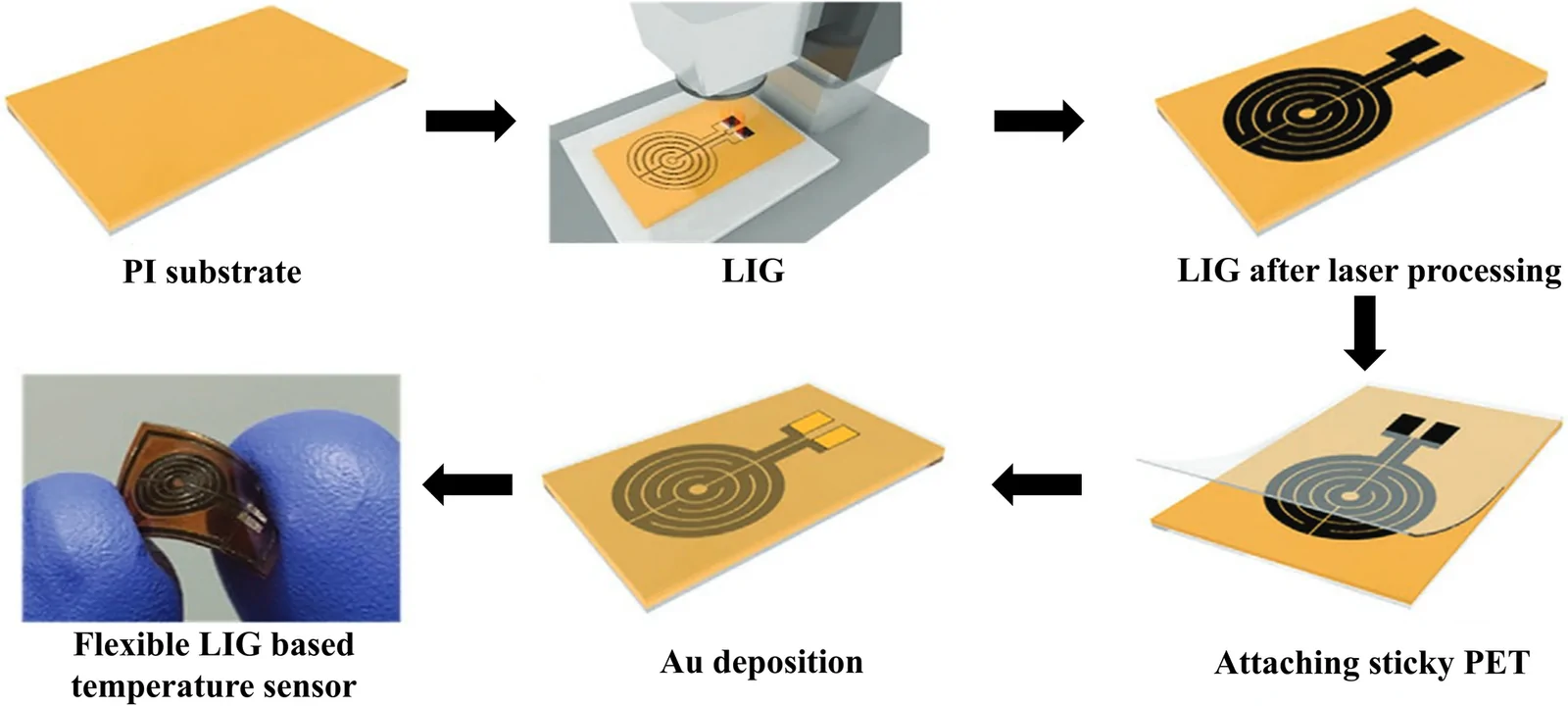
Direct writing on Kapton polyimide films using laser-induced carbonization technology [142]. Reproduced with permission. Copyright 2020, Advanced Materials Technologies

Recent studies highlight how these patterning techniques enhance structural properties without compromising flexibility. Xu et al. [141] utilized laser ablation to create regularly distributed, ordered microstructures on a conductive fabric substrate. This maskless approach considerably improved the patterning precision and effective contact area while preserving the inherent stretchability and mechanical compliance of the textile. By integrating a highly porous piezoresistive medium, the resulting multiscale microstructure accommodated reversible deformations under mechanical loading. Similarly, Gandla et al. [142] (Fig. 12) demonstrated the rapid construction of fine structures via laser-induced carbonization. By directly writing carbonized networks on polyimide films with nanosecond pulsed lasers, they eliminated the need for photoresist masks and wet etching. This simplified approach directly improved interfacial adhesion, avoiding the delamination risks associated with harsh chemical treatments. The resulting laser-induced graphene architectures exhibited excellent structural stability, highlighting the advantage of direct-writing techniques in achieving fine patterning precision and enabling the stable encapsulation of functional layers.

Overall, mask-based photolithography delivers sub-100-μm resolution, facilitating the precise stacked integration of multilayer modules and enhancing overall structural complexity. Conversely, maskless laser processing enables rapid, flexible patterning compatible with non-planar and textile surfaces. However, traditional photolithography frequently exhibits poor mechanical compatibility with flexible substrates, inducing internal stress that compromises stretchability and interfacial adhesion. Meanwhile, laser etching faces challenges in depth uniformity, where thermal impacts can cause non-uniform carbonization or substrate warping. Future advancements must focus on developing mechanically compliant photoresists and low-thermal-impact scanning protocols to ensure high patterning precision and robust interfacial adhesion for highly integrated flexible matrices.

### Screen Printing

Screen printing dictates the thickness and patterning precision of flexible matrices through the precise regulation of mesh count, squeegee pressure, and printing speed [143–145]. A lower mesh count produces thicker printed layers (> 20 µm) that reduce sheet resistance but increase susceptibility to strain-induced microcracking, thereby degrading overall stretchability. Conversely, higher mesh counts (300–400 mesh) paired with optimized thixotropic ink viscosity (10–50 Pa·s) create thin, conformal percolation networks that considerably improve the mechanical compliance and stretchability of the structures. Building multilayer architectures via sequential printing requires carefully controlled thermal curing (60–150 °C) to ensure complete solvent evaporation and strong interfacial adhesion, mitigating structural delamination under complex mechanical deformations. Furthermore, sequential printing inherently involves alignment registration errors (typically ± 20–50 µm), which directly impact the achievable structural complexity and patterning precision.

**Fig. 13:**
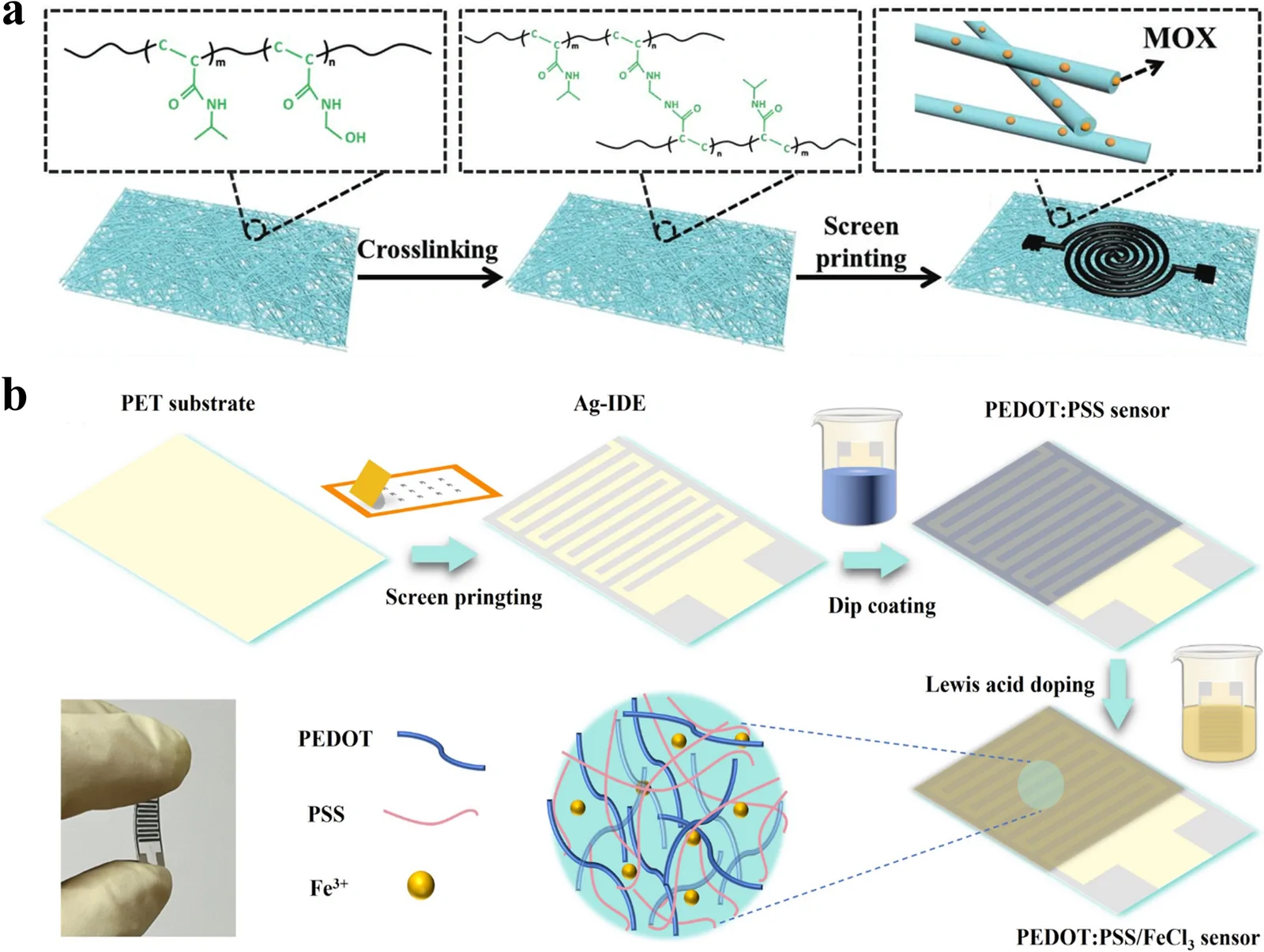
**a** Flexible nanonetwork temperature sensor fabricated on a thermoresponsive PNIPAM copolymer substrate, featuring spiral electrodes patterned via screen printing of conductive silver ink [146]. Reproduced with permission. Copyright 2019, Advanced Functional Materials. **b** Flexible high-sensitivity ammonia sensor based on a PET substrate, with silver interdigitated electrodes formed by screen printing and a PEDOT:PSS sensing layer deposited between the electrodes; the device was further doped with FeCl_3_ to enhance performance [147]. Reproduced with permission. Copyright 2024, Chemical Engineering Journal

Recent literature illustrates how structural design combined with screen printing can optimize these target mechanical parameters. Gong et al. [146] (Fig. 13a) utilized screen printing to pattern a continuous conductive nanonetwork film on a crosslinked electrospun substrate. By screen-printing silver paste into a specific helical structural design, they effectively dispersed mechanical strain, maintaining the continuity of conductive pathways under bending and thereby preserving excellent stretchability and strong interfacial adhesion with the underlying matrix. Similarly, Lv et al. [147] (Fig. 13b) combined screen printing with solution coating to manage multi-material architectures. Silver interdigitated electrodes were screen-printed on a flexible substrate, followed by the deposition of a thin film in the inter-electrode regions. This strategy enabled the formation of highly uniform electrode arrays, demonstrating how screen printing can reliably construct the structural complexity and patterning precision required for integrating distinct functional layers without compromising mechanical flexibility.

While screen printing offers a rapid approach for fabricating continuous conductive networks, its ultimate patterning precision is inherently restricted by mesh limitations and ink rheology. These constraints hinder the fabrication of high-density, micro-/nanoscale features required for advanced structural complexity. Furthermore, critical process variables such as ink viscosity strongly influence film thickness uniformity and interlayer interfacial adhesion. To overcome these limitations, future efforts must focus on hybridizing screen printing with high-resolution techniques and optimizing advanced nanostructured inks to enhance patterning precision and ensure robust interfacial adhesion under dynamic strain.

### Inkjet Printing

Inkjet and aerosol jet printing (AJP) provide non-contact, digitally programmable deposition, making them highly effective for dictating the patterning precision and thickness of flexible matrices. In standard inkjet printing, precise regulation of drop spacing and substrate temperature (30–80 °C) is crucial to control solvent evaporation and suppress the “coffee-ring effect.” Inadequate parameter control leads to uneven cross-sectional thickness and rough edges, which serve as stress concentration sites that severely degrade stretchability and interfacial adhesion under external strain [148]. Furthermore, post-printing sintering dictates the structural continuity of the network; incomplete sintering causes defects that compromise electrical performance. Expanding beyond planar limitations, AJP leverages aerodynamic focusing to deposit atomized aerosols onto complex, non-planar geometries, achieving line-width patterning precision as fine as 10 μm and considerably enhancing the structural complexity of the printed matrix.

**Fig. 14:**
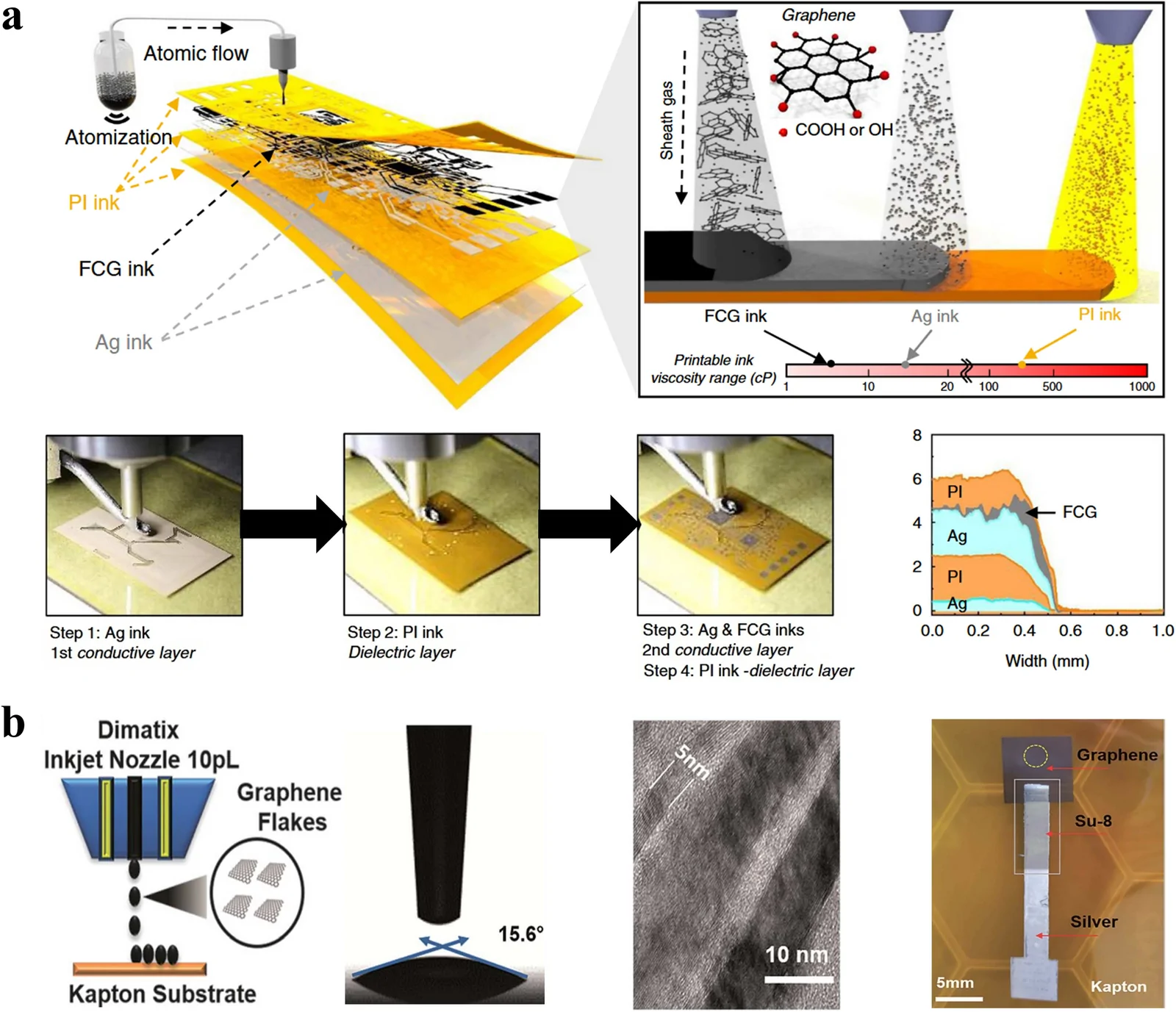
**a** Fully printed nanomembrane wireless bioelectronic sensor fabricated via aerosol jet printing [149]. Reproduced with permission. Copyright 2020, Nature Communications. **b** Schematic of multilayer graphene-based flexible electrodes fabricated by inkjet printing. By optimizing ink rheology and multilayer printing parameters, structurally uniform and highly reproducible flexible electrochemical electrodes were achieved on a Kapton substrate [150]. Reproduced with permission. Copyright 2020, RSC Advances

Recent advancements demonstrate the capability of these techniques to optimize fine structural features. Kwon et al. [149] (Fig. 14a) adopted AJP to fabricate a multilayer nanomembrane architecture. By regulating atomization modes, they deposited inks across a wide viscosity range with micrometer-scale patterning precision, constructing multilayer interconnects with an overall thickness of merely several micrometers and improving structural complexity without adding bulk. Similarly, Pandhi et al. [150] (Fig. 14b) utilized conventional inkjet printing to fabricate multilayer thin films. By optimizing droplet volume, printing speed, and substrate temperature, they effectively controlled ink rheology and suppressed the coffee-ring effect, achieving highly uniform thickness and reproducible patterning precision across the substrate. Bihar et al. [151] further highlighted the scalability of this maskless approach by sequentially constructing functional layers through multilayer ink deposition. This process eliminated the need for complex photolithography while maintaining the high patterning precision and structural complexity required for sequential layer integration.

While inkjet-based techniques provide a template-free approach for achieving high patterning precision (20–50 µm for standard inkjet), they face distinct structural challenges. The inherently limited thickness of single printed layers complicates multilayer stacking, imposing stringent requirements on alignment control and interlayer interfacial adhesion. Overcoming these limitations requires optimizing ink formulations and advancing multi-material co-printing protocols to ensure robust interfacial adhesion and consistent structural complexity under dynamic mechanical deformation.

### Electrospinning

Electrospinning is a highly efficient deposition method for fabricating continuous nanofiber networks with exceptional stretchability and high porosity. Rather than detailing the fluid dynamics of the Taylor cone, this section focuses on how process parameters dictate network morphology. Specifically, applied voltage and solution concentration synergistically dictate the final fiber diameter and overall layer thickness. Furthermore, maintaining an optimal collection distance and ambient humidity is critical to ensure complete solvent evaporation; incomplete evaporation results in fused fiber intersections that rigidly lock the network, drastically increasing mechanical hysteresis and degrading stretchability. Collection methods also define macroscopic structural complexity; for instance, utilizing a high-speed rotating drum or parallel plate electrodes aligns fibers along specific axes, imparting targeted mechanical anisotropy to the flexible matrix.

**Fig. 15:**
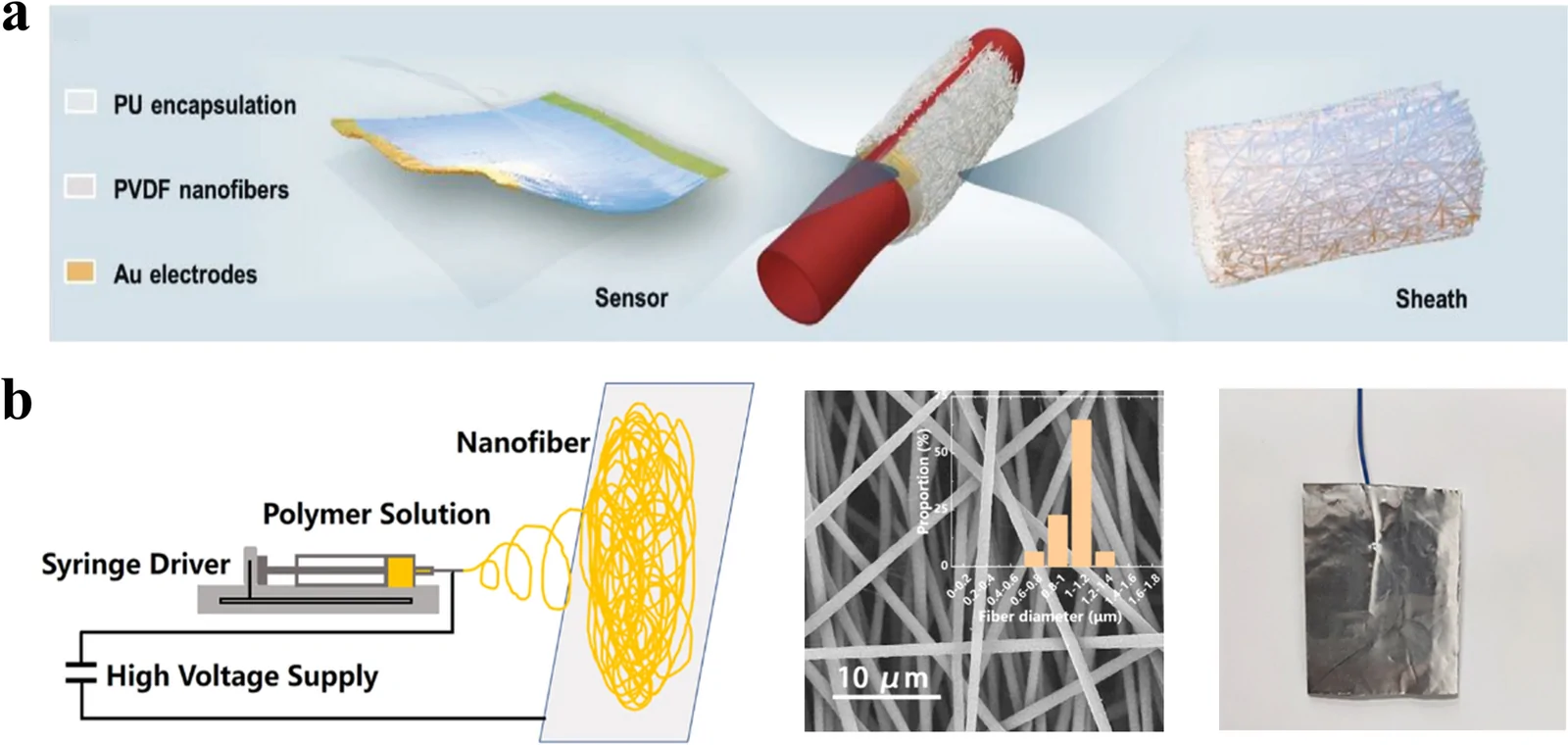
**a** Schematic of the fabrication and application of a PVDF/PU flexible vascular electronic system based on electrospinning. Parallel plate electrodes were used to align PVDF nanofibers and enhance *β*-phase content, while oxygen plasma-modified PU sheaths provided biocompatibility and mechanical compliance [152]. Reproduced with permission. Copyright 2024, Small. **b** Schematic of the fabrication and application of a PVDF-based flexible piezoelectric sensor via electrospinning. Control of the electric field and spinning rate allowed aligned PVDF nanofibers with enhanced *β*-phase content, resulting in a sensor with excellent piezoelectric performance and mechanical stability [153]. Reproduced with permission. Copyright 2020, ACS Applied Materials and Interfaces

Recent studies demonstrate this precise microstructural control. Tang et al. [152] (Fig. 15a) utilized parallel plate electrodes during electrospinning to produce highly aligned nanofiber networks. By precisely tuning the electrospinning parameters, they achieved uniform fiber alignment that enhanced the material’s intrinsic physical response while maintaining high stretchability and mechanical compliance. Similarly, Liu et al. [153] (Fig. 15b) leveraged the electrospinning process to fabricate uniform nanofiber membranes, utilizing the electric field to induce molecular dipole alignment. This structural tuning illustrates how electrospinning parameters directly govern the internal structural complexity without compromising the mechanical flexibility of the resulting matrix.

While electrospinning excels in creating highly porous and stretchable networks, its inherent random deposition heavily restricts ultimate patterning precision. Variations in local porosity complicate the fabrication of high-density micro-/nanoscale features. Furthermore, the spun networks often suffer from weak interfacial adhesion with adjacent functional layers, and residual solvents can accelerate interfacial delamination under mechanical strain [154]. To overcome these limitations, future strategies must integrate coaxial electrospinning or hybridize the process with high-resolution techniques (such as laser direct writing or inkjet printing) to concurrently enhance patterning precision and secure strong interfacial adhesion within complex multilayer architectures.

Table 5 provides a comprehensive summary of the various flexible sensor fabrication strategies discussed in this section. Representative examples of polymer-based flexible wireless sensors corresponding to each method are also summarized. The table systematically compares the characteristics of fabrication technique, typical material systems, key process parameters, and representative performance metrics, offering a clear reference for selecting appropriate fabrication strategies based on specific application requirements.

**Table 5:** Summary of representative polymer-based flexible wireless sensors based on different fabrication strategies

Fabrication Technique	Advantages and Limitations	Key Process Parameters	Typical Material Systems	Representative Performance Metrics	Refs
Screen printing	Advantages: Robust, battery/chip-free; eliminates fragile rigid-soft interfaces Limitations: Limited wireless range (~ 20–25 mm); vulnerable to strain-induced electrical variations	Curing: 110 °C for 10 min Coating: 2 mg mL^−1^ SWNT solution Dimensions: 2 mm sensor width; 100 µm SEBS thickness	Conductor: Ag flake/elastomer ink Substrate/Dielectric: SEBS elastomer Sensing Layer: Thickness-gradient SWNT network	Stretchability: Functional up to 50% strain Sensitivity: GF = 201.6 (at 5% strain) Resistance: Conductor < 50 mΩ/sq; initial sensor resistance 357.3 Ω	[155]
	Advantages: Real-time, highly sensitive, and chip-free monitoring at a single frequency Limitations: Limited wireless range (~ 24 mm); highly sensitive to coil misalignment	Frequency: Fixed at 13.56 MHz Coil Design: Double-layer layout optimized via winding direction and through-hole connections	Antenna: Double-sided Cu on PI film Deformable Layer: Breathable elastic fabric Absorber: High-permeability ferrite film	Sensitivity: 1.23 × 10^–2^ kPa^−1^ (0–15 kPa) Resolution: 54 Pa Response/Recovery: 53/26 ms Durability: 10,000 cycles (at 4.8 kPa)	[156]
Inkjet printing	Advantages: Low-cost and scalable using commercial printers and inks Limitations: Vulnerable to high humidity (> 70% RH); requires encapsulation	Electrode Spacing: 200 µm AgNP Treatment: Drying (60 °C, 10 min) achieves maximum sensitivity Sensing Thickness: 3 layers (170 nm)	Flexible PET (substrate), AgNP ink (electrodes), pure PEDOT:PSS ink (sensing layer)	Temperature: 20–70 °C range; -0.8%/°C sensitivity Humidity: 20%–70% RH range; 1.7%/% RH sensitivity	[157]
	Advantages: Ultrahigh sensitivity and durability; low-cost lithography-free process Limitations: Slight resistance hysteresis (5.2%) from PDMS viscoelasticity	Template: 60-mesh cotton spandex Coating: 3 MXene/AgNFs spray cycles Electrodes: 1 mm interdigital spacing and 0.7 mm line width	Micro-convex PDMS, MXene/AgNFs composite, PET-based flexible interdigital electrodes	Sensitivity: 191.3 kPa^−1^ (0–3.36 kPa) Response/Recovery: 80/90 ms LOD: 8 Pa Durability: 18,000 cycles (at 4.3 kPa)	[158]
3D/4D printing	Advantages: Battery-free, customizable range, excellent shock absorption Limitations: Short read distance (< 10 cm); limited ink quality factor	Fused Filament Fabrication (FFF): 0.2 mm layer/nozzle, 100% infill, 220 °C, 1.5–6 mm s^−1^ DIW: 300 µm trace, dried overnight, 100 °C/30 min cure	Ninjaflex TPU filament (1.75 mm) for the flexible substrate Silver nanoparticle-based conductive ink (Voltera Flex 2) for circuitry PI substrate (0.1 mm)	Sensitivity: -15.7 MHz/kPa (0–9 kPa) and -2.1 MHz/kPa (10–40 kPa) SNR: 113 at 2–3 GHz Repeatability: Standard deviation < 0.002 for resonant frequency changes	[159]
	Advantages: Modular design; filters skin deformation, replaceable sensors Limitations: Resolution limits stiffness; low sensitivity at < 200 kPa	Global: Layer height 0.3 mm, 100% infill, room-temperature bed Thermoplastic Polyurethane (TPU): Print speed 15 mm s^−1^, nozzle 220 °C EEL (Dielectric): Print speed 10 mm/s, nozzle 230 °C	TPU NinjaTek Cheetah (95A) for the flexible structure TPU NinjaTek Eel (90A) for the dielectric components Human skin directly acts as the reference electrode	Detection Range: Tunable from 70 to 2500 kPa Sensitivity: 0.01–0.0002 /kPa Response Time: 800 ms Linearity: R-square of 97% in the sensing zone	[160]
Electrospinning	Advantages: In-situ electrospinning poling enhances piezoelectricity Limitations: BaTiO3 NWs limited to ≤ 3 wt% for proper fiber formation	Voltage: 12 kV Working distance: ~ 12 cm Duration: 6 h Environment: Room temperature, 20%–50% RH Solvent: DMF and acetone (1:1 by weight)	Polyvinylidene difluoride (PVDF) Tetragonal BaTiO_3_ nanowires (NWs) Aluminum/copper foils and adhesive tape	Sensitivity: 0.017 kPa^−1^ Response time: 290 ms Linear pressure range: 1 to 40 kPa Max output current: ~ 105 nA (at 3 wt% NWs, 3.5 Hz) Durability: Stable after 1750 cycles	[161]
	Advantages: Ultra-thin, breathable, sweatproof, negligible crosstalk Limitations: Excess IL blocks pores; excess graphene reduces temp sensitivity	TPU spinning: 20 kV voltage, 20 cm distance, 0.1 mL min^−1^ flow rate, 1 h duration IL/TPU spinning: 20 wt% IL, 35 kV voltage, 10 cm distance, 0.005 mL min^−1^ flow rate, 20 h duration	TPU substrate, IL/TPU electrolyte, PEDOT:PSS/PVA electrodes	Pressure sensitivity: 147.19 kPa^−1^ (0–7 kPa); 4.41 kPa^−1^ (25–85 kPa) Temperature sensitivity: −0.04 °C^−1^ (25–50 °C); −0.002 °C^−1^ (50–100 °C)	[162]
In situ polymerization	Advantages: High transparency, all-climate tolerance (−40 ~ 80 °C), high toughness Limitations: Viscosity increases with IL content; high-temp evaporation	Initiation: UV irradiation for 30 min Light Source: 365 nm, 22.4 mW cm^−2^ Concentration: AAm monomer fixed at 20 wt%	Monomer: AAm Crosslinker: Methacrylated chondroitin sulfate (CSMA) Multifunctional Solvent: Hydrated ionic liquid ([EMIM]Cl and water) Initiator: 2,2-diethoxyacetophenone (DEAP)	Sensitivity: 1.66 (0–200% strain), 3.64 (200%–600% strain), and 6.78 (600%–800% strain) Ionic Conductivity: 38.86 mS cm^−1^ at 25 °C; remains functional at 3.10 mS cm^−1^ at -40 °C	[163]
	Advantages: Battery-free wireless loop; thermal-detachable (no tissue damage) Limitations: High cost, storage requirements, potential biofouling	Gelation Time: ~ 3 min (RT) Phase Transition: Detaches at ~ 40 °C Wireless Frequency: 13.56 MHz	Backbone: NIPAM/AAm; Conductive: PEDOT:PSS Initiator: H₂O₂ / Ascorbic acid	Wireless Range: 15 cm Adhesion: Drops 2 orders of magnitude via heat Durability: 10,000 cycles	[164]
Photolithography	Advantages: Integrated two-in-one design; shared functional layers Limitations: Temperature sensitivity; sensitivity-transparency trade-off	Hydrogel: LiCl/HEA/EOEOEA; PEDOT:PSS: Spin-coat (800 rpm)	Ionic hydrogel (Dielectric); PEDOT:PSS on ITO/PET (Electrode); Cu on PI (Antenna)	Sensitivity: 171 kPa^−1^ (0–60 kPa); 12 kPa^−1^ (60–250 kPa) LOD: 1.2 Pa Response Time: 5.6 ms (pressure); 0.36 s/1.08 s (EC coloring/bleaching)	[165]
	Advantages: High linearity and low hysteresis; flexible mm-scale form Limitations: Temperature dependency; requires thermistor compensation	30% biaxial prestrain; asymmetric PI layer control (14/1 μm)	Sensing: Au serpentine gauge; Support: PI/Acrylic Encapsulation: Ecoflex 00–30	Range: Target operating range of 0–60 mmHg Linearity: High pressure detection linearity of R^2^ = 0.992 Response Time: Rapid response within 200 ms	[166]

## Progress in Polymer-Based Material Systems for Health Monitoring

Driven by advancements in nanotechnology and polymer science, diverse material systems, including carbon-based materials, metals, polymers, two-dimensional materials (e.g., MXenes), and paper-based materials, have emerged with distinct electromechanical and interfacial properties. These properties not only enable versatile biosensing but also strongly dictate critical system functionalities such as wireless transmission and energy efficiency. Recent comprehensive reviews [167] highlight that flexible medical sensors are leveraging these materials to achieve unprecedented integration across material, device, and system levels. This multilevel integration is driving a paradigm shift from simple physiological tracking to complete closed-loop diagnostic-therapeutic cycles in complex clinical scenarios, including neurological monitoring, cardiovascular management, and smart wound care. For instance, in neuroengineering, Chen et al. [168] demonstrated a major breakthrough by coupling biorecognition elements with flexible transducers to enable continuous, real-time, and precise quantitative monitoring of in vivo neurological biomarkers.

### Carbon-Based Fillers in Polymer Matrices

Due to their abundant allotropes and highly tunable microstructures, carbon-based materials exhibit unique and critical functionalities in polymer-based flexible wireless sensors. Compared with conventional metals or inorganic semiconductors, carbon materials offer high electrical conductivity and flexibility, as well as good biocompatibility. They also maintain stable electrical responses under large deformations and dynamic loading. These characteristics provide invaluable advantages for low-power, weak-signal wireless applications, including skin-attached, wearable and implantable health monitoring devices.

#### Graphene and Its Derivatives

Graphene and its derivatives, including reduced graphene oxide (rGO) and laser-induced graphene (LIG), possess excellent electrical conductivity, tunable dielectric properties, and intrinsic flexibility [169, 170]. In the context of polymer-based flexible wireless sensors, these material characteristics provide stable conductive pathways and conformal skin adhesion. Rather than acting as complex system-level filters, their primary contribution lies in maintaining stable tissue-sensor interfaces, which inherently helps minimize physical motion artifacts and baseline fluctuations during continuous signal acquisition.

**Fig. 16:**
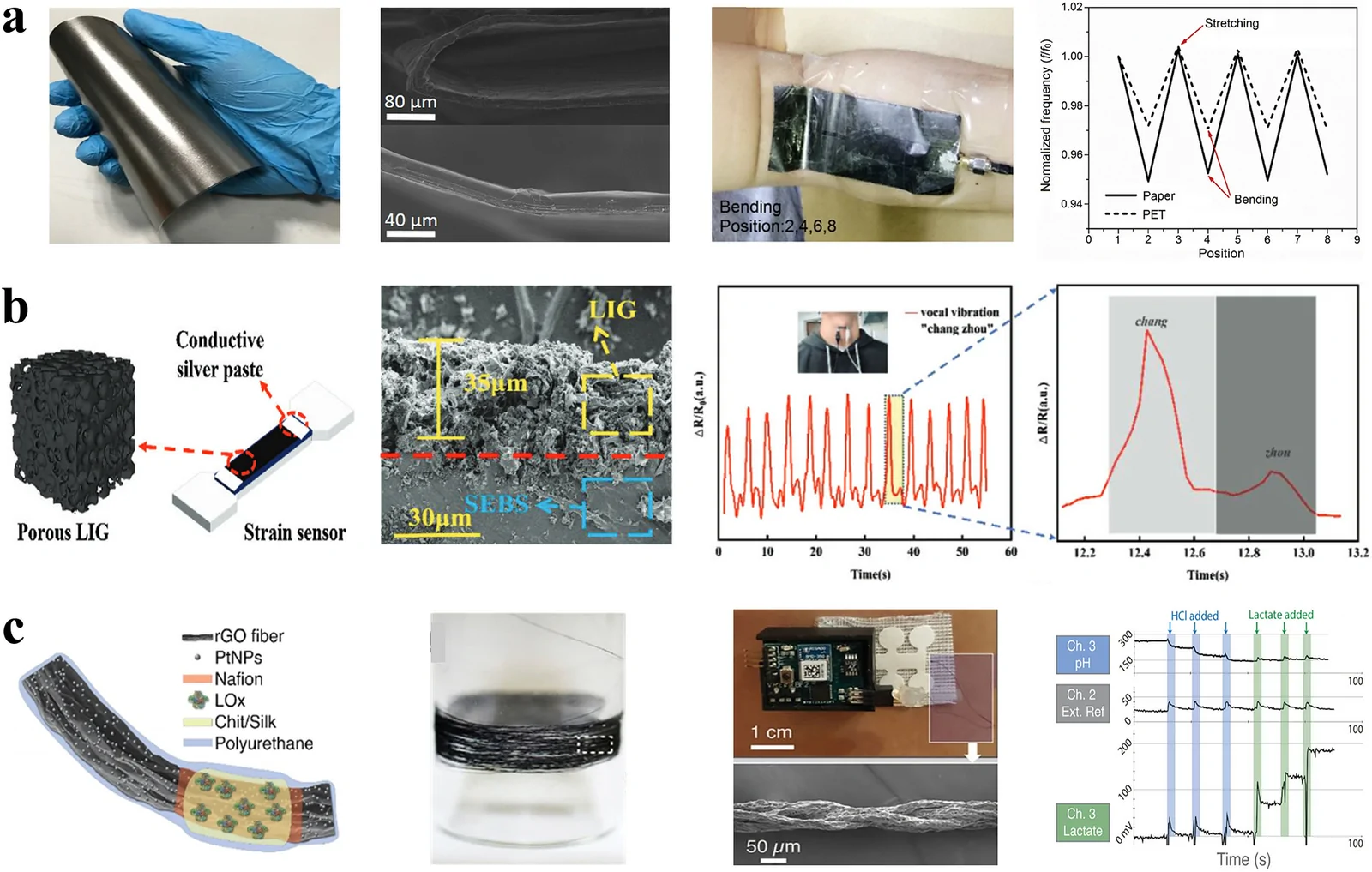
**a** Wireless strain sensing for human joint bending based on a flexible multilayer graphene film antenna [171]. Reproduced with permission. Copyright 2018, Science Bulletin. **b** Real-time wireless monitoring of physiological signals using an embedded LIG flexible sensor [172]. Reproduced with permission. Copyright 2024, Advanced Materials Technologies. **c** Wireless detection of sweat lactate and pH using rGO-based electrochemical sensing yarns [173]. Reproduced with permission. Copyright 2024, Communications Materials

Recent progress in graphene-based wireless sensors demonstrates advancements from physical strain monitoring to biochemical analysis. For physical sensing, Tang et al. [171] (Fig. 16a) developed a battery-free, passive wireless strain sensor using a flexible multilayer graphene film antenna. By translating structural deformation into resonant frequency shifts, it effectively monitored large-scale joint movements. To enhance complexity and range, Liu et al. [172] (Fig. 16b) embedded LIG into elastomers alongside a Bluetooth module, enabling real-time, active monitoring of multiscale movements from delicate pulse waves to large-joint deformations. For biochemical sensing, Napier et al. [173] (Fig. 16c) created multifunctional rGO electrochemical sensing yarns, integrating lactate and pH detection into a single yarn that can be woven into textiles for real-time monitoring. Additionally, Wang et al. [174] described a breakthrough sensor (NutriTrek) using laser-engraved graphene (LEG) with molecularly imprinted polymers for continuous, non-invasive monitoring of metabolites under resting conditions, bypassing the need for exercise.

Despite these capabilities, the long-term wireless reliability of graphene-based sensors faces practical limitations. From a materials and manufacturing perspective, these structural vulnerabilities primarily manifest as severe signal distortion and electrical noise during continuous healthcare monitoring. Specifically, interfacial stress mismatch and strain-induced crack propagation under dynamic stretching frequently lead to unpredictable resistance fluctuations and motion artifacts [175]. Additionally, the complexity of transfer fabrication processes and susceptibility to environmental carrier scattering further exacerbate baseline noise and signal instability, which remain significant hurdles for large-scale integration [176]. To advance the durability and scalability of these wearable systems, future research should focus on enhancing cyclic mechanical stability by constructing hierarchical conductive pathways or composite transition layers to mitigate motion-induced noise. Furthermore, transitioning toward scalable manufacturing techniques such as printable or laser direct-write processes will be critical for the seamless, low-noise co-fabrication of active graphene materials with integrated wireless circuits.

#### Carbon Nanotubes

Carbon nanotubes (CNTs), including single-walled (SWCNTs) and multi-walled (MWCNTs) variants, possess high charge carrier mobility and excellent mechanical robustness [177–180]. In polymer-based flexible wireless sensors, their 1D geometry enables the formation of highly efficient percolating conductive networks even at low loading fractions. Rather than functioning as intrinsic system-level amplifiers, the primary advantage of CNTs lies in their structural stability. They maintain reliable mechanical–electrical responses under repeated deformations, which helps suppress baseline drift and ensures stable signal acquisition for continuous monitoring [181].

**Fig. 17:**
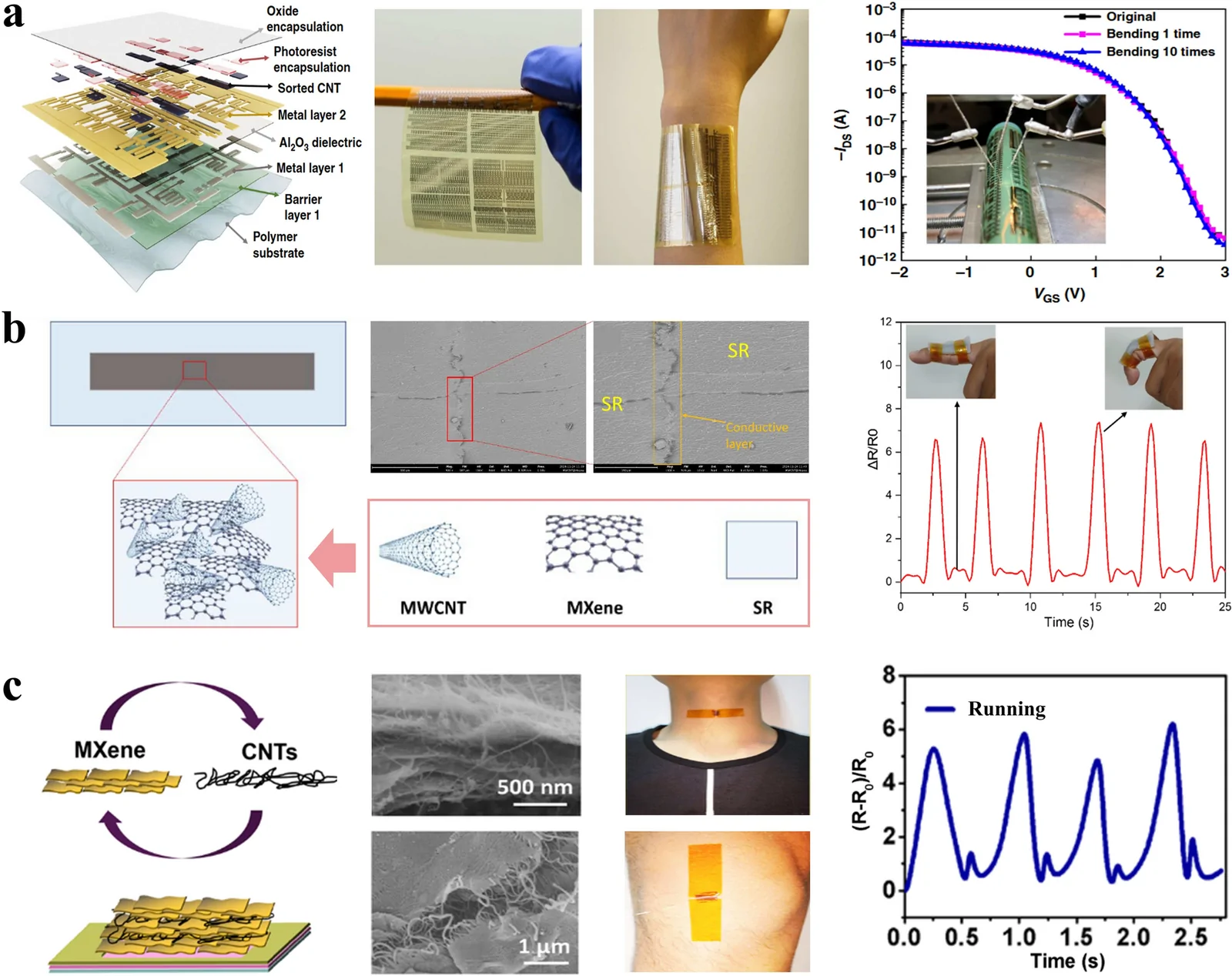
**a** Flexible thin-film transistor circuit based on substantial-purity semiconducting carbon nanotubes for skin-conformal wireless physiological signal acquisition [182]. Reproduced with permission. Copyright 2019, Nature Communications. **b** Flexible strain sensor based on an MWCNT@MXene conductive network for muscle activity and wearable health monitoring [183]. Reproduced with permission. Copyright 2025, Scientific Reports. **c** High-stretchability flexible strain sensor based on a CNT/MXene composite structure [184]. Reproduced with permission. Copyright 2018, ACS Nano

Recent integrations of CNTs into wearable systems highlight their utility in acquiring physiological data for wireless transmission. For instance, Lei et al. [182] (Fig. 17a) developed flexible thin-film transistor (TFT) circuits utilizing ultrahigh-purity semiconducting CNTs. This platform allowed for the conformal acquisition and wireless transmission of multidimensional signals, including heart rate and electromyography. To further enhance sensing capabilities, researchers frequently composite CNTs with 2D materials. Hakim et al. [183] (Fig. 17b) fabricated a flexible strain sensor using an MWCNT@MXene conductive network designed for low-power motion analysis and rehabilitation tracking. Similarly, Cai et al. [184] (Fig. 17c) engineered a highly stretchable CNT/MXene composite sensor capable of reliably capturing everything from subtle muscle contractions to large limb movements for wireless wearable systems.

Despite these robust sensing capabilities, practical applications of CNT-based flexible sensors face notable durability challenges. Weak van der Waals interfacial interactions can result in contact resistance drift, particularly in humid or sweaty conditions [185, 186]. Furthermore, prolonged cyclic deformation frequently induces irreversible microcracking within the polymer matrix, leading to baseline drift and signal hysteresis over long-term use [187]. To ensure the signal reliability required for continuous wireless transmission, future material designs must strengthen interfacial bonding through techniques like plasma treatment or molecular grafting. Additionally, the introduction of dynamic reversible bonds (such as hydrogen bonds or metal–ligand coordination) could create adaptive, reconfigurable conductive networks capable of mitigating performance degradation under continuous cyclic loading.

Table 6 summarizes representative studies of carbon-based materials in polymer-based flexible wireless sensors in recent years. It systematically compares fabrication methods, key performance metrics, primary advantages and limitations, and typical applications in health monitoring, providing a reference for material design and device performance optimization.

**Table 6:** Summary of representative polymer-based flexible wireless sensors based on carbon-based materials

Material System	Fabrication Method	Key Performance Metrics	Advantages / Limitations	Health Monitoring Applications	Refs
Graphene and Derivatives					
LIG/PI	CO_2_ laser induction (12.4 W, 105 mm s^−1^, 3 scans)	GF ≈ 41.4; strain 1%–8%; response/recovery 150/100 ms; > 2000s stability	High sensitivity, low detection limit, flexible; prone to cracking	Fine motor movement, pulse, eye motion	[188]
LIG/PI/PDMS	CO_2_ laser induction (12%, 16 kHz, 200 mm s^−1^)	GF = 30; strain 0–15%	High stretchability, low cost, one-step fabrication; non-linear GF	Pulse, speech, joint monitoring	[189]
Graphene/PDMS	Spin coating transfer (sandpaper template, NH_4_HCO_3_ foaming)	321 kPa^−1^; 0.01–1000 kPa; response 29 ms; > 5000 cycles	Substantial sensitivity, wide range; requires dispersion optimization	Pulse, joint motion	[190]
GNPs/PDMS	Composite casting (sandwich structure)	GF = 6.3; strain 97%	High sensitivity, flexible; packaging size effect	Electronic skin, body monitoring	[191]
Graphene/stretchable yarn	Layer-by-layer assembly	Strain 150%; tunable sensitivity	High stretchability, multiscale detection; CVD complex	Joint, speech, respiration monitoring	[192]
rGO/Fe NWs/PDMS	Hummers’ method + magnetic self-assembly	3.24–6.44 kPa^−1^; response 18 ms; > 6000 cycles	High sensitivity, wide range, low hysteresis; deposition needs optimization	Pulse, swallowing, respiration, motion monitoring	[193]
rGO/Silicone encapsulant	Electrochemical exfoliation + drop-casting	GF = 4100; strain 116%; > 4550 cycles	Substantial elasticity, durable, low-cost; resistance saturation at high strain	Walking, running, limb motion	[194]
Carbon Nanotubes					
CNT/Ecoflex	Three-roll dispersion + casting	GF 10^4^–10^5^; strain up to 300%; response 600 ms; 2000 cycles	Highly flexible, substantial GF; noticeable hysteresis	Respiration monitoring (wireless)	[195]
MWCNT/PCU-PTMGU	CVD growth + dry spinning + elastomer impregnation	GF > 10; strain up to 200%; response < 15 ms	High sensitivity, fast response; high initial resistance	Finger/body motion monitoring	[196]
SWCNT/Cotton fabric	Dipping and drying	GF 6.0/1.2; strain 150%; > 50,000 cycles	Breathable, washable, highly stable; hysteresis at high strain	Multi-directional human motion	[197]
MWCNT/PDMS	Ultrasonic dispersion + curing	GF = 37; strain 2%-10%; high cycle stability	High sensitivity, repeatable; electrode adhesion issues	Elbow/finger bending	[198]

### Metal-Based Fillers in Polymer Matrices

Metal-based materials are essential for polymer-based flexible wireless sensors due to their superior electrical conductivity, stability, and substrate compatibility compared to carbon or polymers. Acting as both high-precision sensing and communication interfaces, their low-resistance properties enable optimal RF impedance matching and efficient energy coupling. By minimizing signal attenuation and reflection losses, these metals considerably enhance wireless power transfer, data transmission, and antenna/coil integration. This makes them indispensable for NFC, RFID, and low-power wireless architectures frequently utilized in continuous health monitoring applications.

#### Solid-State Metal-Based Materials

Solid-state metals, such as Ag, Au, and Cu films, are highly conductive and chemically stable materials frequently utilized in flexible sensing [199, 200]. In wearable devices, their primary function is to provide ultra-low resistance pathways, which are essential for high-fidelity signal acquisition and efficient wireless transmission [201, 202]. To overcome their intrinsic rigidity, these metal films often adopt engineered architectures such as microcracking or wrinkling to accommodate mechanical deformation. This allows them to maintain stable electrical continuity during body movement without acting as complex, system-level dynamic filters.

**Fig. 18:**
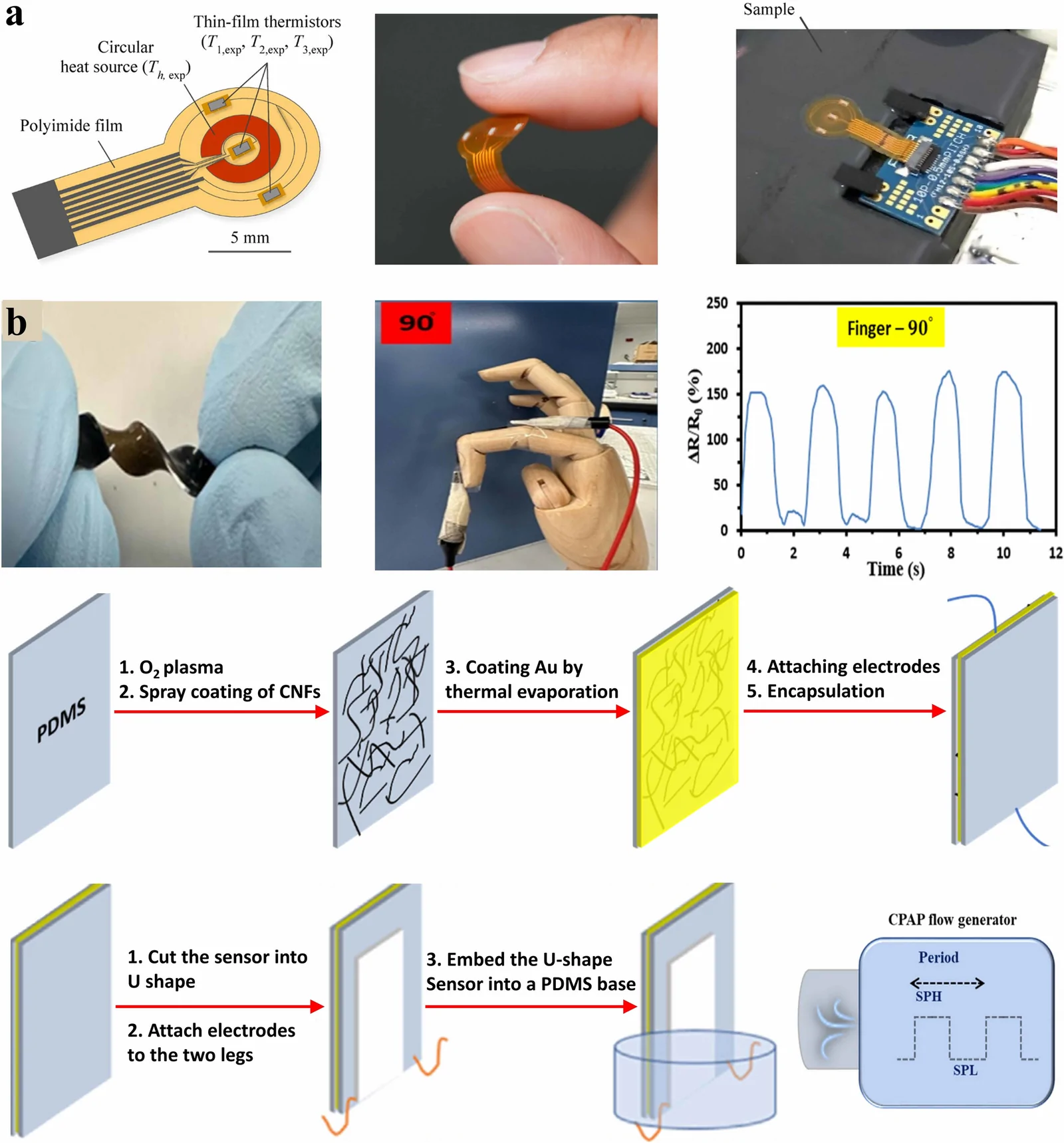
**a** Flexible thermal sensor based on Au/Ni solid-state metal films for non-invasive monitoring of tissue thermal properties and body temperature [203]. Reproduced with permission. Copyright 2025, Scientific Reports. **b** Highly stretchable piezoresistive flexible strain sensor based on solid-state Au films for motion and respiration monitoring [204]. Reproduced with permission. Copyright 2023, Smart Materials in Manufacturing

Recent applications leverage solid-state metals primarily for precise thermal and mechanical health monitoring. For thermal sensing, Okabe et al. [203] (Fig. 18a) developed a flexible sensor utilizing Au/Ni solid-state metal films. By integrating thin-film heaters and thermistors, the device provides non-invasive, continuous tracking of tissue thermal properties and body temperature. For motion and respiration monitoring, Vahdani et al. [204] (Fig. 18b) reported a highly stretchable piezoresistive strain sensor based on thermally evaporated Au thin films. By incorporating carbon nanofibers to control short-range microcrack propagation, the sensor achieves significant stretchability and sensitivity, enabling the real-time capture of everything from subtle airflow changes to large joint movements.

Despite their superior conductivity, solid-state metal films face inherent mechanical limitations in flexible applications. Continuous cyclic deformation typically leads to mechanical fatigue, uncontrolled microcrack propagation, and subsequent baseline signal drift. Furthermore, maintaining reliable interfacial adhesion between rigid metallic layers and soft polymer substrates remains a critical challenge, especially during prolonged use in humid or sweat-rich environments [205, 206]. To address these mechanical mismatch issues, future sensor designs should emphasize multilayer metal/polymer composites or heterostructured architectures, which can generate adaptive microcrack networks to improve cyclic durability. Additionally, implementing targeted interface engineering strategies such as surface passivation or plasma treatment will be vital to reinforce interfacial adhesion and ensure sustained reliability during dynamic on-body monitoring.

#### Liquid Metal-Based Materials

Gallium-based liquid metals, such as eutectic gallium-indium (EGaIn), offer intrinsic fluidity that allows their conductive pathways to dynamically reconfigure under severe deformation [207, 208]. In polymer-based flexible wireless sensors, this unique electromechanical adaptability minimizes resistance hysteresis and prevents structural fatigue during cyclic stretching. Rather than dynamically managing system-level RF impedance or processing data, their primary contribution is providing an ultra-stretchable, continuous electrical foundation that resists mechanical failure, ensuring reliable signal acquisition during multiscale bodily movements.

**Fig. 19:**
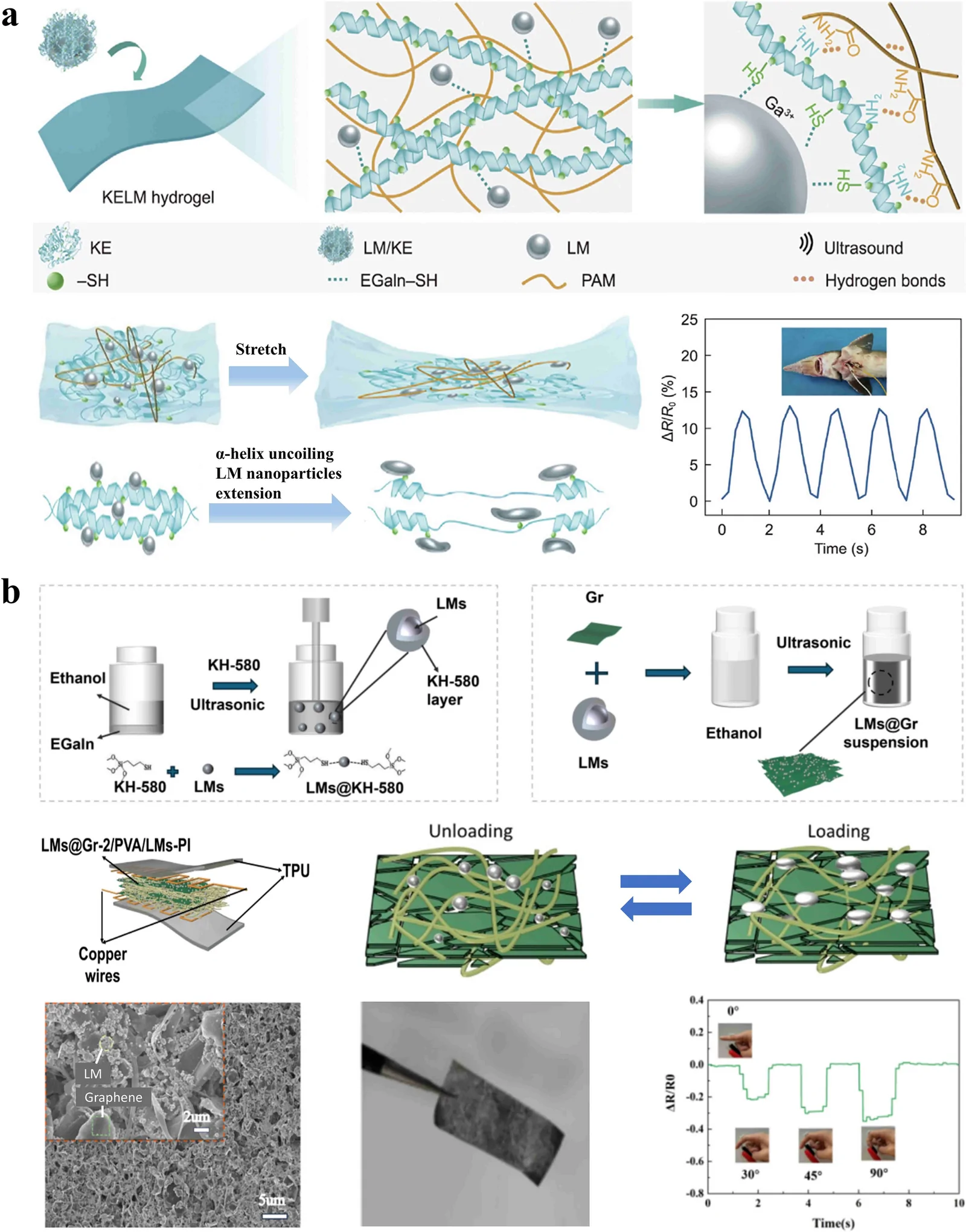
**a** Flexible sensor based on keratin-liquid metal composite hydrogel for multimodal physiological signal monitoring [209]. Reproduced with permission. Copyright 2025, Engineering. **b** Liquid metal flexible pressure sensor based on EGaIn-graphene composite conductive nanofibers for motion monitoring [210]. Reproduced with permission. Copyright 2025, Materials and Design

The operational capabilities of liquid metal sensors are intrinsically dictated by their structural matrix. Emphasizing conformability, Wu et al. [209] (Fig. 19a) developed a highly stretchable, tissue-like hydrogel by chemically anchoring EGaIn within a natural keratin network. This highly biocompatible system excels in the non-invasive monitoring of delicate physiological signals. Alternatively, prioritizing robust durability, Gong et al. [210] (Fig. 19b) engineered a flexible pressure sensor by integrating EGaIn and graphene into an electrospun nanofiber network. This physically entangled dual-conductive network trades extreme stretchability for superior fatigue resistance, making it better equipped to handle large-scale biomechanical deformations like continuous joint tracking.

Despite their unmatched elasticity, the practical integration of liquid metals is hindered by their high surface tension and fluidity. Key challenges include internal droplet migration under localized pressure, difficulties in high-resolution fine-patterning, and complex encapsulation requirements to prevent fluid leakage [211, 212]. From a materials and manufacturing perspective, these structural instabilities inevitably translate into severe signal distortion and electrical noise in healthcare applications. Specifically, the redistribution of liquid metal and imperfect encapsulation under dynamic body movements cause unpredictable resistance fluctuations and motion artifacts, severely compromising continuous monitoring. To overcome encapsulation complexity and mitigate these noise-inducing factors, future strategies should emphasize interface engineering, such as utilizing polymer grafting or hydrogen bonds to stabilize EGaIn within matrices. Furthermore, developing composite networks with solid conductive nanomaterials (e.g., CNTs) and combining them with advanced microfluidic channel designs will be critical for regulating droplet distribution, enhancing fatigue resistance, and improving scalable fabrication precision.

Table 7 summarizes representative recent studies on polymer-based flexible wireless sensors based on metallic materials. It systematically compares the fabrication methods and structures of different material systems, key performance metrics, advantages and limitations, and typical applications in health monitoring, providing a reference for subsequent material selection and device optimization.

**Table 7:** Summary of representative polymer-based flexible wireless sensors based on metallic materials

Material system	Fabrication and structural design	Key performance metrics	Material- and process-specific advantages and limitations	Health monitoring application	Refs
Solid metals					
Au(Cr)/paper substrate + Ta_2_O_5_ (potentiometric, pH)	Cr/Au conductive layers and a Ta_2_O_5_ sensing layer were sequentially deposited on a paper-based flexible substrate by magnetron sputtering; the fibrous paper formed a micrometer-scale porous electrode network, integrated with a miniaturized BLE-based potentiometric readout module	pH sensitivity ≈ 41 mV/pH; stable bending radius of 6.5–25 mm; operational stability for weeks	Advantages: Dense sputtered Au film with excellent chemical stability; porous paper enhances sweat contact; Limitations: Ta_2_O_5_-based potentiometric response does not reach the theoretical Nernst slope	Sweat pH monitoring with detectable pH variation of ~ 0.1–0.2	[213]
Au/CNTs-PDMS (resistive, strain)	A CNT conductive film was transferred onto a pre-stretched PDMS substrate followed by Au deposition; release induced periodic wrinkled conductive structures, coupled with a wireless readout module	GF ≈ 70; response time < 60 ms; durability > 10,000 cycles	Advantages: Au maintains continuous conduction in strain-concentrated wrinkle regions, amplifying tunneling effects; Limitations: Continuous Au film restricts ultimate stretchability and increases cost	Finger bending detection with strain range of 0–20%, enabling discrimination of bending angles	[214]
Au nanolayer/MWCNTs + FLG + ionic liquid (resistive, strain)	A carbon-based composite layer was patterned by screen printing, followed by sputtering of an ultra-thin Au nanolayer to induce controlled cracks; serpentine electrodes and a passive NFC chip were integrated	GF_max_ ≈ 2416; strain range 0–80%; wireless reading distance ≈ 3 cm	Advantages: Positive temperature coefficient of cracked Au compensates the negative temperature coefficient of carbon materials, achieving intrinsic thermal self-compensation; Limitations: Performance strongly depends on precise control of Au nanolayer thickness	Knee ligament strain monitoring with high-resolution tracking of dynamic strain amplitude and rate	[215]
Ag@Au nanowires/Au hollow nanowires on SEBS (resistive, strain)	Core–shell and hollow Au-based nanowires were synthesized via solution processes and monolithically patterned on SEBS substrates, together with integrated wireless modules	ΔR/R₀ ≈ 773 at 100% strain; stretchability > 2000 cycles	Advantages: Au shell suppresses Ag oxidation, while hollow structures accommodate large deformation; Limitations: Electrical uniformity is sensitive to nanowire geometry and wall thickness	Finger and neck motion monitoring with strain range up to 100% and real-time wireless signal output	[216]
Au/Ag/Cu nanowires or nanoparticles on flexible polymers (resistive/piezoresistive)	Metal nanowire or nanoparticle networks were formed by printing or transfer processes on flexible polymer substrates with serpentine or mesh layouts, partially integrated with BLE or NFC modules	GF from 50 to several hundred; strain range 50%–200%; ms-level response	Advantages: High intrinsic conductivity combined with crack/tunneling mechanisms yields high sensitivity; Limitations: Cu is prone to oxidation, while Au/Ag require passivation for long-term stability	Pulse monitoring (60–100 bpm), respiration-induced deformation amplitude recognition	[217]
Liquid metals					
Ni-GaIn on silicone elastomer (resistive, multimodal)	Laser-patterned masks and roll-coating were used to form serpentine Ni-GaIn conductive paths on silicone substrates, integrated with a passive NFC tag	Strain range 0–50%; resistance ≈ 1.2 Ω; sampling rate 24 Hz	Advantages: Ni particles enhance wetting and adhesion of GaIn, preventing electrical discontinuity; Limitations: Excessive Ni content degrades conductivity continuity	Finger joint bending monitoring with strain amplitude of 0–30% and real-time waveform output	[218]
GaInSn in PDMS microchannels (resistive, strain/pressure)	GaInSn liquid metal was selectively filled into photolithographically defined PDMS microchannels, followed by multilayer encapsulation and NFC coil integration for wireless readout	GF ≈ 2; stretchability 0–30%; durability > 10,000 cycles	Advantages: Liquid metal maintains continuous conductivity under large deformation; Limitations: Flow confinement in microchannels introduces hysteresis under compression	Wrist flexion monitoring with continuous wireless strain measurement (0–30%)	[219]
Liquid metal putty (inductive, strain)	Planar inductive coils were fabricated and filled with liquid metal putty to form highly stretchable inductive sensing units, read out via inductive wireless coupling	Strain range 0–500%; resolution 0.05%; durability > 5000 cycles	Advantages: Inductive sensing avoids resistance drift and supports extreme deformation; Limitations: High requirements for material uniformity and encapsulation	Respiration monitoring with chest deformation amplitude of ~ 5%–50%	[220]

### Functional Polymeric Systems and Substrates

In wireless health monitoring systems, polymers utilize their tunable molecular structures and flexible mechanical properties to regulate device electromechanical responses. Functioning seamlessly as sensitive layers, functional interfaces, and flexible substrates, they govern the system’s skin adaptability, signal sensitivity, and environmental stability. By ensuring stable signal acquisition under dynamic physiological conditions, polymers support the efficient integration of the sensing layer with wireless modules, ultimately driving robust overall system performance.

#### Hydrogel-Based Materials

Hydrogels are characterized by their compliant mechanical properties, high water content, and excellent biocompatibility. They are capable of closely conforming to skin and tissue surfaces. This enables them to provide a stable interface for acquiring low-power wireless signals and monitoring physiological parameters continuously [221, 222]. Compared with conventional conductive polymers or inorganic materials, hydrogels achieve enhanced skin adaptability and possess ionic conductivity, electrochemical stability and tunable structural characteristics. These advantages enable them to deliver exceptional performance in multimodal signal acquisition, including heart rate, pulse, joint motion, and tactile sensing [223–226].

##### Naturally Derived Hydrogel Materials

Naturally derived hydrogels, formulated from biopolymers like alginate, silk fibroin, or cellulose, offer exceptional biocompatibility and tissue-like compliance. Their intrinsic ionic conductivity and hydrophilic networks allow them to intimately conform to the skin. In wireless sensing platforms, their core function is to establish a stable physical and electrical tissue–sensor interface, effectively minimizing contact impedance rather than acting as complex biologically selective permeable membranes.

**Fig. 20:**
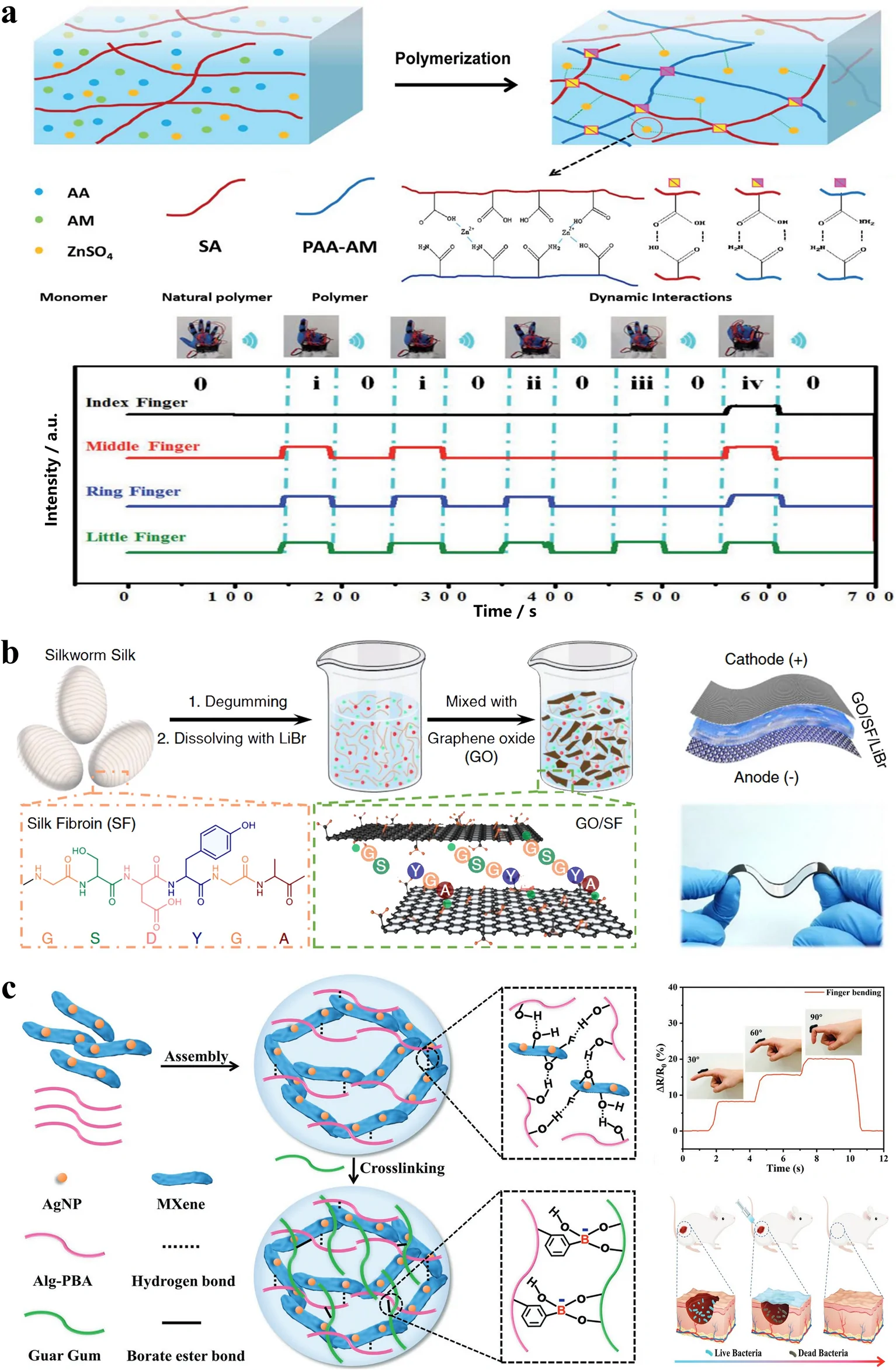
**a** Naturally derived hydrogel based on a sodium alginate and ZnSO_4_ double network, serving as a highly stretchable, self-healing ionic conductive layer for wireless flexible strain sensors to monitor motion [227]. Reproduced with permission. Copyright 2020, Journal of Materials Chemistry A. **b** Self-powered humidity sensor based on silk fibroin and graphene oxide hydrogel electrolyte for real-time respiration monitoring [228]. Reproduced with permission. Copyright 2022, Nature Communications. **c** Self-healing, antibacterial flexible hydrogel sensor based on a guar gum/phenylboronic acid-grafted sodium alginate double network with MXene nanosheet conductive structure [229]. Reproduced with permission. Copyright 2022, Advanced Functional Materials

Recent advancements highlight the versatility of these natural matrices. For physical monitoring, Huang et al. [227] (Fig. 20a) developed a highly stretchable and self-healing double-network hydrogel using sodium alginate and ZnSO_4_, ensuring reliable ionic conduction for macroscopic joint motion tracking. Transitioning toward energy-independent biochemical sensing, Li et al. [228] (Fig. 20b) engineered a self-powered humidity sensor based on a silk fibroin and graphene oxide hydrogel to capture real-time respiratory signals. Furthermore, Li et al. [229] (Fig. 20c) demonstrated a multimodal approach by integrating MXene nanosheets into a guar gum/alginate double network. This antibacterial, self-healing matrix provided sensitive responsiveness to both human motion and electrophysiological signals (e.g., ECG, EMG). Notably, during EMG signal detection, the MXene hydrogel sensor demonstrated superior signal acquisition quality compared to commercial hydrogel electrodes, achieving a distinctively higher SNR of 17.80 dB versus the commercial standard’s 10.17 dB.

The practical deployment of natural hydrogels in long-term wearable systems is intrinsically limited by their high water content. Prolonged operation inevitably leads to water evaporation and sweat-induced ionic imbalances, resulting in structural stiffening, interfacial delamination, and severe baseline impedance drift [230]. To overcome these stability issues, future material designs must focus on anti-drying strategies, such as introducing hygroscopic salts or transitioning to organohydrogel systems. Additionally, incorporating dynamic coordination bonds and optimizing conductive nanofiller networks will be crucial to mitigating electrical degradation and extending the reliable operational lifespan of these sensors.

##### Synthetic Polymer-Based Hydrogels

Synthetic polymer-based hydrogels, such as polyurethane (PU) or polyacrylamide (PAAm), offer highly programmable mechanical and electrical properties. By incorporating ionic liquids, salts, or conductive nanomaterials, they form robust conductive networks [231–233]. Their primary contribution to wireless health monitoring is their highly tunable elasticity and environmental resilience, which ensure consistent signal coupling during dynamic bodily movements.

**Fig. 21:**
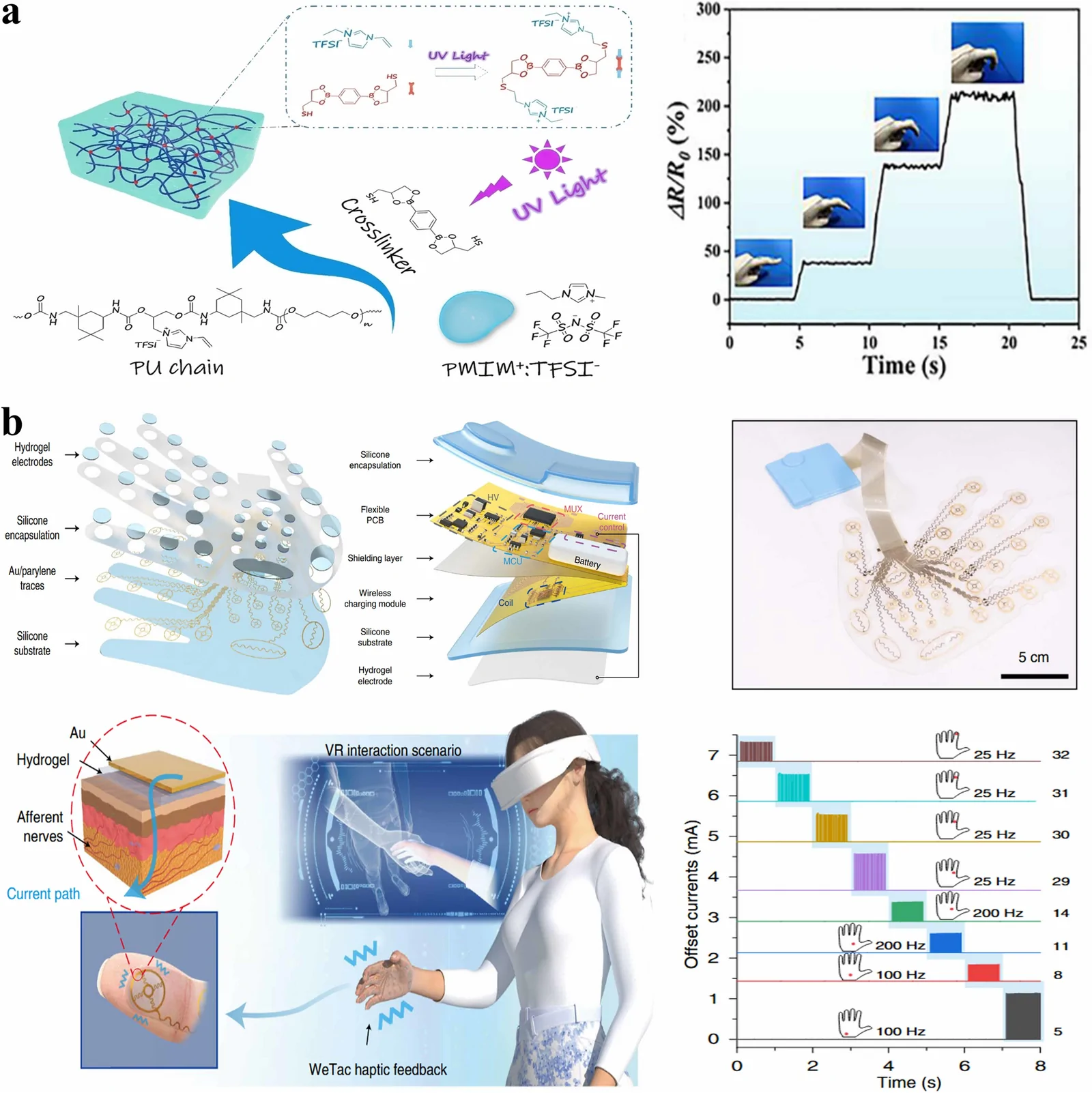
**a** Self-healing, highly conductive ionogel based on a polyurethane/ionic liquid composite network for monitoring human motion and physiological signals [234]. Reproduced with permission. Copyright 2021, Chemical Engineering Journal. **b** Ultraflexible, low-impedance PAAm-based ion hydrogel electrode for skin-integrated tactile interface applications [235]. Reproduced with permission. Copyright 2022, Nature Machine Intelligence

Synthetic hydrogels are frequently engineered to balance mechanical toughness with electrical stability. Xu et al. [234] (Fig. 21a) developed a highly resilient PU ionogel by incorporating ionic liquids into a polyurethane network. This design decoupled mechanical stress from electrical conduction, creating a highly durable and self-healing strain sensor for continuous human activity monitoring. Prioritizing ultrasoft conformability for tactile applications, Yao et al. [235] (Fig. 21b) engineered the WeTac system using a low-modulus PAAm hydrogel. This ultra-compliant matrix acts as a low-impedance epidermal patch, maintaining stable skin contact to facilitate efficient current conduction for wireless tactile stimulation and feedback. Beyond epidermal tactile feedback, synthetic hydrogels play an indispensable role in closed-loop wound environment monitoring. Highly conductive and mechanically robust synthetic networks, such as PEDOT:PSS-based hydrogels, can construct skin-interfacing electrodes that seamlessly bridge the gap between dynamic human tissues and rigid RF electronics. These integrated platforms facilitate the wireless, battery-free tracking of dynamic wound environments, including localized temperature and tissue impedance. This enables real-time clinical interventions and accelerated tissue regeneration [164].

Despite their tunable properties, synthetic hydrogels and ionogels face significant operational hurdles. Their reliance on ion migration means that long-term cyclic loading often induces ion redistribution and signal hysteresis [236–239]. Furthermore, complex and multi-step polymerization processes currently limit their scalable manufacturing. Future research should focus on simplifying synthesis routes for scalable fabrication. From a materials perspective, integrating dynamic reversible crosslinks (such as metal–ligand or boronate ester bonds) into the polymer backbone will be essential to further decouple mechanical deformation from resistance changes, thereby minimizing signal hysteresis and enhancing long-term cycling stability.

#### Conductive and Functional Polymers

Conductive polymers (e.g., PEDOT:PSS, PANI) and functional polymers (e.g., PVDF) provide versatile electrical and electromechanical properties for flexible sensors. Rather than acting as complex active electronic front-ends or autonomous system nodes, their core value lies in their intrinsic material capabilities. Conductive polymers offer exceptionally low interfacial impedance, which is critical for capturing high-fidelity bioelectrical signals (such as ECG and EMG) at the tissue interface. Conversely, functional polymers like PVDF utilize piezoelectric properties to directly transduce mechanical deformations into electrical signals, establishing a physical foundation for self-powered sensing [240–245].

**Fig. 22:**
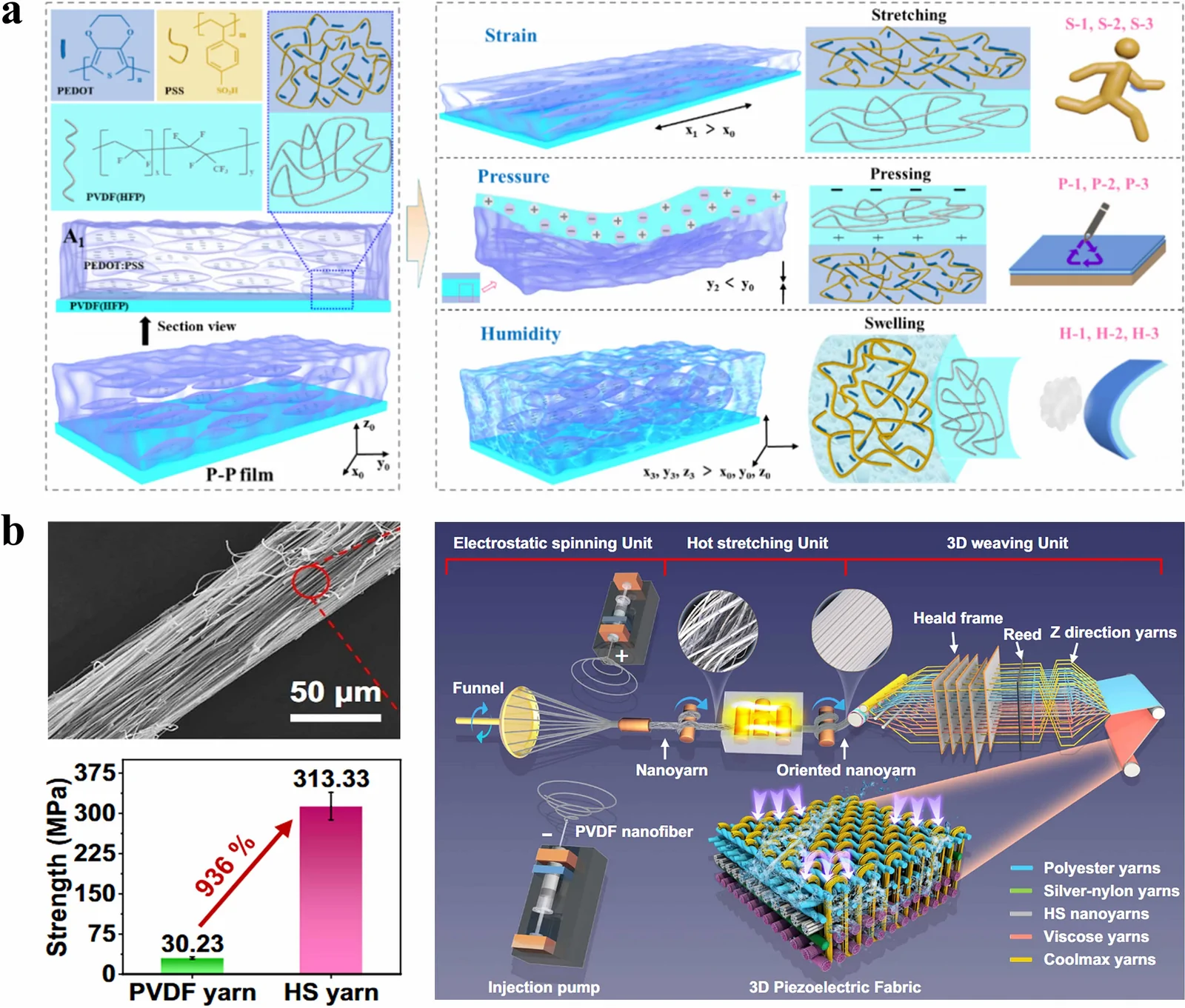
**a** Flexible sensor based on a PEDOT:PSS/PVDF(HFP) bilayer conductive-piezoelectric composite film, featuring high conductivity, piezoelectric output, and rapid humidity response [246]. Reproduced with permission. Copyright 2024, Chemical Engineering Journal. **b** Three-dimensional piezoelectric fabric sensor based on PVDF functional polymer, offering high mechanical strength, sweat-enhanced piezoelectric response, and real-time physiological signal monitoring [247]. Reproduced with permission. Copyright 2024, Nature Communications

Recent device designs focus on structural optimization to enhance both signal coupling and wearer comfort. For multimodal sensing, Wu et al. [246] (Fig. 22a) developed a bilayer film combining conductive PEDOT:PSS with piezoelectric PVDF(HFP). This composite structure effectively captures simultaneous strain, pressure, and humidity stimuli, providing a robust material basis for kinematic and physiological monitoring. Addressing the need for long-term wearability, Fan et al. [247] (Fig. 22b) engineered a three-dimensional piezoelectric fabric (3DPF) utilizing electrospun PVDF nanofibers. This 3D architecture prioritizes encapsulation-free breathability, facilitating sweat transport while simultaneously harnessing that moisture to enhance the piezoelectric response for cardiovascular and motion tracking.

Despite their distinct advantages, these polymers face specific environmental and mechanical limitations during continuous wear. Conductive polymers like PEDOT:PSS are highly susceptible to dehydration and phase separation under fluctuating humidity, which inevitably causes impedance drift. Similarly, PANI can experience volumetric expansion during repeated doping cycles, leading to response hysteresis. For functional polymers like PVDF, efficient mechanical-to-electrical transduction relies strictly on a high *β*-phase crystalline content, which is difficult to maintain without stringent thermal or electrical poling processes. In summary, from a materials and manufacturing perspective, these inherent structural instabilities and processing inconsistencies directly contribute to severe baseline wandering, dynamic electrical noise, and signal distortion, fundamentally degrading signal fidelity in healthcare applications. To address these limitations, future material engineering efforts should prioritize interface engineering strategies to stabilize moisture distribution within conductive polymer networks. Such stabilization can potentially be realized through the incorporation of heterogeneous carbon or inorganic nanomaterials. For piezoelectric polymers, the advancement of scalable manufacturing technologies, including controlled nanofiber electrospinning and multilayer lamination, will be critical to maximizing polarization and energy conversion efficiency while maintaining structural flexibility.

#### General Flexible Substrates

Flexible substrates ranging from elastomers (e.g., PDMS, PU) and thermoplastics (e.g., PI, PET) to paper-based materials serve as the foundational structural anchors for wireless sensing systems [248, 249]. Rather than dynamically regulating energy or data transmission, their core function is to provide necessary mechanical support and conformability. Elastomers offer low moduli to match skin mechanics, thermoplastics provide dimensional stability for high-frequency antenna geometries, and paper materials offer inherent porosity for natural biofluid wicking. Together, they mitigate mechanical mismatch and stabilize the electrode–skin interface.

**Fig. 23:**
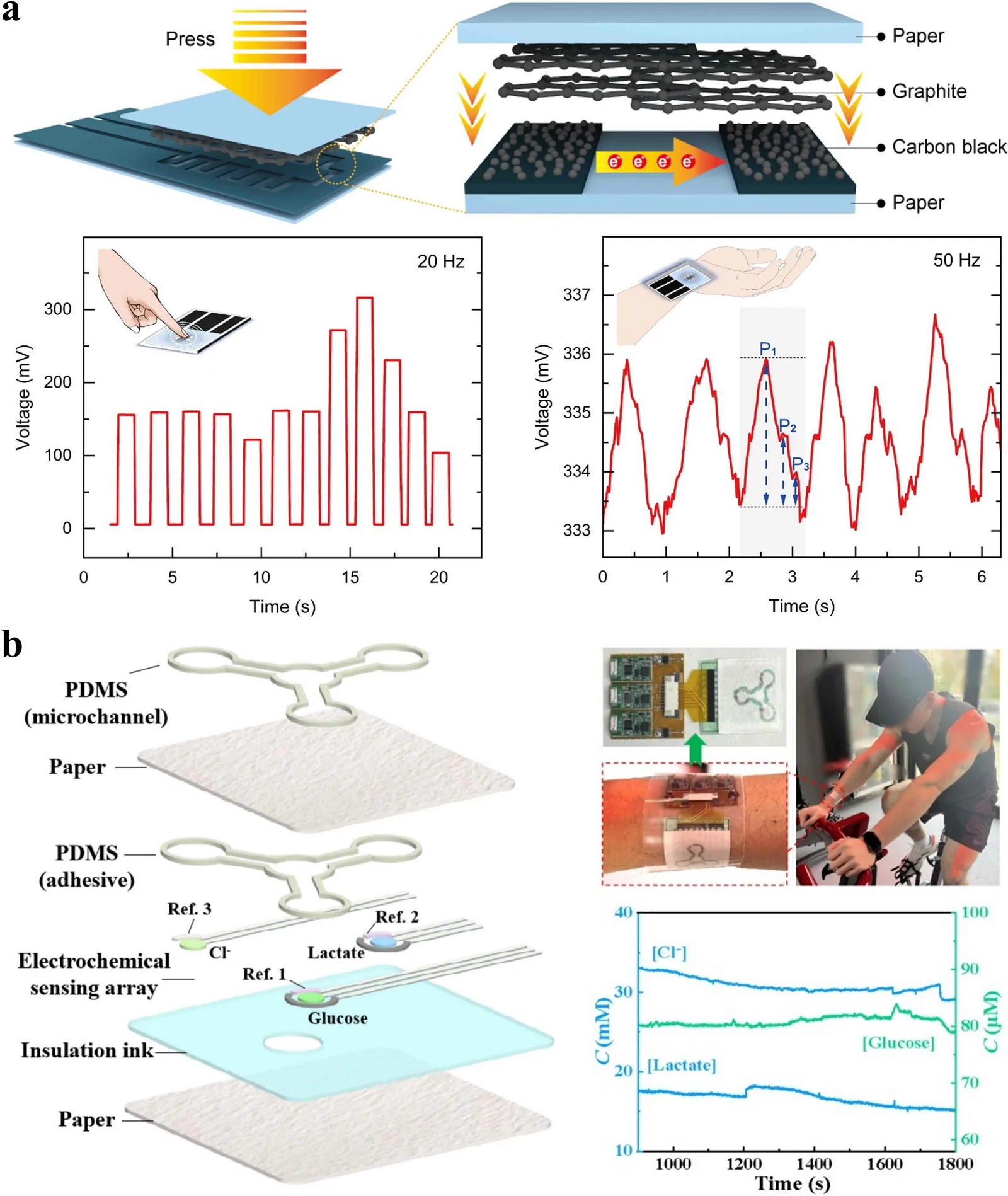
**a** Schematic illustration of the layered structure of the paper-based sensor under applied pressure; sensor signal response curve during finger pressing at a 20-Hz sampling frequency; and dynamic variation curve of the human pulse signal at a 50-Hz sampling frequency [251]. Reproduced with permission. Copyright 2024, Chemical Engineering Journal. **b** Schematic diagram of the 3D paper-based microfluidic electrochemical sensor for sweat analysis; photograph of the worn device; and demonstration of the application scenario for real-time in situ on-body monitoring of human sweat during stationary cycling [252]. Reproduced with permission. Copyright 2025, Chemical Engineering Journal

Recent device designs select specific substrates based on targeted monitoring modalities. For electrophysiological tracking, Mishra et al. [250] utilized a low-modulus elastomeric film combined with PI to create a conformal periocular wearable for high-precision eye tracking. For biochemical and pressure sensing, paper has emerged as a highly versatile substrate due to its capillary action. Chen et al. [251] (Fig. 23a) leveraged the porous cellulose network of paper to develop an ultra-thin pressure sensor, facilitating rapid electrolyte diffusion for physical monitoring. Expanding on this fluidic capability, Mei et al. [252] (Fig. 23b) developed a 3D paper-based microfluidic sensor. The paper’s inherent wicking properties enabled the automatic, capillary-driven transport of sweat, allowing for the simultaneous, pump-free detection of multiple metabolites during exercise. Furthermore, Li et al. [253] ingeniously developed an advanced flexible photoelectrochemical sensor utilizing a flexible paper-based chip for the prehospital diagnosis of atherosclerosis. This inspiring work proposed a dual-electric-field-mediated photothermal-thermoelectric synergistic strategy to overcome thermally induced interfacial electron recombination, achieving highly sensitive detection of Lp-PLA_2_ in body fluids, which provides a highly promising portable solution for the early screening of severe cardiovascular diseases.

Despite their structural utility, practical applications of these substrates are restricted by inherent material trade-offs. Elastomeric substrates like PDMS suffer from poor air permeability, leading to sweat accumulation and subsequent signal degradation over long-term wear. Conversely, rigid thermoplastics and FPCBs are prone to interfacial delamination under repeated cyclic deformation [254, 255]. Furthermore, while paper-based materials excel in fluid manipulation, they are highly vulnerable to structural degradation, swelling, and performance drift when subjected to prolonged exposure to high humidity or bodily fluids. Future substrate engineering must prioritize hybrid architectures to address these bottlenecks. Promising strategies include embedding high-strength PI frameworks within soft PDMS matrices to balance elasticity with dimensional stability, and implementing micro-perforated or honeycomb structures to enhance breathability. For paper-based platforms, the application of advanced hydrophobic surface treatments such as methyltrichlorosilane (MTS) coating will be essential to prevent structural collapse and extend the reliable operational lifespan of the sensor in sweat-rich environments [256, 257].

Table 8 summarizes recent representative studies on polymer materials in the fabrication of polymer-based flexible wireless sensors, systematically comparing the fabrication methods, key performance metrics, main advantages and limitations, and typical applications in health monitoring. This provides a reference for subsequent material selection and device optimization.

**Table 8:** Summary of representative polymer-based flexible wireless sensors based on polymer materials

Material System	Fabrication Method and Wireless Integration	Key Performance Metrics	Advantages and Limitations	Health Monitoring Applications	Refs
GG/Alg-PBA hydrogel + AgNPs@MXene	Solution mixing with dynamic boronate ester crosslinking; wireless proof-of-concept demonstrated (motion signal transmission); human monitoring compatible with BLE	Strain range: ~ 166%; Self-healing efficiency: > 99% (elongation), ~ 95% (strength); Response: instantaneous conductivity recovery; Cycling stability: negligible degradation	Advantages: Dual self-healing, injectable/shear-thinning, antibacterial Limitations: Long-term wear induces excessive swelling and performance decay; ion/Ag^+^ leakage leads to gradual conductivity loss; moderate strength with potential fatigue under prolonged large-strain cycling	Joint motion: wrist/finger/elbow/knee bending angles; Pulse monitoring: P1/P2 peaks, AIx ≈ 0.50, upstroke time ~ 160 ms; ECG: clear P-QRS-T waves, HR ~ 75 bpm; EMG: grip-force discrimination (5–20 kg)	[229]
PAM/OGG double network + TA/Fe^3+^	One-pot in situ polymerization with Schiff-base crosslinking; wireless encrypted signal transmission demonstrated	GF: 11.82; Strain range: > 1060%; Response time: 55–60 ms; Hysteresis: η = 3.9%; Cycling stability: ≥ 2000 cycles	Advantages: Much lower hysteresis and higher linearity than most hydrogel sensors; skin-matched modulus for comfortable short-to-mid-term wear; self-healing (~ 96%) supports repeated attachment Limitations: Long-term dehydration requires encapsulation or rehydration; Fe^3+^-based ionic conduction susceptible to salt migration and signal drift under sweating	Joint motion: knee/elbow/wrist bending angles; Subtle motion: facial expression and swallowing detection; Handwriting/gesture recognition: trajectory and pattern discrimination; Wireless communication: Morse code and encrypted signals	[258]
PAM/tapioca starch double network (ionic)	Solution casting into thin films; fully integrated Bluetooth system for wireless respiration monitoring	Humidity sensitivity: 13,462.1%/RH; Strain range: ≥ 100%; Stability: good repeatability over multiple cycles	Advantages: Sensitivity enhanced under stretching (mechanically programmable); low-cost materials and simple processing with full wireless implementation Limitations: Pure ionic conduction leads to sensitivity degradation upon long-term dehydration; strong dependence on ambient humidity requires calibration	Respiration monitoring: breathing rate, interval, and apnea duration (SAS); Breathing pattern classification: normal, rapid, and apnea	[259]
PAM/SA anisotropic hydrogel	Confined pre-stretching followed by Ca^2+^ ionic crosslinking; wireless HMI demonstrated	Detection limit: 0.1%; Strain range: 1585%; Response time: 123 ms; Temperature tolerance: down to -20 °C	Advantages: Substantial stretchability suitable for large-amplitude motion; stable operation at low temperatures Limitations: Long-term dehydration remains a challenge; Ca^2+^-dependent ionic conduction may suffer ion migration in sweat-rich environments	Large-amplitude motion: limb and joint bending; Subtle deformation: < 1% strain detection; Wireless HMI: real-time motion-command transmission	[260]
PVA/sucrose colorimetric hydrogel	Solvent displacement method; smartphone imaging with app-based wireless readout	pH: 4–9; Glucose: 0–2 mM; Cl⁻: 0–100 mM; Ca^2+^: 0–16 mM	Advantages: No electrical circuitry required; simultaneous multi-analyte detection suitable for disposable POCT; good skin adhesion Limitations: Irreversible color change prevents continuous long-term monitoring; quantitative accuracy depends on lighting and image processing	Sweat analysis: pH, glucose, Cl⁻, and Ca^2+^ concentration ranges; Disease screening: diabetes and cystic fibrosis risk assessment	[261]
PVDF-TrFE/BaTiO_3_	Electrospinning with electrode encapsulation; integrated MCU with wireless transmission	Pressure sensitivity: 0.37 V kPa^−1^; Output voltage: up to 9.8 V; Frequency response: 2–7 Hz	Advantages: High sensitivity in low-frequency range matching physiological signals; no additional poling required Limitations: Sensitivity decreases at higher pressure levels; long-term durability under repeated bending of ultra-thin structures requires validation	Pulse monitoring: waveform and heart rate; Pressure mapping: spatial distribution via sensor arrays	[262]
PVDF + ZnO/rGO	Electrospinning; wireless fall-detection alarm system	Output voltage: ~ 11.4 V; Power density: ~ 457 μW cm^−3^	Advantages: Significant output enhancement achieved with ultralow filler loading; self-powered, ideal for emergency event detection. Limitations: Long-term impact fatigue not fully evaluated	Fall detection: impact recognition with SOS alert transmission	[263]
PVDF nanoyarn 3D textile	Conjugate electrospinning and 3D weaving; Wi-Fi/4G wireless transmission	Tensile strength: 46 MPa; Wet-state sensitivity: 3.95 V kPa^−1^; Durability: 20,000 cycles	Advantages: Mechanical strength far exceeding typical flexible piezoelectric devices; sweat enhances rather than degrades output; breathable and washable textile platform Limitations: Lower sensitivity in dry state; complex structure increases fabrication cost	Motion monitoring: joint movement and posture; Pulse and blood-pressure trends; Smart textile-based alarms	[247]
PANI/MWCNTs	In situ polymerization and printing; Wi-Fi-based cloud connection	NH_3_ detection limit: 0.3 ppm; Response/recovery: 21/191 s	Advantages: High sensitivity to breath ammonia relevant to metabolic status; maintains performance after bending Limitations: Long recovery time limits high-frequency monitoring	Breath analysis: NH_3_ concentration for kidney-function assessment	[264]
PANI-based multi-ion sensors	Sputtered electrodes with microfluidics; Wi-Fi smartphone connection	pH sensitivity: 54.7 mV pH^−1^; Na^+^/K^+^: ~ 59 mV decade^−1^	Advantages: Near-Nernstian response with simultaneous multi-parameter sensing; real-time wireless readout Limitations: Limited stretchability of the substrate; long-term sweat contamination may induce signal drift	Sweat monitoring: pH, Na^+^, and K^+^ concentrations	[265]
PEDOT:PSS (GOPS + CYTOP modified)	Fully printed fabrication; wireless body-temperature platform	TCR: 0.77% °C^−1^; Operating range: 25–50 °C	Advantages: Humidity stability considerably superior to unmodified PEDOT:PSS; suitable for continuous body-temperature monitoring Limitations: Not self-powered; detection range mainly limited to physiological temperatures	Body-temperature monitoring: skin temperature variation (fever and recovery)	[266]

### MXene Materials in Polymeric Matrices

MXenes (e.g., Ti_3_C_2_T_x_) are 2D transition metal carbides and nitrides characterized by high metallic conductivity, a unique layered architecture, and abundant surface functional groups. In flexible sensing platforms, their 2D structure permits stable hybridization with elastomeric matrices, while their highly active surfaces facilitate efficient electrochemical transduction and electromechanical responses. Rather than directly powering RF modules or acting as autonomous system nodes, MXenes provide a highly sensitive, conductive, and functionalizable material foundation for capturing both biomechanical and biochemical signals with minimal signal drift [267–269].

**Fig. 24:**
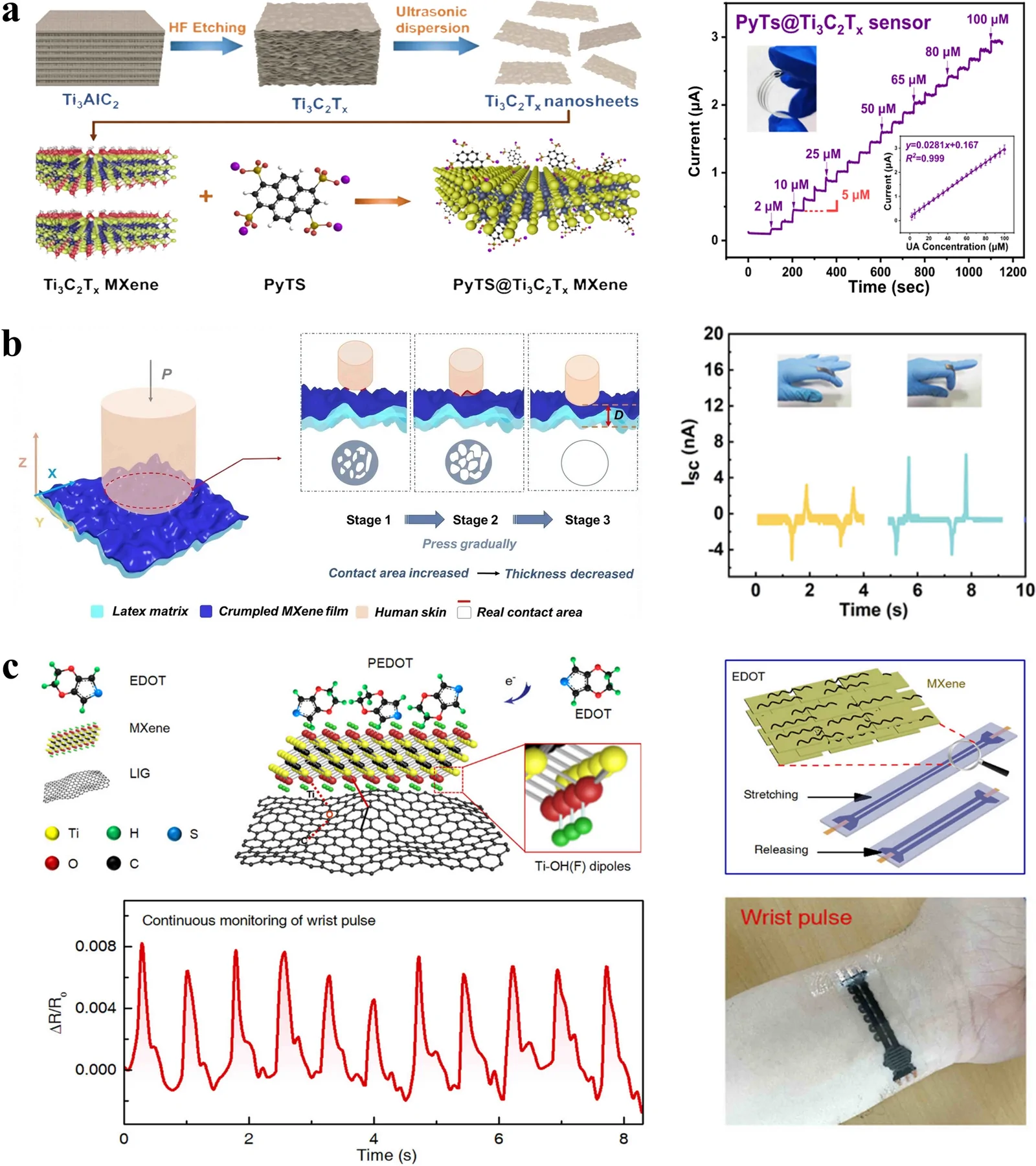
**a** Flexible electrochemical biosensor based on a functionalized MXene composite for real-time monitoring of uric acid in sweat [270]. Reproduced with permission. Copyright 2024, Analytical Chemistry. **b** Self-powered flexible sensor based on a reconfigurable wrinkled Ti_3_C_2_T_x_ MXene structure, enabling real-time monitoring of neck bending and finger joint motion [271]. Reproduced with permission. Copyright 2022, Nano Energy. **c** Flexible multimodal sensor constructed from a MXene/EDOT composite, allowing simultaneous monitoring of human motion, skin temperature, and electrocardiographic signals [272]. Reproduced with permission. Copyright 2022, npj Flexible Electronics

Recent developments highlight the versatility of MXenes across multimodal detection. For biochemical tracking, Chen et al. [270] (Fig. 24a) developed a functionalized MXene composite (PyTS@Ti_3_C_2_T_x_) integrated into a microfluidic sweat-sampling system. The specific surface modification considerably enhanced the catalytic efficiency for uric acid electrooxidation, enabling real-time, non-enzymatic monitoring during aerobic exercise. For physical sensing, Cao et al. [271] (Fig. 24b) fabricated a self-powered sensor using a reconfigurable wrinkled Ti_3_C_2_T_x_ film. This wrinkled architecture increased the effective contact area and accommodated large structural rearrangements, providing high sensitivity for capturing dynamic motions like neck and finger joint bending. Advancing multimodal capabilities, Zhang et al. [272] (Fig. 24c) combined MXene with the conductive polymer EDOT. The resulting conductive network established efficient charge transport pathways, allowing the simultaneous capture of human motion, skin temperature variations, and high-quality ECG signals with considerably lower skin-contact impedance than standard clinical gel electrodes. Besides, Li et al. [273] successfully developed a self-powered bioelectronic textile with an interlocking structure by coupling 2D MXene and 1D AgNWs. The core breakthrough of this work is that this multi-material-coupled architecture not only effectively prevents the oxidation of conductive materials but also maintains stable electrical output under 120% stretching and after 4000 loading cycles. This device enables precise real-time monitoring of physiological signals such as pulse, respiration, and joint movements.

Despite their compelling detection capabilities, the practical application of MXenes in wearable systems is severely restricted by their intrinsic chemical instability. They are highly vulnerable to oxidative degradation when exposed to ambient air or aqueous environments (such as sweat). Crucially, from a materials and manufacturing perspective, this oxidation-induced structural degradation and the resulting unstable contact interfaces introduce severe electrical noise and signal distortion. This rapidly leads to baseline drift and compromises data reliability during continuous, long-term monitoring. To ensure the long-term reliability required for wireless data acquisition, future research must prioritize anti-oxidation strategies. Key directions include implementing surface encapsulation techniques to physically block oxygen and moisture, constructing heterogeneous hybrid systems to reinforce structural robustness, and utilizing functional modifications (e.g., with ionic liquids or specific polymers) to stabilize the conductive network against environmental degradation.

## Conclusions and Outlook

### Conclusions

Polymer-based flexible wireless sensors are catalyzing a fundamental shift in physiological data acquisition, moving health monitoring from sporadic, clinic-bound measurements to uninterrupted, human-integrated observation. Convergent advances in deformable electronics, molecularly tailored materials, sub-milliwatt radios, and adaptive signal fusion now permit thin, skin-like devices to continuously translate cardiovascular, respiratory, metabolic, wound-healing, and kinematic information into secure digital streams. These platforms eliminate the sampling gaps inherent in conventional spot checks while preserving natural mobility, thereby establishing a non-invasive, comfort-centric route to longitudinal health analytics.

This review systematically surveys recent advances in polymer-based flexible wireless sensors for health monitoring from the perspective of coordinated sensing response mechanisms and wireless system integration. Centered on the requirements of physiological signal acquisition and wireless transmission, an integrated analytical framework encompassing response mechanisms, system functionalities, and application scenarios is established, highlighting the synergistic interactions among different technological components under practical monitoring conditions.

First, from the standpoint of sensing response mechanisms, this review summarizes the roles and characteristics of various physical and chemical transduction modes in physiological signal detection, and analyzes how response speed, dynamic range, and long-term stability influence the overall performance of wireless health-monitoring systems. On this basis, common wireless communication schemes and energy management strategies are systematically reviewed with respect to wireless transmission and power supply, and their suitability is discussed in terms of power consumption control, link stability, and system-level integration.

Considering the operational requirements of wireless flexible sensing systems, the review further examines the application of data preprocessing techniques and lightweight machine learning approaches in physiological signal analysis, outlining their roles in enhancing signal reliability and analytical efficiency. Subsequently, fabrication methods for flexible devices are summarized, with emphasis on the characteristics of different processes in terms of structural uniformity, scalability, and compatibility with system integration. Building upon these analyses, the applications of different materials in wireless flexible health monitoring are comparatively reviewed, with particular attention to their performance in sensitivity, mechanical compliance, signal stability, and suitability for long-term monitoring.

Despite significant progress, the translation of polymer-based flexible wireless sensors from laboratory-scale prototypes to long-term wear, deep-tissue monitoring, and clinical deployment remains challenged by multiple technical bottlenecks. These include limited energy supply, insufficient wireless link robustness, constrained data processing capabilities, inadequate long-term biocompatibility, and the lack of well-established commercialization pathways. Addressing these challenges requires a holistic, system-level perspective to distill key issues and define practically implementable research directions.

### Challenges and Outlook

**Fig. 25:**
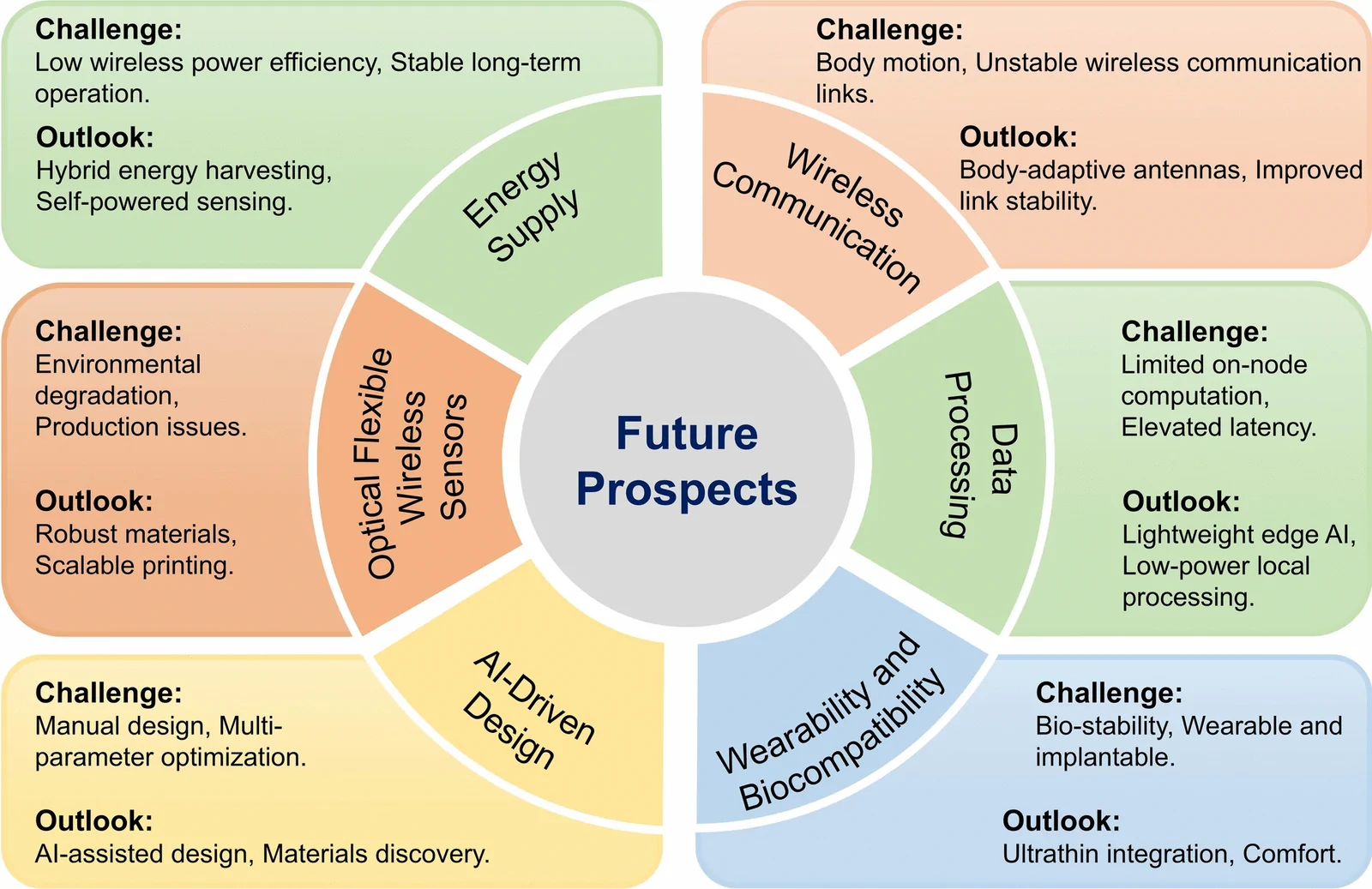
Conceptual roadmap illustrating future development directions of polymer-based flexible wireless sensors for health monitoring. The roadmap highlights the coordinated design and integration of energy-harvesting power systems, human-adaptive wireless communication, edge-intelligent data processing, long-term wearable and biocompatible device architectures, AI-assisted design frameworks, commercialization pathways and emerging opportunities in optical wireless sensing, aiming to enable clinically relevant, highly reliable, and scalable wireless flexible sensing platforms for diverse physiological environments and medical application scenarios

As shown in Fig. 25, a conceptual roadmap for the development of polymer-based flexible wireless sensors is proposed, outlining the key directions from sensor design to clinical applications. This roadmap is intended to advance health monitoring sensor platforms with clinical relevance, high reliability, and scalability.

#### Energy Supply and Energy Autonomy

The continuous operation of polymer-based flexible wireless sensors for health monitoring is highly dependent on a stable energy supply. Current mainstream solutions include RF energy transfer, miniaturized energy storage units, and energy-harvesting technologies. Among these, far-field RF or microwave powering has attracted widespread attention due to its advantages of being non-contact and capable of long-distance transmission. However, in practical physiological environments, the absorption of electromagnetic waves by human tissues, environmental scattering, and dynamic impedance mismatch can considerably reduce energy transfer efficiency, making it difficult to maintain available power at a stable level over extended periods. This has become a key limiting factor for the long-term operation of flexible wireless sensor systems.

The design of future energy systems requires synergistic optimization at the material, structural, and circuit levels. On the one hand, the incorporation of high-Q flexible rectifying antennas, low-loss conductive networks, and stretchable antenna topologies can enhance RF-DC conversion efficiency to a certain extent. On the other hand, adaptive impedance matching and dynamic power management circuits help mitigate energy fluctuations caused by changes in human posture and environmental disturbances. Furthermore, multi-source energy-harvesting and integration strategies hold significant development potential. By synergistically utilizing ambient RF signals, thermoelectric effects, piezoelectric/triboelectric effects, and the chemical potential of sweat, complementary operation across different energy channels can be achieved, thereby reducing the system’s reliance on conventional batteries.

#### Wireless Link Stability and Interference Mitigation

In wireless flexible health-monitoring systems, stable and reliable data transmission is fundamental for continuous monitoring and real-time feedback. Commonly used wireless communication technologies include BLE, UWB, and NFC, which offer advantages in low-power and short-range communication. However, from a materials and manufacturing perspective, signal distortion and noise present significant challenges to reliable data acquisition and transmission. Specifically, the mismatch in elastic modulus between flexible device materials and human skin often leads to severe motion artifacts during body movements. Furthermore, the instability of electrode–skin contact impedance under continuous dynamic deformation introduces baseline wandering and electrical noise. These material-level issues are compounded by the complex electromagnetic properties of the human body, physical motion during wear, and environmental electromagnetic interference, which can dynamically alter the resonant frequency and degrade the performance of flexible antennas. Consequently, these factors combined can lead to multipath fading, signal blockage, packet loss, and increased communication latency, or even link disruption, thereby considerably affecting overall system performance.

Enhancing wireless link stability requires coordinated improvements at both the hardware and communication strategy levels. At the hardware level, directional control of flexible antennas, optimization of polarization modes, and layout design based on the dielectric properties of the human body can improve signal radiation and reception efficiency. Combined with beamforming and adaptive channel compensation algorithms, multipath and scattering effects can be dynamically corrected in real time under varying human postures. At the system level, hybrid communication architectures that integrate multiple modalities and protocols can leverage complementary features across different standards, enhancing link redundancy and reliability in complex usage scenarios.

#### Lightweight Intelligent Computing, Data Reliability, and Security

Due to the power and computational limitations of flexible sensing nodes, complex signal processing and intelligent analysis tasks in current flexible wireless sensor systems largely rely on cloud-based computation. While this architecture can improve analytical accuracy to some extent, it also introduces communication latency, increased energy consumption, and potential data privacy risks, limiting the system’s real-time performance and long-term applicability.

Future research should focus on enhancing on-node intelligence and addressing the practical bottlenecks of AI deployment. First, there is a fundamental conflict between AI model complexity and the resource-constrained hardware of flexible nodes. To manage local energy budgets, it is imperative to develop lightweight architectures using model pruning, quantization, and knowledge distillation, combined with power-aware edge computing. Second, AI performance heavily depends on data quality. Wearable data are frequently corrupted by noise, environmental interference, and motion artifacts, necessitating robust AI-based denoising to improve the SNR. Finally, generalization remains a critical hurdle, as clinical reliability is often compromised by substantial inter-individual biophysical differences. Combined with edge computing and few-shot learning strategies [274], adaptive threshold adjustments tailored to unique patient characteristics can considerably improve model adaptability. Furthermore, incorporating distributed AI architectures, end-to-end encryption, and regulatory considerations is critical to preventing information leakage during AI training and inference, ensuring data privacy [275].

#### Device Miniaturization, Biodegradability, and Long-Term Wearability

Device miniaturization and long-term stability are critical prerequisites for the practical application of polymer-based flexible wireless sensors, particularly for prolonged wearable or implantable monitoring. Currently, many systems still face limitations in terms of device volume, encapsulation thickness, and long-term mechanical and electrical stability, making it challenging to achieve reliable operation over extended periods while maintaining user comfort.

To meet practical application requirements, future development should leverage advanced fabrication and packaging technologies to achieve highly integrated designs at the material and structural levels. Flexible chips, 3D/4D printing, and heterogeneous integration packaging offer feasible routes to reduce device size and thickness. In this context, silicon nano-membranes (SiNMs) have emerged as a critical material platform for driving extreme miniaturization. Due to their ultra-thin geometry (typically in the nanometer to micrometer range), SiNMs effectively overcome the intrinsic brittleness and rigidity of bulk silicon while preserving its superior semiconducting properties, such as high carrier mobility and excellent long-term stability. Integrating SiNMs with flexible or stretchable substrates enables the development of high-performance, skin-integrated sensors with exceptional accuracy and a minimal physical footprint. Furthermore, the incorporation of liquid metal interconnects, nanocomposite conductive materials, and highly biocompatible polymers can enhance mechanical compliance while maintaining electrical performance. For implantable applications, the development of biodegradable flexible materials and low-immunogenic interfaces will play a key role in ensuring long-term biocompatibility [276].

#### AI-Driven Co-Design of Materials, Structures, and Systems

As the complexity of flexible wireless sensor systems continues to increase, traditional experience-driven approaches for material selection and structural design increasingly reveal limitations in efficiency and scalability. The strong coupling between material properties, structural geometry, and system functionality makes it difficult to achieve overall performance improvement through single-layer optimization.

AI offers new tools for the co-design of materials, structures, and systems. Machine learning and GNN approaches can predict the molecular structure, mechanical behavior, and electrical performance of functional materials, supporting inverse design targeted toward specific performance objectives. Generative AI models can automatically construct conductive networks, electrode geometries, and deformable antenna layouts, accelerating the structural optimization process. Furthermore, the integration of multi-physics simulation with AI-calibrated digital twin technologies enables virtual prediction of device lifetime, fatigue behavior, and system-level performance, thereby shortening development cycles and reducing experimental costs. Importantly, similar to the data dependency and generalization challenges in processing physiological signals (detailed in Sect. 6.2.3), AI-driven material co-design is currently bottlenecked by the lack of high-quality, standardized material databases and the difficulty of generalizing predictive models across distinct material systems.

#### Commercialization Pathways and Emerging Opportunities in Optical Wireless Sensing

Beyond signal transduction, optical communication represents a highly promising frontier for wireless data transmission within these systems. While conventional setups rely heavily on RF technologies, flexible optoelectronic components (e.g., flexible light-emitting diodes and photodetectors) offer unique advantages for data relay, including substantial bandwidth, low latency, and intrinsic immunity to electromagnetic interference (EMI). This anti-interference characteristic makes them indispensable in specialized environments, such as continuous vital sign monitoring in strong magnetic fields or highly secure data transfer applications. Additionally, combining advanced optical nanomaterials and these emerging optical communication protocols is essential for transitioning these systems from experimental prototypes to practical, user-friendly health monitoring platforms.

The current bottleneck for commercializing polymer-based flexible wireless sensors lies in the lab-to-market gap, where environmental degradation (e.g., sweat and encapsulation failure), power limitations, and manufacturing inconsistencies hinder long-term reliability. For optical flexible sensors specifically, challenges involve miniaturizing photonic readout systems and mitigating signal drift caused by ambient light and temperature fluctuations. Consequently, addressing these challenges is pivotal for clinical translation, unlocking the full potential of optical sensors in specialized settings like MRI rooms where conventional RF-based electronics face severe limitations.
